# Selective Adsorption of Oxygen from Humid Air in a Metal–Organic Framework with Trigonal Pyramidal Copper(I) Sites

**DOI:** 10.1021/jacs.3c10753

**Published:** 2024-01-26

**Authors:** Kurtis M. Carsch, Adrian J. Huang, Matthew N. Dods, Surya T. Parker, Rachel C. Rohde, Henry Z. H. Jiang, Yuto Yabuuchi, Sarah L. Karstens, Hyunchul Kwon, Romit Chakraborty, Karen C. Bustillo, Katie R. Meihaus, Hiroyasu Furukawa, Andrew M. Minor, Martin Head-Gordon, Jeffrey R. Long

**Affiliations:** † Institute for Decarbonization Materials, University of California, Berkeley, Berkeley, California 94720, United States; ‡ Department of Chemistry, University of California, Berkeley, Berkeley, California 94720, United States; § Department of Chemical and Biomolecular Engineering, University of California, Berkeley, Berkeley, California 94720, United States; ∥ Materials Sciences Division, Lawrence Berkeley National Laboratory, Berkeley, California 94720, United States; ⊥ National Center for Electron Microscopy, Molecular Foundry, Lawrence Berkeley National Laboratory, Berkeley, California 94720, United States; # Department of Materials Science and Engineering, University of California, Berkeley, Berkeley, California 94720, United States

## Abstract

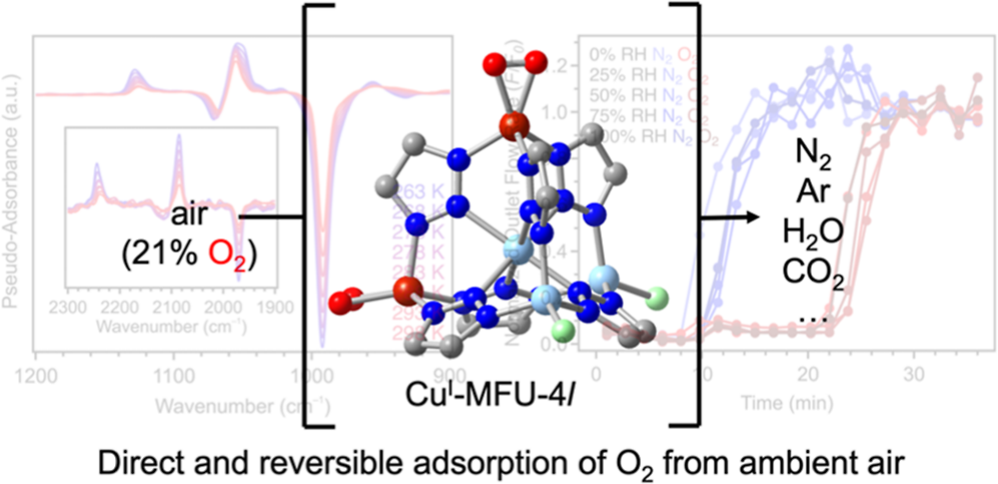

High or enriched-purity O_2_ is used in numerous industries and is predominantly produced from the cryogenic distillation of air, an extremely capital- and energy-intensive process. There is significant interest in the development of new approaches for O_2_-selective air separations, including the use of metal–organic frameworks featuring coordinatively unsaturated metal sites that can selectively bind O_2_ over N_2_*via* electron transfer. However, most of these materials exhibit appreciable and/or reversible O_2_ uptake only at low temperatures, and their open metal sites are also potential strong binding sites for the water present in air. Here, we study the framework Cu^I^-MFU-4*l* (Cu_*x*_Zn_5–*x*_Cl_4–*x*_(btdd)_3_; H_2_btdd = bis(1*H*-1,2,3-triazolo[4,5-*b*],[4′,5′-*i*])dibenzo[1,4]dioxin), which binds O_2_ reversibly at ambient temperature. We develop an optimized synthesis for the material to access a high density of trigonal pyramidal Cu^I^ sites, and we show that this material reversibly captures O_2_ from air at 25 °C, even in the presence of water. When exposed to air up to 100% relative humidity, Cu^I^-MFU-4*l* retains a constant O_2_ capacity over the course of repeated cycling under dynamic breakthrough conditions. While this material simultaneously adsorbs N_2_, differences in O_2_ and N_2_ desorption kinetics allow for the isolation of high-purity O_2_ (>99%) under relatively mild regeneration conditions. Spectroscopic, magnetic, and computational analyses reveal that O_2_ binds to the copper(I) sites to form copper(II)–superoxide moieties that exhibit temperature-dependent side-on and end-on binding modes. Overall, these results suggest that Cu^I^-MFU-4*l* is a promising material for the separation of O_2_ from ambient air, even without dehumidification.

## Introduction

**Figure 1:**
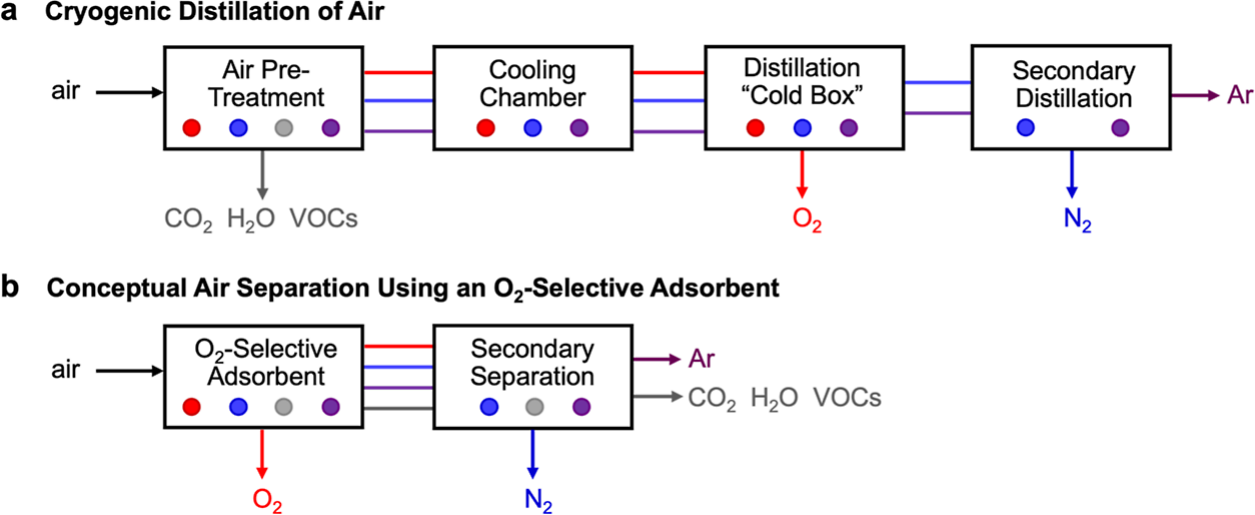
(a) Simplified process flow diagram of current industrial air separation, entailing air pretreatment to remove condensable volatiles, followed by cryogenic distillation to isolate O_2_. Note that the initial pretreatment step often entails adsorption along with its associated unit operations, which are not shown in detail here. A subsequent secondary distillation is used to separate N_2_ and Ar. VOC denotes volatile organic compounds, which present safety issues due to potential uncontrolled oxidations with liquid oxygen in the distillation “cold box”.^18,19^ (b) More desirable adsorbent-based air separation would entail the direct removal of O_2_ from untreated air at ambient temperatures, followed by secondary separation to purify N_2_ and Ar.

Enriched- or high-purity O_2_ is a critical commodity in the medical, manufacturing, and aerospace industries and for the production of feedstock chemicals such as ethylene oxide and phthalic anhydride.^1,2^ The vast majority of O_2_ is produced from the cryogenic distillation of air.^3,4^ This energy-intensive, multistep process involves the compression and pretreatment of air to remove volatile organic compounds, water, and CO_2_, and then the resulting gaseous mixture—predominantly O_2_, N_2_, and Ar is expanded and cooled to cryogenic temperatures upon passing through a series of heat exchangers, before being fed into distillation columns where O_2_ is separated from N_2_ and Ar. A simplified illustration of the basic steps required for the cryogenic distillation of air is shown in Figure 1a. Ultimately, while cryogenic distillation is the most mature and widely used technology for air separations, there is significant interest in identifying more energy-efficient and scalable methods for isolating O_2_ from air.

The prospect of using O_2_-selective adsorbents for energy-efficient air separations has garnered research attention for decades,^4,5^ beginning with early studies of O_2_ binding in molecular cobalt(II) complexes.^6^ A renaissance in this area has occurred within the previous decade with the discovery that certain porous, microcrystalline metal–organic frameworks (MOFs) featuring coordinatively unsaturated iron(II),^7−9^ cobalt(II),^10−13^ and chromium(II)^14,15^ sites can bind O_2_*via* electron transfer mechanisms that give rise to excellent selectivities for O_2_ over typically redox-inactive N_2_. The reader is referred to two recent perspective articles published on this topic.^4,16^ Importantly, air separations using cation-exchanged zeolites that selectively adsorb N_2_ over O_2_ (and Ar) are already used in industry to supplement cryogenic distillation for applications where O_2_ purities <95% are sufficient (*e.g.*, for medicinal use). As such, in particular for medium to small-scale applications, infrastructure is in place that could in principle be adapted to implement separations technology using O_2_-selective adsorbents^3,4^

A porous adsorbent capable of selectively capturing O_2_ over N_2_ and the other components of air, such as water, could be used to produce high purity O_2_ from air in a process that requires no pretreatment^17^ (other than removal of particulate matter) and is thus in principle operationally simpler than cryogenic distillation or N_2_-selective adsorptive separations.^18,19^ An illustration of such a hypothetical process is given in Figure 1b, although we note that this is a conceptual diagram only, intended to highlight an idealized process flow for such an adsorbent. In principle, far less adsorbent would be required to treat a given quantity of air in such a process than would be needed for an equivalent air separation using an N_2_-selective zeolite, since the concentration of O_2_ (21%) in air is much less than the concentration of N_2_ (78%).^4^ Consequently, the capital and energy expenditures required for air separations using an O_2_-selective adsorbent could be significantly less than what is required for cryogenic distillation and current adsorptive separations.^4^ However, the majority of O_2_-selective MOFs studied to date adsorb appreciable O_2_ only at subambient temperatures^7−13,20^ or exhibit poor stability to repeated cycling.^14,15,21^ Additionally, these materials feature coordinatively unsaturated, Lewis acidic metal sites that can also serve as strong binding sites for water.^22,23^ Importantly, none of the corresponding studies has examined adsorbent O_2_ selectivity and capacity in the presence of water vapor, which is a non-negligible component of air.

**Figure 2:**
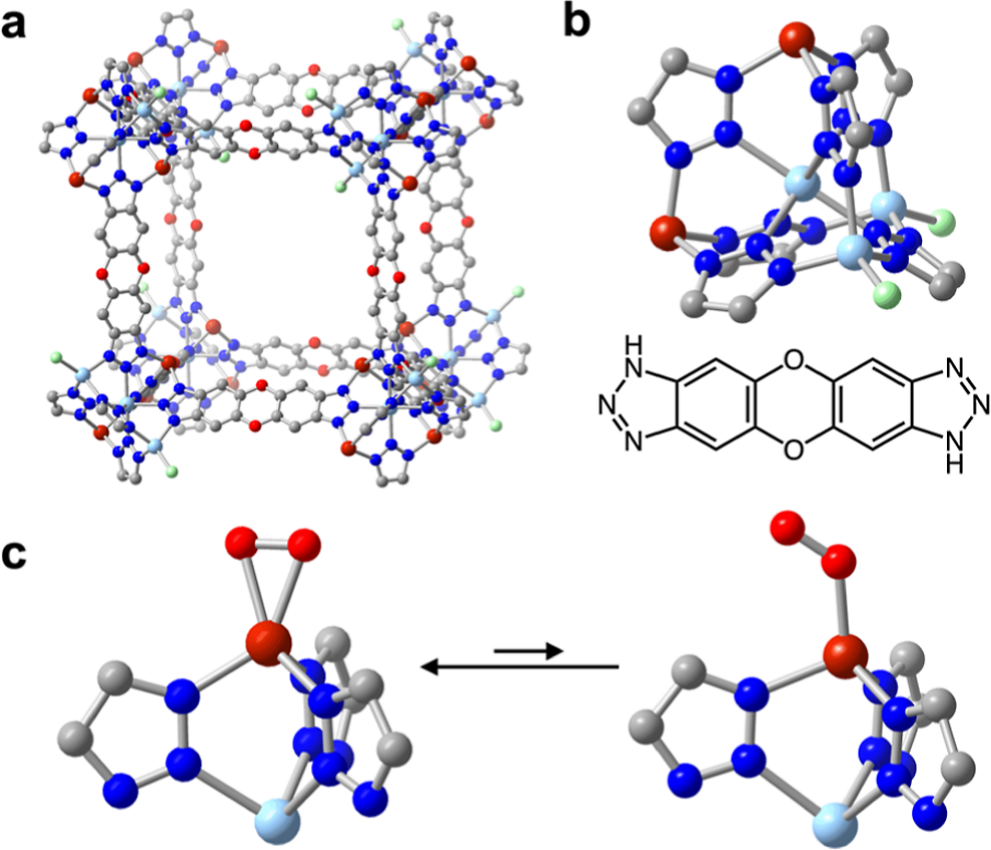
(a) Solid-state structure of Cu_2.4_-MFU-4*l* determined from Rietveld refinement of synchrotron powder X-ray diffraction data (Figure S50). The framework Cu_2.4_-MFU-4*l* was prepared *via* an optimized synthesis route involving the direct reaction of Zn_5_Cl_4_(btdd)_3_ with copper(I) chloride dimethylsulfide. Note that the Cu and Zn sites are disordered such that the aggregate ratio of Cu to Zn is 2.4 to 2.6 for the framework. (b) (Upper) Expanded view of a pentanuclear node in Cu_2.4_-MFU-4*l*. (Lower) Structure of the H_2_btdd linker. (c) Illustration of calculated structures for superoxide bound in a side-on and end-on fashion to copper(II) sites in the model pentanuclear cluster Cu_2_Zn_3_Cl_2_(bta)_6_ (ta^–^ = 1,2,3-benzotriazolate; see the Supporting Information for details). Vibrational spectroscopy analysis supports that O_2_ adsorbs in Cu_2.4_-MFU-4*l* to generate both side-on and end-on superoxide bound to copper(II), which are in temperature-dependent equilibrium (Figure 3). Brown, light blue, green, red, dark blue, and gray spheres represent Cu, Zn, O, N, Cl, and C atoms, respectively.

A noteworthy framework in the context of air separations is Cu^I^-MFU-4*l* (Cu_*x*_Zn_5–*x*_(Cl/OOCH)_4–*x*_(btdd)_3_; H_2_btdd = bis(1*H*-1,2,3-triazolo[4,5-*b*],[4′,5′-*i*])dibenzo[1,4]dioxin), which features pentanuclear cluster nodes consisting of a central octahedral zinc(II) ion coordinated to four peripheral metal ions either pyramidal copper(I) or tetrahedral zinc(II) (Figure 2a,b). Under ambient conditions, the copper(I) sites in the framework have been shown to strongly and reversibly bind O_2_,^24^ and the favorable calculated Δ*G*° of O_2_ binding at 298 K in this material suggests that it may be promising candidate for O_2_-selective adsorptive air separations.^4^ Additionally, in the context of hard–soft acid–base theory, we hypothesized that the intrinsic mismatch between the soft copper(I) ion and hard water molecule may render Cu^I^-MFU-4*l* selective for O_2_ even in the presence of water.^25^

Herein, we disclose that Cu^I^-MFU-4*l*, synthesized with new optimized procedures that result in higher Cu^I^ loadings, is able to reversibly adsorb O_2_ from air at ambient temperatures with excellent cyclability, even in the presence of water vapor. Variable-temperature *in situ* diffuse reflectance infrared Fourier transform spectroscopy (DRIFTS) and magnetic susceptibility data indicate that O_2_ binds at the copper(I) sites to form copper(II)–superoxide motifs and that side- and end-on superoxide binding modes are in equilibrium over a range of temperatures. We find that differences in the kinetics of desorption of O_2_ and N_2_ from the framework allow for the isolation of high-purity O_2_, providing a new approach to separate O_2_ directly from ambient air.

## Results and Discussion

### Materials Synthesis and Characterization

The framework Cu^I^-MFU-4*l* was initially synthesized following the literature protocol.^26^ In brief, Zn_5_Cl_4_(btdd)_3_ (MFU-4*l*)^24^ was treated with excess CuCl_2_ in *N*,*N*-dimethylacetamide under an inert atmosphere at 60 °C to give Cu^II^Cl-MFU-4*l* [Cu^II^_2.2_Zn_2.8_Cl_4_(btdd)_3_ based on inductively coupled plasma optical emission spectroscopy, ICP-OES]. Subsequent anion exchange with lithium formate monohydrate and thermolysis at 180 °C afforded Cu^I^-MFU-4*l* (Figure S38). When prepared using this route, Cu^I^-MFU-4*l* has been reported to contain a mixture of copper(I) and copper(II) ions.^27−29^ The copper(I) sites in this material are known to strongly bind H_2_,^24,26^ and therefore as a means of qualitatively estimating the number of these sites in Cu^I^-MFU-4*l*, we collected H_2_ adsorption isotherms at 77 K and pressures ranging from 0 to 1.2 bar (Figure S7). The material exhibits steep H_2_ uptake at low pressures and achieves a capacity of 1.3 mmol/g at 1 mbar, followed by more gradual uptake at higher pressures indicative of H_2_ physisorption. If all of the copper sites in the material were trigonal pyramidal copper(I), and assuming a 1:1 stoichiometry for H_2_ binding,^24^ we would expect a low-pressure uptake of approximately 1.9 mmol/g (based on the copper site stoichiometry determined for the Cu^II^-MFU-4*l* precursor from ICP-OES). From the measured uptake of 1.3 mmol/g at 1 mbar, we then estimate that ∼68% of the copper ions are exposed copper(I) sites.^30^ While estimates of copper(I) loading achieved in this way are qualitative (see Figure S7), we propose that the H_2_ uptake at 1 mbar may be a useful means of estimating and comparing copper(I) loading in Cu^I^-MFU-4*l* materials [see Table S2 for a comparison of reported copper(I) loadings in various Cu^I^-MFU-4*l* samples prepared in the literature and other qualitative approaches used to evaluate loadings].

With the goal of accessing a form of Cu^I^-MFU-4*l* featuring a greater number of copper(I) sites per node and therefore higher gas adsorption capacities, we sought to optimize the synthesis of this material. For simplicity, we denote Cu^I^-MFU-4*l* materials prepared *via* different routes simply as Cu_*x*_-MFU-4*l*, where *x* specifies the total number Cu sites per node as quantified by ICP-OES analysis of the copper(II) precursor (*e.g.*, the shorthand for Cu^I^-MFU-4*l* prepared *via* the literature route^24,26^ is Cu_2.2_-MFU-4*l*). Following extensive optimization, we found that treatment of Zn_5_Cl_4_(btdd)_3_ with 40 equiv of CuCl_2_ in anhydrous dimethyl sulfoxide at 60 °C yields a material with 2.7 Cu^II^ ions per pentanuclear node, based on energy-dispersive X-ray spectroscopy and ICP-OES (Figures S29 and S30). Two sequential additions of lithium formate monohydrate followed by a thermolysis sequence ending with heating at 250 °C afforded a material with the formula Cu_2.7_Zn_2.3_H_0.9_Cl_0.7_(btdd)_3_ (hereafter, Cu_2.7_-MFU-4*l*; see Section S2 of the Supporting Information for synthesis details and Figures S15, S29, S34, S37, and S38).

Analysis of H_2_ adsorption data collected at 77 K revealed that Cu_2.7_-MFU-4*l* adsorbs 2.1 mmol/g at 1 mbar of H_2_, nearly double the capacity measured for Cu_2.2_-MFU-4*l* under the same conditions (Figure S8; Table S2). From this uptake, we estimate that approximately 89% of the copper sites in the material are copper(I), which is one of the highest levels of copper(I) incorporation reported for Cu^I^-MFU-4*l* to date. As validation of this approach, we also conducted an experiment where we dosed a tared sample of activated Cu_2.7_-MFU-4*l* with 50 mbar of CO at 298 K to saturate the copper(I) sites^24^ and then evacuated the sample to remove physisorbed CO. The resulting sample mass increased by 5.8(1) wt %, corresponding to a copper(I) loading of 2.4(1) per node, consistent with the copper(I) loading estimated from the H_2_ adsorption data (see Table S14). Analysis of 77 K N_2_ adsorption data revealed that Cu_2.7_-MFU-4*l* has a high BET surface area of 4160(40) m^2^/g (Figure S1) that exceeds previously reported values for Cu^I^-MFU-4*l* (347–4000 m^2^/g, see Table S2).^24,26,28,31−34^ Thermal decomposition profiles collected under dry N_2_ and O_2_ revealed that the material is stable until approximately 400 and 280 °C, respectively, under these gases (Figure S16). We found it is also possible to prepare Cu^I^-MFU-4*l via* the abovementioned route using hydrated CuCl_2_ in dimethyl sulfoxide without the exclusion of air or water (see Section S2 of the Supporting Information for details). The resulting material exhibits a similarly high H_2_ capacity of 1.9 mmol/g at 77 K and 1 mbar (Figure S9).

In our optimization of the synthesis of Cu^I^-MFU-4*l*, we also found that treatment of Zn_5_Cl_4_(btdd)_3_ with copper(I) chloride dimethylsulfide in acetonitrile at 25 °C and activation of the resulting framework at 300 °C affords Cu_2.4_Zn_2.6_Cl_1.6_(btdd)_3_ (Cu_2.4_-MFU-4*l*; see Figures 2a,b, S31, S35, and S36). The H_2_ adsorption capacity measured for this material at 77 K and 1 mbar is 2.0 mmol/g, consistent with the theoretical capacity assuming binding of one molecule of H_2_ per Cu(I) site in the material (2.0 mmol/g) (Figure S7; a consistent loading was also determined from analysis of CO uptake in the material, see Table S14). Advantageously, this approach affords access to Cu^I^-MFU-4*l* with a more well-defined formula and in fewer synthetic steps than the materials accessed *via* copper(II) substitution and autoreduction. The BET surface area of Cu_2.4_-MFU-4*l* is 3820(30) m^2^/g (Figure S1), which is slightly lower than the surface area measured for Cu_2.7_-MFU-4*l* and consistent with the presence of only chloride capping ligands, in contrast to the mixture of chloride and smaller hydride ligands in Cu_2.7_-MFU-4*l*. For all O_2_, N_2_, and Ar isotherm data collection (see below), we employed Cu_2.7_-MFU-4*l* based on its slightly higher estimated copper(I) loading, while Cu_2.4_-MFU-4*l* was used for spectroscopic analyses due to its greater homogeneity of copper ions and coordinated anions.^35^

### Structural, Spectroscopic, and Computational Characterization of O_2_ Binding

As noted above, Cu^I^-MFU-4*l* is known to reversibly bind O_2_ at room temperature,^24^ and a recent investigation of O_2_ binding in Cu^I^-MFU-4*l* using *in situ* Cu L_2,3_-edge near-edge X-ray absorption fine structure spectroscopy and time-dependent density functional theory revealed that O_2_ adsorption is accompanied by significant electron transfer from copper(I) to O_2_ with partial oxidation of the copper ion.^27^ We sought to better understand the nature of the binding of the binding of the O_2_ species in this material using a suite of structural, spectroscopic, and computational analyses. Dosing microcrystalline Cu_2.4_-MFU-4*l* with 8 mbar of O_2_ at 195 K resulted in a rapid color change from off-white to pink, indicative of a change in the copper oxidation state. Analysis of powder X-ray diffraction data collected for Cu_2.4_-MFU-4*l* at 195 K before and after dosing with 8 mbar of O_2_ revealed a shift in the peak positions to higher 2θ values with gas dosing, while dosing with higher O_2_ pressures of 109 and 1005 mbar did not lead to further changes in the peak positions (Figure S45).

Pawley fits against the diffraction data for activated Cu_2.4_-MFU-4*l* collected under vacuum and the sample dosed with 8 mbar of O_2_ revealed a unit cell contraction upon O_2_ binding from 31.2090(14) to 31.0044(3) Å (cubic space group *Fm*3̅*m*, see Figures S42 and S43), while a smaller unit cell contraction was characterized upon dosing the material with 9 mbar of N_2_ at 195 K [from 31.2090(14) to 31.0997(11) Å, Figure S44]. These results suggest a greater perturbation of the local electronic structure around the copper(I) ions upon O_2_ binding *versus* N_2_ binding, and the decrease in unit cell parameter upon O_2_ dosing is consistent with the shortening of the metal–ligand bonds due to copper oxidation. Rietveld refinement of the diffraction data collected for activated Cu_2.4_-MFU-4*l* revealed a structure consistent with that reported previously based on neutron powder diffraction data (Figure 2a,b).^26^ Rietveld refinement of the diffraction data for O_2_-dosed Cu_2.4_-MFU-4*l* using the structure of the activated framework as a starting model revealed electron density above the copper sites that was refined as O_2_, yielding an occupancy of 2.8(4) molecules per node (Figures S47 and S48). However, the structural disorder of the O_2_ motif about the *C*_3_ axis as well as the crystallographic superposition of the zinc and copper ions preclude meaningful commentary on structural metrics or the nature of O_2_ binding.

**Figure 3:**
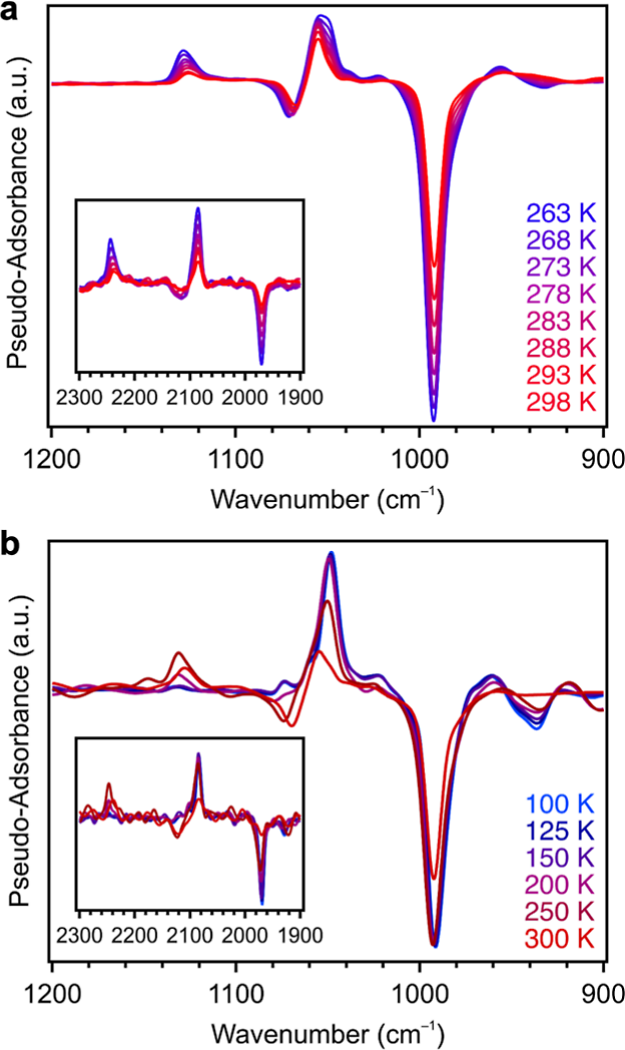
(a) Difference spectra obtained by subtracting DRIFTS data collected upon warming (263 to 298 K) a sample of Cu_2.4_-MFU-4*l* dosed with O_2_ from the corresponding data for Cu_2.4_-MFU-4*l* dosed with ^18^O_2_. (b) Difference spectra generated by subtracting DRIFTS data collected upon warming (100 to 300 K) a sample of Cu_2.4_-MFU-4*l* dosed with ^18^O_2_ (negative features) from the corresponding data for Cu_2.4_-MFU-4*l* dosed with O_2_ (positive features). Peaks corresponding to a secondary, less activated superoxide species appear at 200 K. The lower and higher energy features in both sets of spectra are assigned to superoxide species bound to copper(II) in a side-on and end-on fashion, respectively. Insets depict superoxide overtones.

We turned to variable-temperature *in situ* DRIFTS to further investigate the nature of the binding of O_2_ in Cu_2.4_-MFU-4*l*. Dosing the material with up to 1 bar of O_2_ at 300 K resulted in clear changes in the fingerprint region (600 to 1400 cm^–1^) (Figure S35). To elucidate the features arising from bound O_2_, we conducted identical experiments in which Cu_2.4_-MFU-4*l* was dosed at 263 K with 8 mbar of either natural abundance O_2_ (99.8% ^16^O_2_) or ^18^O_2_ (97 atom % ^18^O). Spectra were then collected at 5 K intervals as the sample was warmed from 263 to 298 K. A set of difference spectra generated by subtracting the ^18^O_2_-dosed spectra from the O_2_-dosed spectra are plotted in Figure 3a and clearly show positive and negative peaks corresponding to ^16^O_2_ and ^18^O_2_ vibrations, respectively. Intriguingly, two sets of features were generated upon O_2_ dosing: one pair at 1131 and 1051 cm^–1^ and another pair at 1073 and 993 cm^–1^ for the O_2_- and ^18^O_2_-dosed samples, respectively. The isotopic shifts for both features are consistent with predictions based on the simple harmonic oscillator model for O_2_ (peaks for ^18^O_2_-dosed material are predicted to be at 1066 and 990 cm^–1^). As such, we assign both sets of features to the O_2_ species.

To determine the origins of these resonances, we performed another set of experiments in which the activated framework was dosed with either 45 mbar O_2_ or ^18^O_2_ at 300 K, cooled to 100 K to saturate the copper(I) sites, and then spectra were collected as the sample was incrementally warmed to room temperature (Figure 3b). Interestingly, a reversible color change from pink to gray-brown was observed upon warming the sample from 100 K to room temperature (Figure S61). At 100 K, only the peaks at 1051 and 993 cm^–1^ were present, while above 200 K, the peaks at 1131 and 1073 cm^–1^ were also apparent. We assign the lower energy features to a superoxide-bound side-on (η^2^) to Cu^II^ (typically 970–1100 cm^–1^)^36−38^ and the higher energy features to a superoxide-bound end-on (η^1^) to Cu^II^ (typically 1100–1150 cm^–1^).^38−41^ Interestingly, this is to our knowledge the first example of a copper–O_2_ adduct where the coordinated O_2_ species exhibits a temperature-dependent equilibrium between binding modes. Dosing Cu_2.4_-MFU-4*l* with air at room temperature results in an additional resonance at 2242 cm^–1^ associated with N_2_ adsorption.^24^ This resonance is red-shifted from the Raman active mode of free N_2_ (2331 cm^–1^),^42^ indicating the copper(I) sites function as a weak π-donor for N_2_.^27^

The spin state of a copper–O_2_ adduct depends on the O_2_ binding mode. In particular, η^2^-O_2_ adducts have been found to possess a singlet ground state (*S* = 0),^38,43^ while η^1^-O_2_ adducts possess a triplet ground state (*S* = 1).^38,44^ As such, to support the assignments made based on the *in situ* DRIFTS data, variable-temperature dc magnetic susceptibility data were collected at 1 T for a sample of Cu_2.4_-MFU-4*l* dosed with O_2_ at 195 K (see the Supporting Information for details and Figures S49 and S50). Below 195 K, the magnitude of the molar magnetic susceptibility–temperature product (χ_M_*T*) is close to zero. However, as the material is warmed above 200 K, χ_M_*T* steadily increases to 0.25 emu·K/mol. Although the values of χ_M_*T* should be treated qualitatively due to challenges with the sample diamagnetic correction and desorption of O_2_ at higher temperatures, these data are consistent with an equilibrium between the *S* = 0 and *S* = 1 species, in which the higher spin state is at least partially accessed upon warming.

Density functional theory (DFT) calculations carried out on a pentanuclear cluster model of Cu^I^-MFU-4*l* further support an equilibrium between the O_2_ bound side-on and end-on to the copper sites in the framework (see Section S10 of the Supporting Information and Figure 2c). In particular, the η^2^-O_2_ binding mode was found to be favored based on electronic energies over the η^1^-O_2_ binding mode (Δ*E* = −65 *versus* −46 kJ mol^–1^, respectively). Additionally, the calculated O–O bond lengths for O_2_ bound in an η^2^ and η^1^ fashion are slightly longer than the O–O bond length for gaseous O_2_ (calculated 1.31 and 1.26 Å for O_2_*versus* 1.21 Å, respectively), consistent with electron transfer from copper(I) to O_2_ to form a copper(II)–superoxo (O_2_^•–^) moiety. Taken together, the structural, spectroscopic, and computational results support the formation of Cu^II^–O_2_^•–^ moieties upon O_2_ binding in Cu^I^-MFU-4*l*.^45^

### Investigation of O_2_, N_2_, Ar, and H_2_O Adsorption

**Figure 4:**
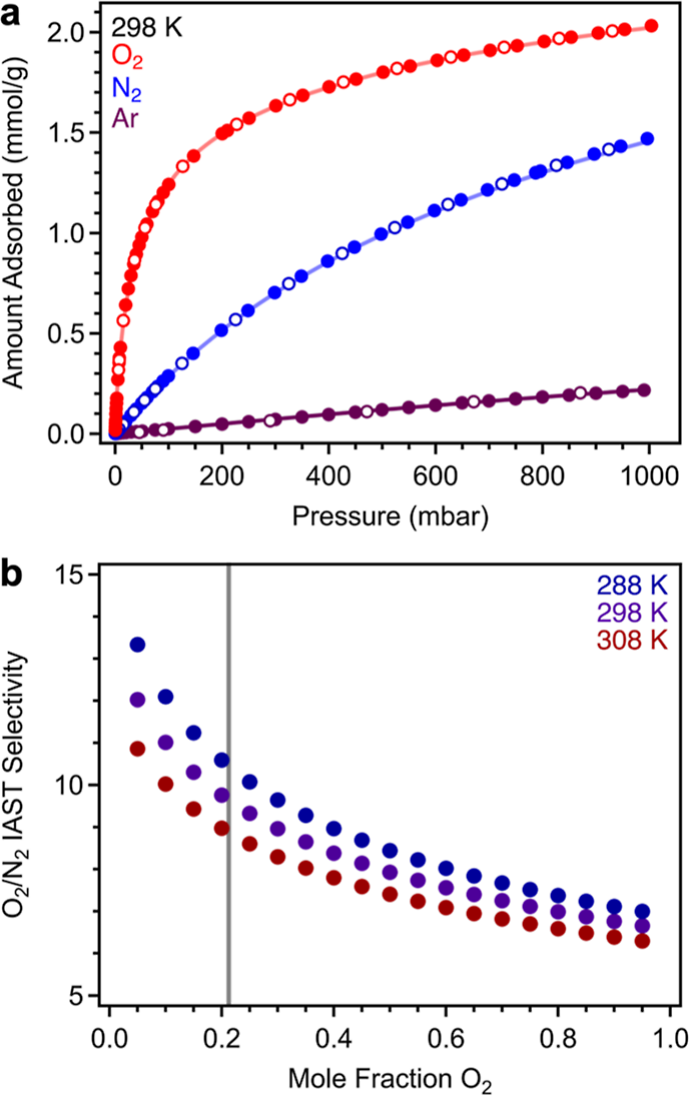
(a) Single-component O_2_, N_2_, and Ar adsorption (filled circles) and desorption (open circles) isotherm data collected at 298 K for Cu_2.7_-MFU-4*l*. Colored lines represent calculated curves obtained from simultaneous fitting of three single-component isotherms at different temperatures with either a dual-site Langmuir–Freundlich equation (N_2_, O_2_: 288, 298, and 308 K) or a single Langmuir–Freundlich equation (Ar: 170, 180, and 190 K). (b) Variable-temperature IAST selectivities calculated for a binary O_2_/N_2_ mixture. The O_2_ concentration in air (21%) is denoted with a vertical gray line.

Single-component O_2_, N_2_, and Ar isotherms were collected for Cu_2.7_-MFU-4*l* at 298 K and pressures ranging from 0 to 1 bar (Figure 4a). Consistent with binding of the O_2_ at the open copper sites, the material exhibits relatively steep uptake of the O_2_ at low pressures and achieves a capacity of 1.5 mmol/g at 210 mbar, the partial pressure of the O_2_ in air. This is the second highest O_2_ capacity reported for a MOF under these conditions (see Table S3), exceeded only by Cr_3_[(Cr_4_Cl)_3_(BTT)_8_]_2_ (Cr-BTT; BTT^3–^ = 1,3,5-benzenetristetrazolate), which exhibits a capacity of 2.2 mmol/g.^15^ However, Cr-BTT is not stable for repeated cycling with O_2_ under conditions relevant to uptake from ambient air, unlike Cu_2.7_-MFU-4*l* (see below). Oxygen uptake in Cu_2.7_-MFU-4*l* begins to level off at higher pressures, reaching a value of 2.0 mmol/g at 1 bar O_2_. Assuming that one O_2_ molecule binds at every copper(I) site, the theoretical capacity (excluding adsorption at secondary sites in the material) is 2.1 mmol/g. Considering that the capacity at 1 bar represents both chemisorption and physisorption, the experimental uptake suggests that not all copper(I) sites in this framework are saturated at this pressure. Nitrogen uptake in Cu_2.7_-MFU-4*l* is more gradual at low pressures, and the material adsorbs less N_2_ than does O_2_ over the entire pressure range (1.5 mmol/g at 1 bar). However, at the partial pressure of N_2_ in air (780 mbar), the material exhibits a N_2_ capacity of 1.3 mmol/g N_2_ that is only slightly less than the O_2_ capacity at 210 mbar. Finally, Cu_2.7_-MFU-4*l* adsorbs very little Ar at 298 K between 0 and 1 bar, and at 9 mbar, the partial pressure of Ar in air, the material adsorbs <0.01 mmol/g.

Variable-temperature Ar, O_2_, and N_2_ adsorption isotherms were collected to determine the enthalpies of adsorption in Cu_2.7_-MFU-4*l* (Figures S5 and S6). Low-temperature Ar isotherms were collected at 170, 180, and 190 K, and these data could be simultaneously modeled using the single-site Langmuir–Freundlich equation (Figure S6). In contrast, a dual-site Langmuir–Freundlich equation was needed to model O_2_ and N_2_ isotherms collected at 288, 298, and 308 K, consistent with primary gas binding at the copper sites and secondary interactions with the framework (Table S4). Using the fits to these data and the Clausius–Clapeyron equation, we determined O_2_, N_2_, and Ar adsorption enthalpies as a function of loading (Figure S13). The isosteric enthalpy of adsorption (Δ*H*_ads_) at low loadings of O_2_ is −56.8(1) kJ/mol, higher than the values determined for N_2_ [−38.9(4) kJ/mol] and Ar [−10.9(1) kJ/mol]. The enthalpy of Ar adsorption is consistent with weak adsorbate–framework interactions^46^ and remains essentially constant with loading, while the heats of adsorption for O_2_ and N_2_ gradually decline as the copper sites become saturated and secondary adsorption sites within the framework are occupied. The O_2_ and N_2_ adsorption enthalpies are consistent with previously reported isosteric enthalpy (heat) of adsorption in Cu^I^-MFU-4*l* [Δ*H*_ads_ = −*Q*_st_ = −52.6(6) and −41.6(6) kJ/mol, respectively; see Table S3 for heats of O_2_ and N_2_ adsorption reported for other relevant frameworks]^24^ and indicative of strong interactions with the open copper(I) sites. This result is consistent with the DRIFTS data and the electron transfer from copper(I) to O_2_.

Ideal adsorption solution theory (IAST)^47^ was used to predict the equilibrium adsorption behavior of Cu_2.7_-MFU-4*l* exposed to a binary O_2_/N_2_ mixture and extract O_2_/N_2_ adsorption selectivities as a function of O_2_ concentration (see the Supporting Information for details). For a binary mixture containing 21% O_2_, the O_2_/N_2_ selectivity is 10 at 298 K, which would correspond to 72% adsorbed phase purity (74% at 288 K; Figure 4b). However, it should be noted that one of the assumptions of IAST is that there is a homogeneous distribution of guests within the pores of the material,^48^ an assumption that is not likely to hold for Cu^I^-MFU-4*l*, where electron transfer is a driving force in O_2_ binding but not in the case of N_2_ binding. In a realistic scenario where O_2_ preferentially clusters around the copper(I) sites, IAST is expected to overestimate actual selectivity values.^48^ However, breakthrough analysis using dry and humid compressed air streams indicates that O_2_ does indeed bind selectively over N_2_ under dynamic conditions (see below). Interestingly, predicted N_2_/Ar and O_2_/Ar selectivities suggest that Cu_2.7_-MFU-4*l* may also be appropriate for removing N_2_ and O_2_ impurities in Ar purification (Figure S12).

In addition to a high capacity and selectivity for O_2_ over the other components of air, an ideal O_2_-selective adsorbent would exhibit robustness to humidity and a low affinity for water, thus potentially enabling air separations without the need for pretreatment to remove moisture. The Cu^I^-MFU-4*l* framework was previously reported to be air-stable, and in our hands, a sample of Cu_2.7_-MFU-4*l* was found to be robust to ambient air for 3 months, based on powder X-ray diffraction analysis (Figure S40). Furthermore, DRIFTS data collected for a sample of Cu_2.7_-MFU-4*l* exposed to the atmosphere at 300 K revealed the stable coordination of both N_2_ and O_2_ over the course of at least 10 h without any additional changes in color (Figure S36). A water isotherm collected for Cu_2.7_-MFU-4*l* at 298 K at relative humidity levels ranging from 2 to 80% revealed that the material has a low affinity for water (Figure S14), in contrast to the parent framework MFU-4*l*.^49^ Consistent with this result, DFT calculations predict an electronic energy of −27 kJ/mol for water adsorption at the copper(I) sites in the framework, indicating a weaker metal–adsorbate interaction compared to the binding of O_2_ or N_2_ to the exposed copper(I) site.^50^

Additionally, while the O_2_, N_2_, and Ar adsorption isotherms of Cu_2.7_-MFU-4*l* were completed within several hours, the completion of the water adsorption isotherm required approximately 50 h, indicative of sluggish water uptake kinetics. A small amount of hysteresis upon water desorption may be due to the formation of water clusters within the pores.^49,51^ Powder X-ray diffraction analysis of Cu_2.7_-MFU-4*l* following water adsorption/desorption isotherms collected at 298 and 308 K revealed that the material remains crystalline (Figure S38). These observations contrast the behavior of Cu_2.2_-MFU-4*l*, which exhibits substantial loss of crystallinity following water adsorption/desorption isotherms at 298 K. Finally, to probe the stability of Cu_2.7_-MFU-4*l* at 100% relative humidity under relevant conditions, we dosed a sample of the material with air presaturated with water for 30 min at 25 °C. The material was then reactivated (see Section S1.16 of the Supporting Information for details) and powder X-ray diffraction and O_2_ adsorption and desorption data were collected. The framework retained crystallinity, and the isothermal adsorption data are indistinguishable from that collected for the pristine material (see Figures S41 and S10, respectively), indicating excellent material stability.

### Adsorption/Desorption Cycling Performance under Dry Air

To gain initial insight into the cycling stability of Cu_2.7_-MFU-4*l* under more realistic O_2_ capture conditions, we performed thermogravimetric analysis adsorption/desorption cycling experiments by exposing a sample of the material to flowing dry air (10 min at 30 °C), followed by desorption under a simulated vacuum (Ar purge, 10 min at 30 °C). Remarkably, the material retained >99.9% of its total capacity over the course of 40 cycles, although we note that the weight change measured under these conditions reflects both adsorbed O_2_ and N_2_ (Figure S18). Adsorption/desorption cycling data were also collected under simulated temperature swing conditions by exposing the material to dry air (5 min at 30 °C), followed by desorption under an O_2_ purge at higher temperature (30 s at 100 °C). Under these conditions, incomplete desorption was observed, and a capacity of ∼95% (O_2_ and N_2_) was retained over 40 cycles (Figure S19). The slightly lower capacity relative to that measured under simulated pressure swing conditions is attributed to the highly oxidizing conditions used for desorption.

### Kinetics Measurements

**Figure 5:**
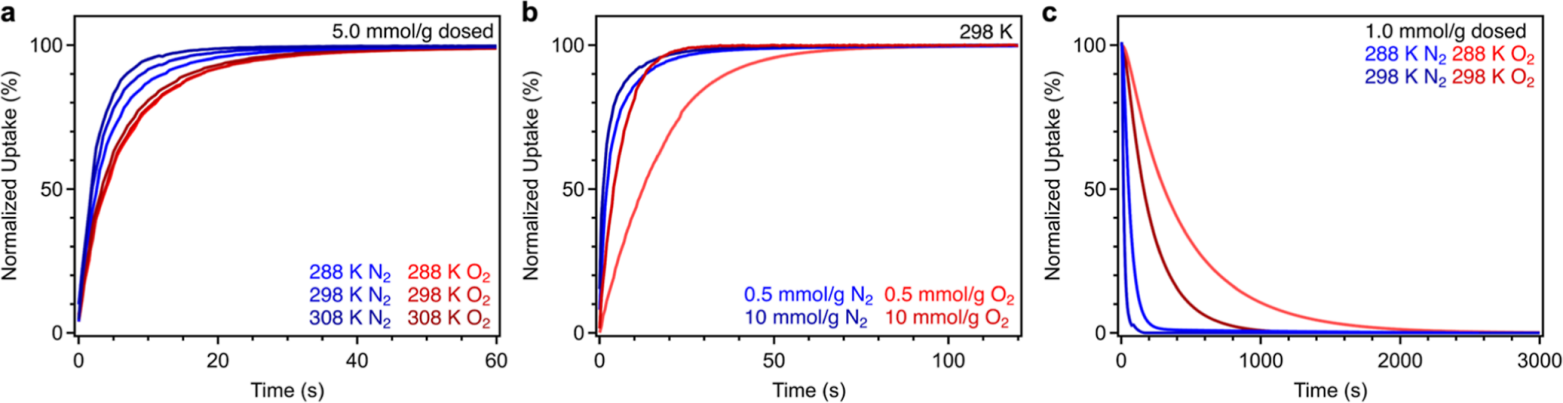
(a) Normalized adsorption kinetic traces for Cu_2.7_-MFU-4*l* dosed with 5.0 mmol/g N_2_ and O_2_ at 288, 298, and 308 K. Nitrogen adsorption and equilibrium in the material occurs slightly more rapidly than for O_2_ at all temperatures. (b) Higher initial dosing quantity for N_2_ and O_2_ minimizes differences in equilibration times seen at lower dosing concentrations. (c) Normalized O_2_ and N_2_ desorption kinetic traces collected for Cu_2.7_-MFU-4*l* following dosing at variable temperatures. Oxygen desorption is more gradual than N_2_ desorption, and this difference is enhanced at a lower temperature.

Adsorption and desorption kinetics are also critical factors to consider in assessing the utility of a candidate adsorbent. We investigated the kinetics of O_2_ and N_2_ adsorption and desorption in Cu_2.7_-MFU-4*l* at temperatures of 288, 298, and 308 K after dosing with initial quantities of each adsorbate (0.5, 1.0, 5.0, or 10.0 mmol/g) corresponding to equilibrated pressures ranging from approximately 3 to 330 mbar (see Table S5; these pressures reflecting the steep region of the single-component isotherms for each gas). Adsorption of O_2_ and N_2_ occurred rapidly in Cu_2.7_-MFU-4*l*, with pressure equilibration occurring within approximately 50 s or less in both cases and for all temperatures and dosing conditions (Figure S20). For both gases, the rate of adsorption increased with an increase in temperature, although this effect is more pronounced for N_2_ (Figure 5a). At the lowest two dosing concentrations and all three temperatures, N_2_ uptake in Cu_2.7_-MFU-4*l* equilibrated more rapidly than that in O_2_ (∼20 *versus* 50 s). This difference was minimized at the highest two dosing concentrations of 5.0 and 10.0 mmol/g (Figures 5b, S22, and S23), and the saturation times for both gases appeared to approach the diffusion-controlled time scales measured for Ar adsorption kinetics (∼20 s, see Figure S21).

The adsorption data collected following dosing with 1.0 mmol/g of O_2_ or N_2_ could be satisfactorily fit (*R*^2^ > 0.99) with a pseudo-first order rate law model using the Lagergren equation.^52^ Activation energies calculated using the Arrhenius equation are similar for O_2_ adsorption [*E*_a_ = 10(1) kJ/mol] and N_2_ adsorption [*E*_a_ = 12(1) kJ/mol] (Table S6 and Figure S26). Identical activation barriers were calculated using data collected under the most dilute dosing conditions (0.5 mmol/g; see Figure S27). Diffusion time constants calculated from the kinetics data (see the Supporting Information for details) indicate that diffusion of N_2_ is more rapid than O_2_, which we attribute to a weaker N_2_ binding within the framework (Table S7 and Figure S28).^53^ The relatively large diffusion time constants are indicative of rapid diffusion kinetics facilitated by large framework pores.

Following adsorption analysis, variable-temperature O_2_ and N_2_ desorption kinetics data were collected under reduced pressure (Figures 5c, S24, and S25), which revealed that O_2_ desorption from the material is more sluggish than N_2_ desorption. For example, after dosing with 1.0 mmol/g of each adsorbate at 298 K, half-life (*t*_1/2_) values extracted for O_2_ and N_2_ desorption were 161 and 16 s, respectively (Figure 5c), and this difference becomes even more pronounced upon lowering the temperature to 288 K (*t*_1/2_ values of 260 and 53 s, respectively; see Figures S24 and S25). The desorption curves for both gases were fit using a pseudo-first order rate law, and the resulting data were used to calculate activation barriers for O_2_ and N_2_ desorption of *E*_a_ = 45(1) and 30(1) kJ/mol, respectively (Figure S26 and Table S6). Importantly, although the kinetics of O_2_ and N_2_ adsorption in Cu_2.7_-MFU-4*l* are similar and both gases are expected to be adsorb under conditions relevant to air capture, these results suggest that it may be possible to tailor the desorption conditions to isolate high-purity O_2_.

### Breakthrough Analysis

**Figure 6:**
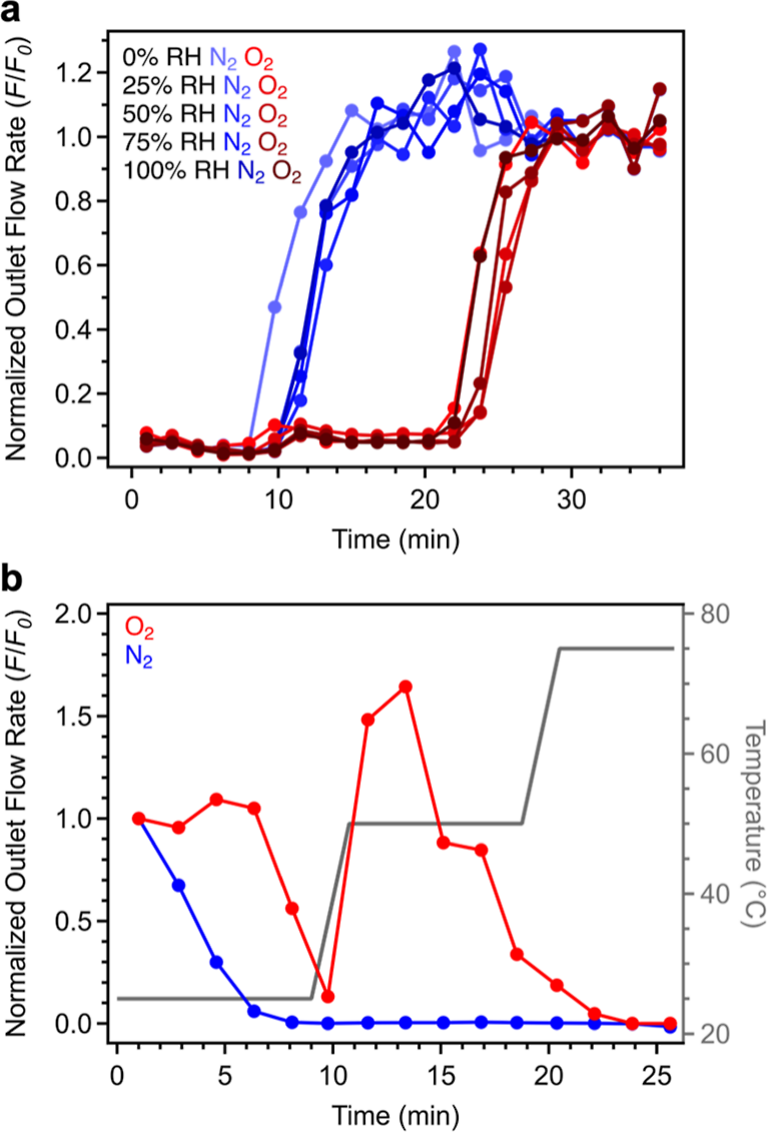
(a) Breakthrough profiles collected for Cu_2.7_-MFU-4*l* exposed to compressed air streams with the indicated humidity levels. Sharp breakthrough of N_2_ occurs before O_2_, highlighting the selectivity of the framework for O_2_ over N_2_. Symbols correspond to averages of data from triplicate runs, and solid lines are guides for the eye (see Figures S51–S55 for individual data sets and Table S12). Small quantities of O_2_ detected after 10 min before breakthrough are attributed to displacement of a small amount of O_2_ by N_2_. A small amount of both gases detected above the baseline at *t* = 0 is attributed to trace air present within the connection between the breakthrough column and the gas chromatograph that remains after flushing the system prior to the start of the measurement (see Section S1.13 of the Supporting Information and Figure S62 for details). (b) Oxygen and N_2_ desorption breakthrough profiles for Cu_2.7_-MFU-4*l*. Nitrogen was desorbed first by purging the material with He gas at 25 °C, and O_2_ was subsequently desorbed under He gas at 50 °C. The O_2_ and N_2_ concentrations were quantified using gas chromatography, which led to a relatively low signal-to-noise ratio.

Breakthrough measurements were conducted at 25 °C using pelletized Cu_2.7_-MFU-4*l* and compressed air inlet streams (2 mL/min) with varying levels of humidity to assess the selectivity of Cu_2.7_-MFU-4*l* for O_2_ and N_2_ under more realistic conditions (see Section S1.13 of the Supporting Information for details). We note that negligible uptake of CO_2_ in anticipated under these conditions based on single-component CO_2_ adsorption data collected for isotherm Cu_2.7_-MFU-4*l* at 25 °C (Figure S11). When the material was exposed to dry air, a sharp breakthrough of N_2_ occurred after 10 min, followed by breakthrough of O_2_ after 25 min. This result highlights the selective nature of the binding of O_2_ in Cu_2.7_-MFU-4*l* under these conditions (Figure 6a). Additionally, while not the focus of this work, the separation of the N_2_ and O_2_ breakthrough products under these conditions suggests that Cu_2.7_-MFU-4*l* may also be a viable adsorbent for purifying N_2_ from air, although we note that in this case pretreatment of the air, or post-treatment of the recovered N_2_, may be needed to separate other trace air contaminants, depending on the intended use and required N_2_ purity. Following breakthrough of N_2_ from the column, the normalized outlet flow rate (*F*/*F*_0_) for N_2_ temporarily exceeded the inlet flow rate, indicative of roll-up^54^ from displacement of bound N_2_ by O_2_ due to competitive adsorption at the same bind sites. After this initial breakthrough run, the material was regenerated with heating at 150 °C under a He purge (until O_2_, N_2_, and H_2_O were no longer detected in the outlet stream), completing the first breakthrough “cycle”. The material was then cooled to ambient temperature, and two more adsorption/desorption cycles were performed under the same conditions. The breakthrough times and capacities for O_2_ and N_2_ did not change over the course of these three cycles (Figures 6a and S51 and Table S12).

Using the same sample, three breakthrough cycles were subsequently carried out in succession, involving adsorption of compressed air streams with relative humidity levels of 25, 50, 75, and 100% and regeneration with heating at 150 °C under He (Figures S52–S55). There was no significant change in the measured breakthrough times for O_2_ over the course of the 15 adsorption runs (Figure 6a), and there was no apparent change in the corresponding material capacity (Figures S56 and S57 and Table S12). Indeed, the average O_2_ capacity from triplicate measurements under dry conditions was the same as that measured at the highest humidity (Figure S56 and Table S12), indicating that water neither hinders the material performance nor competes with O_2_ for binding at the copper(I) sites. Finally, after the third breakthrough run at 100% relative humidity, two additional breakthrough cycles at the same relative humidity were performed with more mild regeneration under flowing He at only 50 °C (Figure S55). Although some water remains adsorbed in the material following desorption under these conditions (Figure S60), there was no apparent change in the material capacity.

The O_2_ and N_2_ capacities determined from averaging over all 17 breakthrough runs are 1.2 and 1.7 mmol/g, respectively. These capacities differ slightly from those determined from single-component isotherm data, namely, 1.5 and 1.3 mmol/g, respectively. A lower O_2_ capacity from breakthrough analysis is consistent with competition between O_2_ and N_2_ binding in the material, highlighting the fact that single-component adsorption data may not accurately reflect the adsorption behavior of a MOF when exposed to a mixed-gas stream. Interestingly, the N_2_ capacities determined from the humid breakthrough data are overall higher than the single-component adsorption capacity. This phenomenon is currently not understood, and while the capacity values should be interpreted with caution in the absence of statistical errors, this result may indicate that the presence of humidity enhances N_2_ uptake in Cu_2.7_-MFU-4*l*. Importantly, the breakthrough data reveal that the framework retains selectivity for O_2_ over N_2_ when exposed to air streams with varying humidity levels, and also that there is an enhancement in the quantity of O_2_ in the adsorbed phase in Cu_2.7_-MFU-4*l* relative to ambient air.

Finally, we sought to exploit the differences in N_2_ and O_2_ desorption kinetics discussed above and identify conditions, under which it would be possible to separately isolate adsorbed N_2_ and O_2_. We found that it is indeed possible to desorb the majority of the bound N_2_ from the material at 25 °C under simulated vacuum with a He purge, after which point high-purity O_2_ can be isolated from the material (containing <0.5% N_2_ based on analysis of the stream composition using gas chromatography) by ramping the temperature to 50 °C (Figure 6b). Desorption of both N_2_ and O_2_ could also be conducted entirely at 25 °C, albeit with relatively sluggish kinetics for complete O_2_ desorption, indicating a trade-off between desorption rates and thermal input for regeneration (Figure S59).

## Conclusions

We have optimized the synthesis of the well-known metal–organic framework Cu^I^-MFU-4*l* and studied its O_2_ binding properties under various conditions relevant to O_2_ capture from air in the presence of water vapor. Spectroscopic, magnetic, and computational analyses revealed that the copper(I) sites bind to O_2_*via* electron transfer to form copper(II)–superoxo species. Interestingly, the superoxo moieties bind in both side- and end-on modes at the copper(II) sites, and these modes are in equilibrium over a range of temperatures. Breakthrough cycling experiments indicate the material is stable to extended cycling under dry and humid air streams and reversibly captures O_2_ from ambient air in the presence of water. While both O_2_ and N_2_ rapidly adsorb in the material under these conditions, the activation barrier for O_2_ desorption is higher than that for N_2_ desorption, and this feature can be exploited to access high-purity O_2_ (>99%) after initial N_2_ desorption. Breakthrough analyses further indicate that the O_2_ capacity of the material is unaffected by the presence of humidity, suggesting coadsorbed water does not bind to the exposed Cu^I^ sites. These results highlight the advantages of using soft copper(I) sites in MOFs for selective O_2_ capture in the presence of water. Further, while high adsorption selectivity for O_2_ over N_2_ has traditionally been sought in candidate MOFs for O_2_-selective air separations, our results reveal that differences in the desorption kinetics can be used to access high purity O_2_ even when adsorption behavior suggests relatively low selectivity for O_2_. This discovery suggests that it may be worthwhile to reinvestigate existing materials that have been overlooked based on preliminary analysis of their single-component O_2_ and N_2_ adsorption behavior. Research is ongoing in our laboratory to further advance Cu^I^-MFU-4*l* for practical air separations, including the scaleup of the materials developed here, the impact of trace air contaminants on long-term stability, and the study of hydrophobic polymer^55^ coatings to minimize water uptake.

## Supporting Information Available

Additional synthesis and characterization details, adsorption, kinetics, breakthrough, and computational data (PDF)
(CIF)

## Supplementary Material

ja3c10753_si_001.pdf

ja3c10753_si_002.cif
