# Supplementary material for: Selective Adsorption of Oxygen from Humid Air in a Metal–Organic Framework with Trigonal Pyramidal Copper(I) Sites
Source: J Am Chem Soc. 2024 Jan 26;146(5):3160–70. doi: 10.1021/jacs.3c10753 (PMC10859921; doi:10.1021/jacs.3c10753)
Supplement: Supplementary file 1 — ja3c10753_si_001.pdf [file ja3c10753_si_001.pdf]

Supporting Information for:

Selective Adsorption of Oxygen from Humid Air in a Metal–Organic Framework with Trigonal Pyramidal Copper(I) Sites

Kurtis M. Carsch,[1,2] Adrian J. Huang,[1,2] Matthew N. Dods,[1,3] Surya T. Parker,[1,3] Rachel C. Rohde,[1,2] Henry Z. H. Jiang,[1,2,4] Yuto Yabuuchi,[1,2,4] Sarah L. Karstens,[1,2] Hyunchul Kwon,[1,2] Romit Chakraborty,[1,2,4] Karen C. Bustillo,[5] Katie R. Meihaus,[1,2] Hiroyasu Furukawa,[1,2,4] Andrew M. Minor[5,6], Martin Head-Gordon,[1,2,4] Jeffrey R. Long[1,2,3,4]*

[1]Institute for Decarbonization Materials, University of California, Berkeley, Berkeley, California 94720, United States.

[2]Department of Chemistry, University of California, Berkeley, Berkeley, California 94720, United States.

[3]Department of Chemical and Biomolecular Engineering, University of California, Berkeley, Berkeley, California 94720, United States.

[4]Materials Sciences Division, Lawrence Berkeley National Laboratory, Berkeley, California 94720, United States.

[5]National Center for Electron Microscopy, Molecular Foundry, Lawrence Berkeley National Laboratory, Berkeley, California 94720, United States.

[6]Department of Materials Science and Engineering, University of California, Berkeley, Berkeley, CA, 94720, USA.

Table of Contents

1. Materials and Methods S3
2. Syntheses S12
3. Supporting Isothermal Characterization Data S16
4. Supporting Isobaric Characterization Data (Thermogravimetric Analysis) S27
5. Kinetic Measurements S31
6. Supporting Spectroscopic Characterization Data S36
7. Supporting X-ray Diffraction Characterization Data S44
8. Magnetic Characterization Data S51
9. Breakthrough Measurements. S52
10. Additional Characterization S59
11. Computational Details S63
12. References S74

1. Materials and Methods

1.1. General Considerations. All manipulations of evacuated porous materials were conducted either in the absence of water and dioxygen using standard Schlenk techniques or in an inert atmosphere glovebox under a N_2 or Ar atmosphere, unless specified otherwise. For air-free manipulations, glassware was oven-dried at 150 °C (> 2 h) and cooled in an evacuated antechamber for a minimum of 20 min prior to use in the dry box. Anhydrous dimethylsulfoxide was purchased from Sigma-Aldrich, sparged with Ar for a minimum of 45 min, then dried over 3 Å molecular sieves in a dry box before use for a minimum of 48 h. Other anhydrous solvents, including 1,2-difluorobenzene, were dried using a commercial solvent purification system designed by JC Meyer Solvent Systems and stored over 3 or 4 Å activated molecular sieves (Strem) prior to use. Anhydrous cupric chloride (99.999% purity, CAS 7447-39-4) was purchased from Sigma-Aldrich and used as received. Silver triflate (CAS 2923-28-6) was purchased from Sigma-Aldrich, used as received, and stored with exclusion of light. Lithium formate monohydrate (CAS 6108-23-2) was purchased from Sigma-Aldrich and used as received. The framework $Zn_5Cl_4(btdd)_3$ (MFU-4*l*; H_2btdd= bis(1*H*-1,2,3-triazolo[4,5-*b*],[4′,5′-*i*])dibenzo[1,4]dioxin) was synthesized following reference 1 and evacuated at 120 °C under dynamic vacuum for a minimum of 12 h before further use. The synthesis of $Cu_{2.2}$-MFU-4*l* was carried out using the route reported in reference 2. Elemental analyses (C, H, N) were performed by the Microanalytical Facility at the University of California, Berkeley using a Perkin-Elmer 2400 Series II combustion analyzer.

1.2. Ambient Temperature Gas Adsorption Measurements. Gas adsorption isotherms were measured by a manometric method using a Micromeritics ASAP 2020 gas sorption analyzer, a Micromeritics ASAP 2420 gas sorption analyzer, or a Micromeritics 3Flex gas sorption analyzer. A typical sample of approximately 100–200 mg of material was transferred to a Micromeritics ASAP analysis tube that was previously tared under vacuum. Prior to all gas sorption measurements, the analysis tube containing the partially activated material was capped with a Micromeritics TranSeal and evacuated on either a Micromeritics ASAP 2020 instrument or a Micromeritics Smart VacPrep instrument. The evacuated ASAP analysis tube containing the activated sample was carefully transferred to an electronic balance and massed to determine the mass of sample. The sample was subsequently transferred to an Ar-filled glovebox, in which the TranSeal was removed, and a glass rod of near-equal height was added to minimize headspace volume and provide a conduit for thermal transfer. The TranSeal was re-attached, and the tube was then transferred to the analysis port of the gas sorption analyzer. The outgas rate was subsequently confirmed to be less than 2 μbar/min following exposure of the sample to vacuum. For all isotherms, warm and cold free space correction measurements were performed using ultra-high purity He gas (UHP grade 5.0, 99.999% purity). Oxygen and N_2 isotherms were collected using UHP grade gas sources and were measured in water baths equipped with a Julabo F32 circulator. Water isotherms were collected using a Micromeritics 3Flex gas dosing analyzer. Water isotherms were collected out to only 80–90% relative humidity to prevent condensation of water within the instrument manifold. Prior to water isotherm measurements, a stainless-steel vapor dosing apparatus filled with Milli-Q water was subjected to four freeze–pump–thaw cycles to remove dissolved gases while attached to the gas dosing analyzer. The isotherm data points were considered equilibrated after a <0.010% change in pressure occurred over an average of 11 intervals (30 sec equilibration time). Oil-free vacuum pumps and oil-free pressure regulators were used for all measurements to prevent contamination of the samples during the evacuation process or of the feed gases during the isotherm measurements.

1.2.1. Low-pressure argon adsorption measurements. In an Ar-filled glovebox, pre-activated sample (~100 mg) was transferred to a stainless-steel sample holder equipped with a diaphragm-sealed valve, which was then evacuated at 100 °C for 1 h. Argon isotherms were collected between 0 and 1.1 bar on a Quantachrome Autosorb iQ2 physisorption analyzer. The analysis temperature (170, 180, and 190 K) was controlled using a cryostat (CryoCooler, Quantachrome) with cryogenic temperatures necessary to achieve appreciable uptake of the adsorbate. The void volume of the analyzer system was estimated using He. Ultra-high purity Ar and He gases (UHP grade 5.0, 99.999% purity) were used throughout the adsorption analyses.

1.3. Adsorption Isotherm Fitting. Single-component adsorption isotherms (O_2, N_2) were fit simultaneously for adsorption data points at temperatures 288, 298, and 308 K (Figure S5) using a dual-site Langmuir-Freundlich equation:

$$n = \frac{q_{sat,1} b_1 p^{v_1}}{1 + b_1 p^{v_1}} + \frac{q_{sat,2} b_2 p^{v_2}}{1 + b_2 p^{v_2}} \tag{1}$$

Here, n denotes the quantity adsorbed (mmol/g), $q_{sat,i}$ denotes the saturation capacity of a specific component, b denotes the Langmuir parameter (bar^{-1}), P denotes pressure (bar), v denotes the dimensionless Freundlich parameter, and subscripts i denote the strong (1) or weak (2) binding site. The appropriate parameters were determined through a simultaneous least-squares fitting (Microsoft Excel, version 16.68) of three isotherms with values provided in Table S4. The Langmuir parameter b_i can be further given by the following equation:

$$b = e^{\frac{S_i}{R}} e^{\frac{-1000 E_i}{RT}} \tag{2}$$

Here, S_i denotes the site-specific integral entropy of adsorption (J mol^{-1} K^{-1}), E_i denotes the site-specific differential enthalpy of adsorption (kJ mol^{-1}), R denotes the ideal gas constant (J mol^{-1} K^{-1}), and T denotes temperature (K).

1.4. Isosteric Enthalpy of Adsorption Calculations. The differential enthalpies of adsorption as a function of gas loading were extracted from simultaneous fits of three single-component dual-site isotherms at three distinct temperatures for O_2 and N_2 (288, 298, and 308 K; Figure S5). For Ar isotherms, the fitting was conducted using adsorption isotherms collected at 170, 180, and 190 K (Figure S6). The Clausius–Clapeyron relationship was subsequently employed at constant loadings to calculate the resulting enthalpy of adsorption:

$$\Delta H_{ads} = -RT^2 \left(\frac{\partial lnP}{\partial T}\right)_n \tag{3}$$

Here, ΔH_{ads} is the isosteric enthalpy of adsorption (kJ mol^{-1}), R is the ideal gas constant (J mol^{-1} K^{-1}), P denotes a pressure at a specific gas loading of n (bar^{-1}), and T denotes the temperature (K) of the single-component isotherm measurement. The isotherm fits were numerically inverted and solved as $P(n)$ in 0.05 mmol g^{-1} intervals (Microsoft Excel, version 16.68, solver function). At each loading, the slope of the best-fit line to ln(P) against T^{-1} was calculated to obtain the differential enthalpy of adsorption.

1.5. IAST Calculations. Ideal adsorbed solution theory (IAST)[3] calculation was used to determine adsorbent selectivity values from single-component gas adsorption isotherms (Figure S12).

Specifically, this approach entailed numerically solving for the spreading pressure and determining the component of the adsorbed phase at a specific gas composition. For a binary O_2/N_2 mixture, the adsorption selectivity was calculated from the following equation:

$$S = \frac{\frac{n_{O_2}}{n_{N_2}}}{\frac{x_{O_2}}{x_{N_2}}} \tag{4}$$

Here, *S* denotes the selectivity factor, *n* denotes the mole fraction in the adsorbed phase, *x* denotes the mole fraction in the gas phase, and the subscripts refer to O_2 or N_2 components. IAST calculations were further employed to assess the selectivity for binary O_2/Ar and N_2/Ar mixtures.

1.6. Thermogravimetric Analysis. Thermogravimetric analysis data were collected for $Cu_{2.7}$-MFU-4*l* using a TA Instruments Discovery TGA with samples loaded under air. Masses were not corrected for buoyancy effects. Thermogravimetric decomposition experiments were collected under N_2 with a temperature ramp rate of 1.0 °C/min. Before data collection, $Cu_{2.7}$-MFU-4*l* was activated at 100 °C (under Ar at a ramp rate of 10 °C/min) for a minimum of 30 min to remove volatile species. The sample was then cooled to 30 °C (under Ar, ramp rate of 20 °C/min) and allowed to thermally equilibrate for 10 min prior to data collection. For adsorption/desorption cycling studies, a looping procedure was employed to repeat a desired sequence for the specified time duration. See Figures S18 and S19 for details.

1.7. Inductively Coupled Plasma Optical Emission Spectroscopy (ICP-OES). ICP-OES measurements were performed on an Optima 7000 DV instrument maintained by the Microanalytical Laboratory at the University of California, Berkeley. In brief, framework samples (<1 mg) were digested in a mixture of sulfuric acid and dimethylsulfoxide (~1 mL) and then diluted in Milli-Q ultrapure water to a concentration of 1–10 ppm Zn, Li, and Cu. Measurements were initially calibrated with Zn, Li, and Cu standard solutions with concentrations between 0.1 and 10 ppm. All measurements were conducted in triplicate.

1.8. Infrared Spectroscopy and Diffuse Reflectance Infrared Spectroscopy (DRIFTS). Ambient temperature Fourier transform infrared (FTIR) spectra were recorded on a Perkin Elmer Avatar Spectrum 400 FTIR Spectrometer equipped with an attenuated total reflectance (ATR) attachment under air (<1 mg sample). DRIFTS data were collected using a Bruker Vertex 70 spectrometer equipped with a glowbar source, KBr beamsplitter, and a liquid N_2-cooled mercury-cadmium-telluride detector. A custom-built diffuse reflectance system with a static gas dosing cell equipped with ZnSe windows was used. Sample temperature was controlled by an Oxford Instruments OptistatDry TLEX cryostat, and sample atmosphere was controlled by a Micromeritics ASAP 2020 Plus gas sorption analyzer. The framework $Cu_{2.4}$-MFU-4*l* (10 wt%) was dispersed in dry diamond powder in an Ar-filled glovebox, loaded into the sample cell, and evacuated at room temperature for 30 min prior to data collection. Known pressures of UHP-grade natural abundance O_2 (>99.8% $^{16}O_2$) and $^{18}O_2$ (97 atom% ^{18}O, Sigma-Aldrich) were dosed into the sample using a Micromeritics ASAP 2020 Plus gas sorption analyzer. Spectra at 4 cm^{-1} resolution were generated from 128 scans collected over the course of approximately 35 seconds, and collected at 1-min intervals until no further changes were observed (typically 8–10 min). All spectra were processed in pseudo-absorbance units.[4] Difference spectra were generated by subtracting the spectrum of the activated framework from spectra of the framework dosed with O_2 (Figure S35) or with air (Figure S36), and isotopic difference spectra were generated by subtracting

the spectra of the framework dosed with $^{18}O_2$ from spectra of the framework dosed with O_2 at corresponding pressures and temperatures (Figure 3a,b).

1.9. Energy-Dispersive X-ray Spectroscopy. Under air, a sample of framework (typically 1–2 mg) was crushed with a spatula and dispersed in methanol (~1 mL). A droplet was transferred onto a gold grid covered with lacey carbon and dried in air. Data was collected using a Thermo Fisher Scientific TitanX microscope operated at 300 kV. The STEM convergence angle was 10 mrad and the annular dark field inner collection angle was ~45 mrad. STEM EDS data were acquired using a Bruker windowless EDS detector with a solid angle specified as 0.7 steradians. The EDS maps used a pixel size of 14 and 10 nm and the beam currents were 0.83 and 0.75 nA for $Cu_{2.7}$-MFU-4*l* and $Cu_{2.4}$-MFU-4*l*, respectively. EDS data were processed using Bruker ESPRIT software. Quantitative analysis was performed according to the Cliff-Lorimer approach;[5] background subtraction and peak integration were carried out before quantification. For all elemental maps and quantitative analysis for determining the ratios of Cu to Zn, K-edges were used. When determining the amount of Cl, the Cl K-edge was compared to the Cu and Zn L-edges.

1.10. Powder X-ray Diffraction. Standard diffraction patterns on samples of $Cu_{2.4}$-MFU-4*l* were collected with 0.01° steps using a Bruker AXS D8 Advance diffractometer equipped with Cu−Kα radiation (λ = 1.5418 Å), a Göbel mirror, a Lynxeye linear position-sensitive director, and the following optics: fixed divergence slit (0.6 mm), receiving slit (3 mm), and secondary beam Soller slits (2.5°). The generator was set at 40 kV and 40 mA. Samples were loaded on zero background sample holders. High-resolution powder x-ray diffraction patterns for $Cu_{2.4}$-MFU-4*l* were collected at Beamline 17-BM-B at the Advanced Photon Source (APS) at Argonne National Laboratory. The diffraction patterns were collected between 100 and 298 K with wavelengths of 0.45192 Å. Scattered intensity was recorded by a Varex 4343CT a-Si Flat Panel detector. Owing to the large number of data collected, all diffraction patterns were rebinned to a step size of 0.005° in 2θ.

For data collected at the APS, sample was loaded into a 1.0 mm borosilicate capillary, and the capillary was attached to a custom-designed valve gas-dosing cell and transferred to the goniometer head. The sample was heated under dynamic vacuum at 180 °C *in situ* to activate the material. Following cooling to 195 K, diffraction data were collected for the activated framework. Then, diffraction data were collected at 195 K as the framework was dosed with O_2 at pressures up to 8 mbar. The framework was then warmed up to 100 °C under vacuum to remove adsorbed O_2. The framework was then cooled to 195 K, and then N_2 gas was dosed until the equilibrium pressure was 9 mbar, after which diffraction data for N_2-dosed framework was collected. An Oxford Systems Cryostream 800 was used to change the temperature to room temperature. The wavelength was 0.45192 Å. Diffracted intensity was recorded by a PerkinElmer a-Si panel detector.

Unit cell parameters for activated and gas-dosed $Cu_{2.4}$-MFU-4*l* were obtained by structureless Pawley refinement using TOPAS-academic 6.1. The backgrounds of the pattern were modeled with Chebyshev polynomial functions. Peak shapes were described with the fundamental parameters approach. The solid-state structure of Cu^{I}-MFU-4*l* reported in reference 2 was used as the starting model of Rietveld refinement. The positions and atomic displacement parameters of the Cu atoms, the Zn atoms, the Cl atoms, and the atoms of the $btdd^{2-}$ ligand were refined with restraints akin to those provided in reference 6. Occupancies of the peripheral Cu atoms, the peripheral Zn atoms, and the capping Cl atoms were fixed based on the Cu/Zn ratio determined by the ICP-OES. When the Cl occupancy was freely refined, the number of chlorides was estimated

to be 1.91(6) per pentanuclear cluster, while the R_{wp} value showed little improvement (4.85% when the Cl occupancy was freely refined versus 4.91% when the Cl occupancy was fixed at 1.60 based on the Cu/Zn ratio from ICP-OES). For the refinement of the O_2-dosed framework, the refined structure of the activated framework was used as the starting model for Rietveld refinement, which revealed unmodeled electron density above Cu sites at Wyckoff position 96k, at which an oxygen atom was tentatively placed. Freely refining the occupancy of the oxygen atom gave the site occupancy of 35%, which can be translated to 117% occupancy of the O_2 molecule at the Cu^I site with a sizeable error bar (17%), precluding meaningful interpretation of the data. The large error bar and challenges in refinement are attributed to disorder about a special position and the cubic symmetry of the framework. The position and the atomic displacement parameters of the oxygen atom, peripheral Cu atom, the peripheral Zn atom, the peripheral Cl atom, and the atoms of the $btdd^{2-}$ linker were further refined with restraints. Hydrogen atoms were added after the refinement using the software Mercury[7] (linker C–H distances of 1.09 Å).

1.11. Kinetic Measurements. Kinetic studies were performed on a Micromeritics 3Flex sorption analyzer equipped with a temperature-controlled water bath. Glass rods were placed into the ASAP tubes to minimize headspace volume. The same sample of $Cu_{2.7}$-MFU-4*l* was used across all measurements for consistency.

Adsorption Measurements. For adsorption kinetics analysis, the framework was first activated at the analysis temperature under dynamic vacuum until the tube headspace pressure fell below 1.0 μbar. Then, N_2, O_2, or Ar was dosed into the tube at a dosing concentration of 0.5, 1, 5, or 10 mmol/g and temperatures of 288, 298, and 308 K. Tube headspace pressure was recorded over time at a sampling frequency of 2 Hz using the DataMonitor 3500 software package (Figure S20). From mass balance, gas uptake was calculated at each of the time points. At the end of each adsorption experiment, the gas-filled tube was closed from the manifold in preparation for desorption analysis. The data could be fit using the Lagergren equation:

$$\frac{\partial q_{ads}(t)}{\partial t} = k_{ads}[q_{sat} - q_{ads}(t)] \tag{5}$$

Here, q_{sat} is the saturation capacity for each measurement, and $q_{ads}(t)$ represents the capacity as a function of time. Activation barriers (E_a) were subsequently determined through the Arrhenius equation, by plotting $\ln(k_{ads})$ versus T^{-1} with the linear slope defined as $-E_aR^{-1}$. Data normalization was performed by plotting the data as a fraction of the capacity at time t relative to the capacity once equilibrated, selected as time t = 200 s.

Desorption Measurements. Desorption kinetic analyses were performed only for O_2 and N_2 since Ar adsorption at the examined temperatures (288, 298, and 308 K) is minimal. These analyses were conducted by reducing the manifold pressure to below 1 μbar and subsequently opening the tube to the evacuated manifold. Headspace pressure data were collected at a sampling frequency of 2 Hz until the pressure fell below 1 μbar (Figure S20).

Background Correction. For each of the initial headspace pressures before the gas-filled tube was opened to the evacuated manifold, a blank tube containing a glass rod without $Cu_{2.7}$-MFU-4*l* was analyzed at the identical temperature, and the same blank tube was used for all measurements. The blank tube was filled to approximately the same pressure as the sample, and the tube was subsequently isolated from the manifold. Then, the manifold pressure was reduced to below 1

μbar, and the blank tube was opened to the evacuated manifold. The headspace pressure of the blank tube was recorded over time at a frequency of 2 Hz until this pressure fell below 1 μbar. For each of the time points collected (excluding the first five points in each data set owing to pressure fluctuations), the blank pressure was subtracted from the headspace pressure. Before performing this subtraction, blank pressures were linearly adjusted such that the initial pressure was identical to that used for the sample-filled tube. This adjustment was performed due to slight mismatch between the initial pressures of the sample-filled tube and blank. Due to this adjustment, as well as to other factors (e.g., thermal fluctuations), the resulting desorption kinetic traces are qualitative.

Using the calculated pressure difference at each time point ΔP_t, gas uptake was computed over time with the following equation:

$$q_{des}(t) = q_{sat}[1 - \frac{\sum_{i=0}^{t} \Delta P_i}{\sum_{i=0}^{N} (\Delta P_i)}] \tag{6}$$

where q_{sat} is the saturation capacity for each temperature and dosing concentration, and the index N represents the total number of data points included in the desorption kinetic experiment. Because ΔP_t is initially zero, the desorption capacity is equal to the saturation capacity at $t = 0$. The rate constant k_{des} was subsequently calculated through application of the following equation (eq. 7):

$$\frac{\partial q_{des}(t)}{\partial t} = k_{des}[-q_{des}(t)] \tag{7}$$

Activation barriers (E_a) were subsequently determined through the Arrhenius equation, by plotting $\ln(k_{des})$ against T^{-1} with the linear slope defined as $-E_aR^{-1}$. Data normalization was performed by plotting the data as a fraction of the capacity at time t relative to the initial capacity following adsorption at time $t = 0$.

Diffusivity Measurements. Micropore diffusion to measure intracrystalline diffusivity was employed to calculate the diffuse time constant (D_c/r_c^2) from the following equation (eq. 8):

$$1 - \theta(t) = \frac{6}{\pi^2} \exp(-\pi^2 \frac{D_c}{r_c^2} t) \tag{8}$$

Here, $\theta(t)$ denotes the fractional occupancy between 70 and 99% reaction completion (determined from the normalized data plots, see Figure S28), which was observed to be highly prone to instrument error as the reaction approached completion. We note deviations from linearity suggest strong interactions of the adsorbates with the framework, reflecting engagement of the adsorbates with the exposed copper(I) site. Consequently, such analysis was applied only for dilute dosing conditions. Detailed information on micropore diffusion is provided in reference 8. As the adsorption of N_2 and O_2 to the framework reflect contributions of both chemisorption and physisorption, we present these data qualitatively to indicate that diffusion of N_2 proceeds more rapidly than diffusion of O_2 for adsorption in $Cu_{2.7}$-MFU-4*l*.

1.12. Dc Magnetic Susceptibility Measurements. Dc magnetic susceptibility data were collected under a field of 1 T at temperatures ranging from 2 to 300 K using a Quantum Design MPMS2 SQUID magnetometer. In brief, O_2-dosed $Cu_{2.4}$-MFU-4*l* was prepared by adding activated,

crystalline framework (29.6 mg) to a 5 mm i.d./7 mm o.d. quartz tube with a raised quartz platform. A layer of glass wool was compressed on top of the sample (4.4 mg) to minimize crystallite torquing. The sample tube was fitted with a Teflon sealable adapter, evacuated using a glovebox vacuum pump, and removed from the glove box. The sample tube was connected to a Schlenk line, evacuated for about 10 min at the minimum vacuum achievable on the manifold, and then dosed with O_2 (1 bar) at −78 °C with aid of a dry ice/acetone bath. The color of the solid immediately changed from off-white to pink. After standing for 10 min, the sample was again exposed to vacuum briefly (~10 min) at −78 °C to remove excess O_2 gas in the headspace of the tube. The color of the solid did not change noticeably during this manipulation. The portion of the tube containing the sample was then cooled in liquid nitrogen (77 K), and the tube was flame sealed with an O_2/H_2 flame under static vacuum. All data were corrected for diamagnetic contributions from the core diamagnetism of the sample and for the diamagnetism of the glass wool, estimated using Pascal's constants to give corrections of $\chi_{dia} = -0.0003908$ emu/mol for the framework and −0.0000222 emu/mol for glass wool. Over a portion of the measured temperature range, plots of the molar magnetic susceptibility–temperature product ($\chi_M T$) versus T (Figure S49) and χ_M versus T (Figure S50) drop below zero, indicating an error in the diamagnetic correction for the sample. As such, the measured susceptibility values cannot be interpreted as absolute, and are instead presented to highlight the overall change with changing temperature, which is indicative of a temperature-dependent spin state change, as discussed in the main text.

1.13. Breakthrough Measurements. For all breakthrough measurements, pellets of $Cu_{2.7}$-MFU-4*l* were prepared through mechanical compression. In brief, in an Ar-filled dry box, evacuated $Cu_{2.7}$-MFU-4*l* (0.72 g) was dampened with 1,2-difluorobenzene (~2-3 mL) and placed into a stainless-steel cylinder between a highly polished facet of a stainless-steel platform and a highly polished face of a stainless-steel plunger, as described in Figure S50 in reference 9. The powder was manually compressed between the platform and plunger, yielding a light beige, fragile tablet. This tablet was then manually compressed with a metal spatula against one sieve to yield pellets of 350–700 μm diameter (25–845 mesh). Finer particles that passed through the sieves were then taken through the same series steps (dampening with solvent, mechanical compression, and sieving) to isolate additional pellets. The pellets were then carefully transferred to an ASAP analytic tube equipped with a TranSeal and heated under dynamic vacuum on a Smart VacPrep at 250 °C (from room temperature, ramp rate of 1.0 °C/min) over the span of 4 d, yielding activated pellets of $Cu_{2.7}$-MFU-4*l* (~0.45 g). The resulting activated pellets were transferred into the Ar dry box and loaded into a breakthrough column (0.5 ft, 0.25-in stainless steel tubing, ~0.34 g of MOF).

Breakthrough experiments were conducted in-house using a custom-made apparatus (see Figure S62), composed of 1/8″ copper and stainless-steel tubing fitted with Swagelok fittings and mass flow controllers. The breakthrough column containing $Cu_{2.7}$-MFU-4*l* was connected to the breakthrough apparatus through Swagelok fittings and manually fixed with quartz wool. Heat tape and an internal thermocouple were used to control the temperature of the breakthrough column. A gas chromatograph (GC) was used to measure the outlet gas composition (either for O_2 and N_2 or for N_2 and Ar), and after the GC a flowmeter was attached to measure flow rate. The amount of water in the humid outlet gas streams from the humid O_2/N_2 breakthrough experiments was quantified using a nondispersive infrared sensor with a rapid sampling rate of once per second. Adsorption breakthrough experiments were conducted using compressed air streams without water and with humidity levels ranging from 25 to 100%. Humid streams were obtained by premixing a stream of compressed air that was fully saturated with water with the corresponding dry stream in the appropriate ratio (the case of 100% RH experiments, only the humid stream was used). For

instance, an air stream with 25% relative humidity was obtained by mixing a 1:3 ratio of a humid air to dry air, as controlled by two inlet mass flow controllers. The mixed stream was equilibrated at 15 sccm for 15 min and then 2 sccm for 10 min by allowing it to flow for through a series of tubes in the breakthrough setup, including the tubing connecting the breakthrough column to the GC (the column was closed off to the circulating gas during each equilibration period). This pre-equilibration step was employed for all O_2/N_2 streams and breakthrough measurements (including gas streams that did not require mixing, namely the dry compressed air and 100% humid compressed air streams). For N_2/Ar breakthrough measurements, a 1:1 gas mixture was prepared by premixing N_2 and Ar streams from separate cylinders. This mixed stream was also equilibrated at 15 sccm for 15 min and then 2 sccm for 10 min by allowing it to flow for through a series of tubes in the breakthrough setup (the column was closed off to the circulating gas during this equilibration period). Regeneration cycles involved purging the column with helium gas at 150 or 50 °C as discussed in the main text. Capacities reported in Figures S56–S57 and Table S12 were determined by integrating the area under a curve on a pre-calibrated GC.

We note that the flowmeter used in these experiments is calibrated for a minimum flow rate of 5 sccm, whereas we conducted these experiments using a lower flow rate of 2 sccm. This low flow rate was used given the small sample quantity used, which was limited by the volume of the breakthrough column. As a result of this lower flow rate, there is substantial noise in the breakthrough data for all gases.

In the O_2/N_2 breakthrough curves, a small amount of O_2 is recorded after 10 min, before O_2 breakthrough. We attribute this to slight displacement of bound O_2 by N_2. Initial flow detected in all breakthrough curves at $t = 0$ is attributed to the presence of residual air (or the N_2/Ar stream in the case of the N_2/Ar breakthrough data) in the dead space connection before the gas chromatograph (see labels in Figure S62), noted above. Ultimately, the selectivity of $Cu_{2.7}$-MFU-4*l* for O_2 over N_2, which is seen even in the presence of relative humidity, is reliably and consistently measured, and this is the key result of the breakthrough measurements.

1.14. Nuclear Magnetic Resonance. Solution-phase ^{1}H NMR spectra were collected on a Bruker AV-400 spectrometer at ambient temperature with chemical shifts referenced to residual dimethylsulfoxide (DMSO). Framework dissolution was achieved upon addition of either DCl (35 wt% D_2O) or D_2SO_4 (1–2 drops) to a suspension of the framework (~1–2 mg) in DMSO-d_6 (~0.7 mL), followed by sonication and briefly heating to 60 °C until apparent homogenization.

1.15. Gravimetric Cu(I) Loading Calculation. Activated samples of $Cu_{2.4}$-MFU-4*l* (0.1234 g) and $Cu_{2.7}$-MFU-4*l* (0.1762 g) in evacuated ASAP analysis tubes were tared in triplicate on an electronic balance. The samples were then individually attached to a Micromeritics 3Flex gas sorption analyzer. Once the manifold of the instrument was evacuated (<2 μbar), the TranSeal of the ASAP analysis tube was open, resulting in a pressure increase of <2 μbar and confirming retention of vacuum. The sample was then dosed with 50 mbar CO at 298 K and allowed to equilibrate at this pressure over the span of 10 minutes (the saturation of Cu^I sites with CO occurs at very low pressures <1 mbar at 298 K).[10] The sample was then evacuated to <0.1 μbar over 12 h to removed physisorbed CO. The TranSeal was closed, and then the sample was re-massed in triplicate on an electronic balance. The change in wt% corresponds to CO chemisorbed to exposed Cu^I sites.

1.16. Benchtop Humid Air Dosing. A gas washing bottle was partially filled with deionized water, and then a fritted bubbler was inserted into the bottle. Compressed air was pushed through

this manifold for 20 minutes to ensure saturation of the exhaust air stream with water. The outlet port of the washing bottle was connected using a needle to an ASAP analysis tube capped with a rubber septum containing $Cu_{2.7}$-MFU-4*l* under He (the ASAP analysis tube was previously silanized to prevent condensation of water on the sides of the tube). An exhaust needle was also placed into the rubber septum of the ASAP tube, which was then wrapped with aluminum foil and placed in a sand bath held at 25 °C. After sparging the ASAP analysis tube with humidified air for 30 minutes (corresponding to the average time for a single breakthrough measurement), the punctured septum was then replaced with a TranSeal. The aluminum foil was removed, revealing an expected color change from off-white to gray-brown and indicative of O_2 coordination to the framework. The sample was attached to a Micromeritics Smart VacPrep instrument and evacuated at 25 °C for 30 minutes, during which time a color change to off-white was observed. The sample was subsequently heated to 150 °C (1 °C/min) under dynamic vacuum, accompanied by an additional off-gassing event indicative of desorption H_2O. The sample was then subject to isotherm analysis (see Figure S10).

2. Syntheses

CuCl(SMe_2). CuCl(SMe_2) (DMS = dimethylsulfide) was synthesized by slightly adapting a literature procedure.[11] Under N_2, a 1 L Schlenk flask was charged with CuCl (10.0 g, 0.10 mol; 99.999% purity from Sigma-Aldrich), following by addition of excess anhydrous DMS (100 mL) via cannula transfer at room temperature. The mixture was briefly sonicated (~10 min) and the solids dissolved, yielding a faint yellow solution. In a well-ventilated fume hood, the sealed flask was open to air, and the mixture was filtered through a coarse porosity frit. Addition of anhydrous hexanes (100 mL) to the filtrate under air resulted in rapid precipitation of a highly crystalline colorless solid. After rapid stirring for ~5 min, the precipitate was collected on a filter frit and rinsed with ample hexanes (3 × 100 mL). All manipulations were conducted rapidly within 1 h of air exposure to prevent Cu^I oxidation, which is evident by discoloration from white to green. The resulting colorless, crystalline CuCl(SMe_2) (13.9 g, 86%) was collected in storage vessel, evacuated at room temperature under dynamic vacuum for 2 h, and stored under N_2 with exclusion of light at −35 °C until further manipulations. **Note**, this procedure can also be carried out using lower purity CuCl that is visibly contaminated with trace green copper(II) impurities. In this case, after stirring the suspension formed from CuCl in DMS under N_2 at 35 °C for 24 h and then cooling to room temperature, oxidized impurities can be readily removed by filtration through a coarse porosity frit, yielding CuCl(SMe_2) as a colorless, crystalline solid following precipitation with hexanes. Anal. Calc. for C_2H_6ClCuS (%): C 14.91, H 3.75, N 0.00, S 19.90; Found (%): C 14.87, H 3.72, N 0.00, S 19.83.

Synthesis of $Cu_{2.7}Zn_{2.3}H_{0.9}Cl_{0.7}$(btdd)$_3$ ($Cu_{2.7}$-MFU-4*l*)

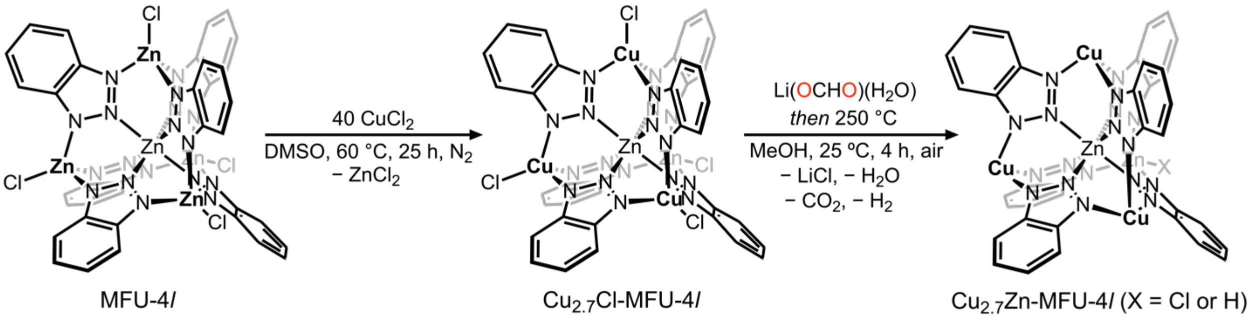

The following protocol was adapted from literature procedures.[2,10] In an inert atmosphere glove box, anhydrous $CuCl_2$ (0.530 g, 3.94 mmol, 40.0 equiv) was dissolved in anhydrous DMSO (20 mL) at 60 °C with stirring, and over the course of ~30 min, a deep green solution formed. The homogenous solution was transferred to a vial of activated MFU-4*l* (0.125 g, 0.0990 mmol, 1.00 equiv) and allowed to stand at 60 °C for 25 h. After cooling to room temperature, the vial was removed from the glove box, and the deep green solution was decanted from a lime green powder under air. The solids were transferred to a 50 mL Eppendorf tube as a suspension in DMSO and following centrifugation (4000 rpm, 5 min), the supernatant was decanted, and the solids were kept in the Eppendorf tube and re-suspended in fresh DMSO (40 mL) at room temperature for a minimum of 4 h; the DMSO was then decanted and replaced with 40 mL of fresh DMSO, and this mixture was again left to stand at room temperature for a minimum of 4 h. The DMSO was then decanted and replaced with methanol (20 mL) and this mixture was allowed to stand at room temperature for a minimum of 4 h, before the methanol was decanted. This process was repeated

with methanol five more times to yield a yellow-green solid. To isolate Cu^{II}-MFU-4*l*, the yellow-green solid was isolated by filtration and then heated to 180 °C under dynamic vacuum (1.0 °C/min ramp rate), resulting in a color change to brick-red, corresponding to activated Cu^{II}-MFU-4*l* ($Cu_{2.7}Zn_{2.3}Cl_4(btdd)_3$ based on ICP-OES to determine the Cu:Zn ratio).

To subsequently synthesize Cu^{I}-MFU-4*l*, the yellow-green solid isolated after the final methanol soak was combined with lithium formate monohydrate (0.300 g, 4.29 mmol, 43 equiv) dissolved in methanol (40 mL), and after agitation, a rapid color change from yellow-green to pale green. After standing for 2 h, the supernatant was decanted and a second solution of lithium formate monohydrate (0.300 g, 4.29 mmol, 43 equiv) dissolved in methanol (40 mL) was added. The mixture was agitated and allowed to stand for an additional 2 h. Following decanting of the supernatant, the solids were subsequently soaked seven times in methanol (30 mL) over a span of 48 h, allowing the solids to stand for a minimum of 2 h between solvent exchange. The final pale green solid isolated after the final soak was isolated by vacuum filtration and then transferred to an ASAP analysis tube and capped with a TranSeal. Following evacuation (2 h) at 70 °C under dynamic vacuum, the solids were evacuated for a minimum of 12 h at 95 °C on a Micromeritics ASAP 2020 instrument, after which time they had changed color to dark yellow. ICP-OES analysis of the resulting solid revealed the absence of Li. Solution-phase ^{1}H NMR spectroscopy analysis of an acid-digested sample of framework was used to estimate the number of formates per node ($Cu_{2.7}Zn_{2.3}(OOCH)_{3.3}Cl_{0.7}(btdd)_3$) (Figure S33).

Further thermolysis was carried out to generate Cu^{I}-MFU-4*l*, as follows. We note that this sequence of activation procedures is necessary to achieve the H_2 capacity shown in Figure S7, and deviations from this procedure result in lower H_2 capacity values in our hands. In an ASAP analysis tube, the sample was heated to 100 °C (0.1 °C/min ramp rate) and held at that temperature for 12 h, at which point the color turned to brown-yellow. The temperature was then ramped to 105 °C (0.1 °C/min ramp rate) and held there for 12 h, after which time the solid was observed to be even darker in color. The temperature was then ramped to 120 °C (0.1 °C/min ramp rate) and held there for 6 h, after which time the solid had changed color to off-white. This solid was heated to 180 °C (0.2 °C/min ramp rate) and held at that temperature for 2 h hold. During that time, the solid gradually changed color to light pink before changing color again to off-white. Finally, the solid was heated to 250 °C (1.0 °C/min ramp rate) and held there for 2 h, after which time no further color change was observed. The sample was cooled to ambient temperature, and resulting solid was isolated (0.099 g, ~87% yield). Analysis of the solid using a multitude of techniques (see section 2.1) revealed it to have the chemical formula $Cu_{2.7}Zn_{2.3}H_{0.9}Cl_{0.7}(btdd)_3$ ($Cu_{2.7}$-MFU-4*l*). Solution-phase ^{1}H NMR spectroscopy analysis following digestion of the material in DCl/DMSO-d_6 revealed no evidence for formic acid (Figure S34). The hydride ligands result from decarboxylation of formate at high temperatures, which has previously been demonstrated for the material $Zn_5H_x(Cl/OOCH)_{4-x}(btdd)_3$.[10] The material was stored under inert atmosphere until further manipulations. Exposure of $Cu_{2.7}$-MFU-4*l* to ambient air affords a rapid and reversible color change from off-white to gray-brown. Anal. Calc. for $C_{36}H_{12.9}Cl_{0.7}Cu_{2.7}N_{18}O_6Zn_{2.3}$ (%): C 37.92, H 1.14, N 22.11; Found (%): C 37.95, H 1.17, N 21.85.

The synthesis of $Cu_{2.7}$-MFU-4*l* can also be conducted entirely in air without the use of anhydrous DMSO under N_2 for Cu^{II} exchange. All solvent exchange procedures, reagent quantities, and activation procedures otherwise identical. This alternative route affords the desired framework with approx. 90% exposed Cu^{I} sites relative to the procedure as described above that employs anhydrous DMSO for the Cu^{II} exchange. This change in capacity is based on 77 K H_2 isotherm

data and a measured uptake of 1.94 mmol/g at 1 mbar (Figure S7). The decreased 1 mbar H_2 capacity is attributed to a lower extent of initial Cu^{II} incorporation in the framework as validated through ICP-OES analysis (~2.5–2.6 Cu ions per node instead of ~2.7–2.8 Cu ions per node).

Synthesis of $Cu_{2.4}Zn_{2.6}Cl_{1.6}(btdd)_3$ ($Cu_{2.4}$-MFU-4*l*)

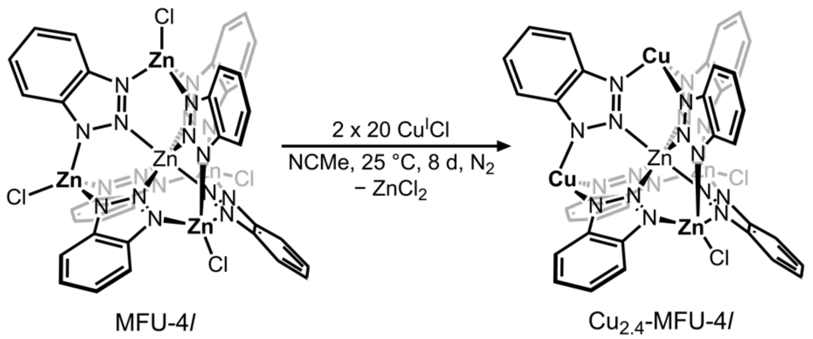

The synthesis was adapted from a literature procedure.[12] Under N_2, solid $CuCl(SMe_2)$ (0.200 g, 1.26 mmol, 20.0 equiv) was added to a suspension of MFU-4*l* (0.080 g, 0.063 mmol, 1.0 equiv.) in acetonitrile (20 mL). No color change was observed from the initial faint beige. The mixture was allowed to stand at room temperature for 4 d, followed by exchange of the supernatant with an identical solution of $CuCl(SMe_2)$ in acetonitrile (20 mL). After an additional 4 d, the microcrystalline powder was collected on a fine porosity frit and suspended in acetonitrile (~10 mL). This suspension was allowed to stand for 2 h and then the supernatant was decanted and replaced with fresh acetonitrile (20 mL). This process was repeated five more times, for a total of six soaks over two days. The solid was then suspended in methanol (~20 mL) for 12 h, and the solvent was decanted, and this process repeated. The solid was then suspended in benzene (~20 mL) and allowed to stand for 2 h. After the benzene was decanted, the solid was loaded into an ASAP analysis tube equipped with a TranSeal. Following a brief evacuation (2 h) at 80 °C under dynamic vacuum, the solids were evacuated for a minimum of 12 h at 80 °C on a Micromeritics ASAP 2020 instrument and then further evacuated at 300 °C (0.5 °C/min ramp rate) for a minimum of 2 h, affording $Cu_{2.4}Zn_{2.6}Cl_{1.6}(btdd)_3$ ($Cu_{2.4}$-MFU-4*l*) as a light beige solid (0.059 g, 79%). The material was stored under inert atmosphere prior to further manipulations. Exposure of $Cu_{2.4}$-MFU-4*l* to air affords a rapid and reversible color change from light beige to gray-brown. Anal. Calc. for $C_{36}H_{12}Cl_{1.6}Cu_{2.6}N_{18}O_6Zn_{2.4}$ (%): C 36.91, H 1.03, N 21.52; Found (%): C 37.07, H 1.03, N 21.79.

We note that the synthesis of $Cu_{2.4}$-MFU-4*l* can also be scaled up to 1 g of MFU-4*l* with all reagents and solvent quantities scaled proportionally.

2.1. Detailed Discussion on the Stoichiometry Characterization of $Cu_{2.7}$-MFU-4*l*

A multitude of techniques were used in determining the composition of $Cu_{2.7}$-MFU-4*l*, which features both a mixture of Cu^{I} and Cu^{II} ions as well as anion disorder at the Zn sites.

Thermogravimetric Analysis. Thermogravimetric analysis of $Cu_{2.7}$(OOCH)-MFU-4*l* with a thermal ramp under N_2 between 120 and 180 °C was used to estimate the quantity of Cu–OOCH sites based on mass loss of H_2 and CO_2, and a subsequent thermal ramp between 180 and 250 °C was used to estimate the quantity of Zn(II)–OOCH sites based on mass loss of CO_2 (see Figure S13), with the sum of these two values giving an estimate of the total weight percent of formate for the copper(II) precursor framework. Note that buoyance effects must be addressed for an accurate mass determination. We observed an ~11.4 wt% decrease in mass upon heating the activated material from 100 to 250 °C, indicating a stoichiometry of approximately 3.3 formate anions per cluster.

^{1}H NMR Spectroscopy. Proton NMR spectroscopy analysis of a digested sample of $Cu_{2.7}$(OOCH)-MFU-4*l* revealed resonances for the H_2btdd linker and formic acid, and integration of these was used to determine the extent of formate exchange (Figure S33; ~80–85% over multiple independent batches of material). Additionally, ^{1}H NMR spectroscopy analysis of an acid-digested sample of $Cu_{2.7}$-MFU-4*l* (Figure S34) featured no peak for formic acid.

Inductively Coupled Plasma Optical Emission spectroscopy (ICP-OES). ICP-OES was used to determine the Cu:Zn ratio for $Cu_{2.7}$(OOCH)-MFU-4*l* and for $Cu_{2.7}$-MFU-4*l*. Over multiple independent batches, we observed a Cu:Zn ratio ranging from 2.7:2.3 to 2.8:2.2. No Li could be detected. The conversion of $Cu_{2.7}$(OOCH)-MFU-4*l* to $Cu_{2.7}$-MFU-4*l* does not change the Cu:Zn ratio, as validated by independent ICP-OES measurements on both $Cu_{2.7}$(OOCH)-MFU-4*l* and $Cu_{2.7}$-MFU-4*l*.

Energy Dispersive Spectroscopy (EDS). EDS allowed for quantification of the relative ratios of Cu, Zn, and Cl, and the results are consistent with ICP-OES analysis (see Figure S29). Examination of multiple independent particles of $Cu_{2.7}$-MFU-4*l*, revealed a Cu:Zn ratio close to that of ICP-OES with ~0.7 Cl anions per cluster (measured ~0.8–0.9).

Isothermal H_2 Adsorption Measurements. As discussed in the main text, we used the H_2 uptake at 1 mbar and 77 K to estimate the number of Cu^{I} sites in $Cu_{2.7}$-MFU-4*l* (Figure S7).

Elemental Analysis. The elemental analysis (C, H, N) measured for $Cu_{2.7}$-MFU-4*l* is consistent with the anticipated chemical formula. Anal. Calc. for $C_{36}H_{12.9}Cl_{0.7}Cu_{2.7}N_{18}O_6Zn_{2.3}$ (%): C 37.92, H 1.14 N 22.11; Found (%): C 37.95, H 1.17, N 21.85.

Qualitative Detection of Chloride. Upon acidification and homogenization of the framework (~10 mg) in H_2SO_4 (~1 drop) and DMSO (~1 mL), addition of AgOTf (10 mg) in DMSO (~1 mL) resulted in immediate formation of a turbid solution, suggestive of residual Cl present in the framework due to formation of AgCl. Control experiments in the absence of the framework revealed no immediate precipitation of solids.

The combination of the above characterization techniques allows for an approximate formula of $(Cu^{II}(OOCH))_{2.4}(Cu^{II}Cl)_{0.3}(Zn(OOCH))_{0.9}(ZnCl)_{0.4}Zn(btdd)_3$ for $Cu_{2.7}$(OOCH)-MFU-4*l*. Notably, following thermolysis at 250 °C gives a formula of $(Cu^{I})_{2.4}(Cu^{II}Cl)_{0.3}(ZnH)_{0.9}(ZnCl)_{0.4}Zn(btdd)_3$ for $Cu_{2.7}$-MFU-4*l*.

3. Supporting Isothermal Characterization Data

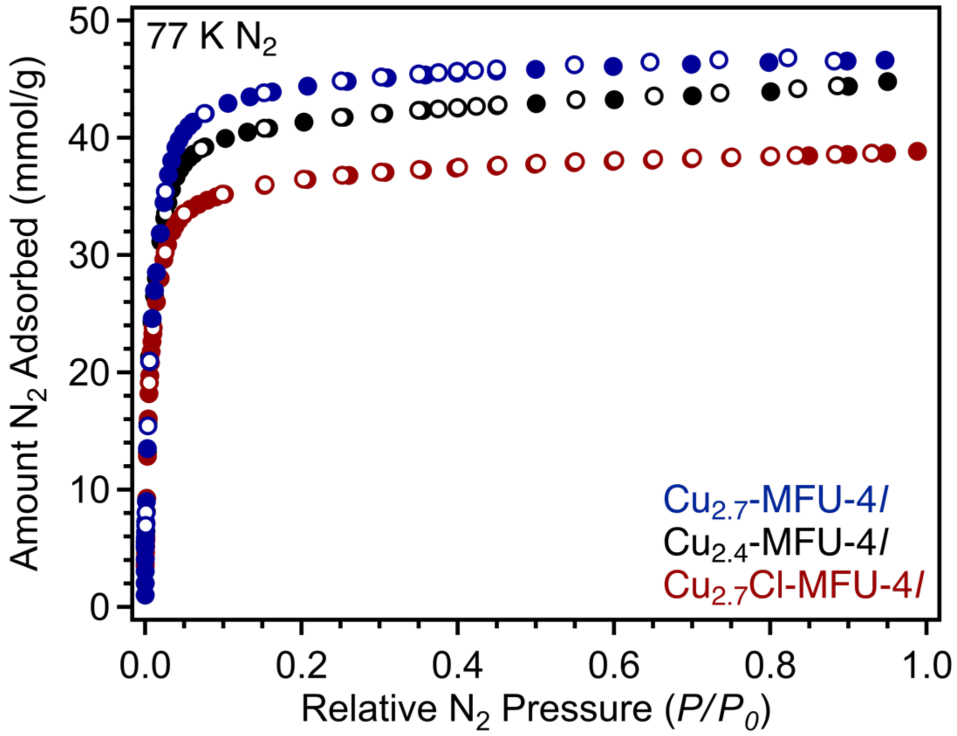

Figure S1. Nitrogen adsorption (filled circles) and desorption (open circles) isotherms measured at 77 K for $Cu_{2.7}$Cl-MFU-4*l* (red), $Cu_{2.4}$-MFU-4*l* (black), and $Cu_{2.7}$-MFU-4*l* (blue).

Table S1. Tabulated surface area values for MFU-4*l* series for newly reported syntheses.

Framework	BET Surface Area	Langmuir Surface Area
$Cu_{2.7}$Cl-MFU-4*l*	3340(30) m^2/g	3680(10) m^2/g
$Cu_{2.7}$-MFU-4*l*	4160(40) m^2/g	4500(20) m^2/g
$Cu_{2.4}$-MFU-4*l*	3820(30) m^2/g	4190(10) m^2/g

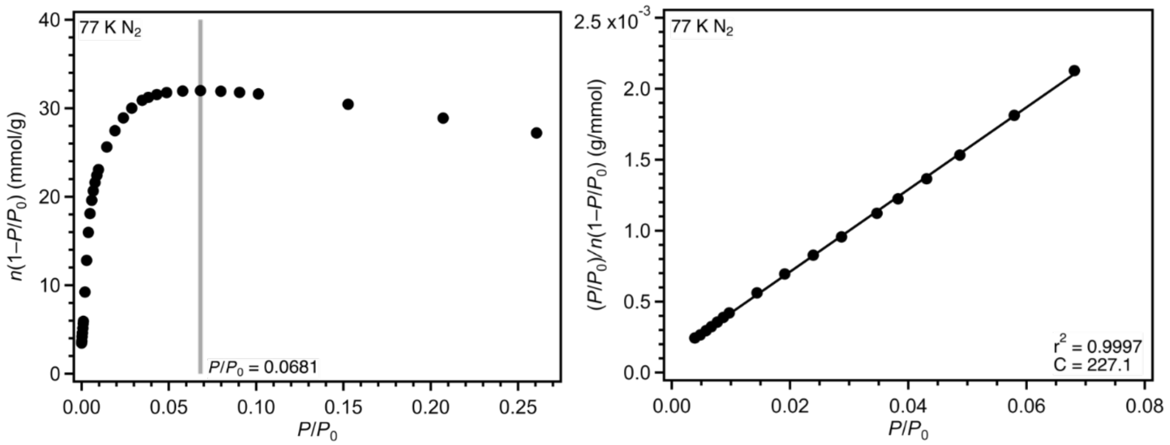

Figure S2. (Left) Plot of $n(1–P/P_0)$ versus P/P_0 for $Cu_{2.7}$Cl-MFU-4*l*, revealing only $P/P_0 < 0.0681$ satisfies the first consistency criterion for applying BET theory. (Right) Plot of $(P/P_0)/n(1–P/P_0)$ versus P/P_0 for $Cu_{2.7}$Cl-MFU-4*l*, revealing the linear regime for application of the BET equation.

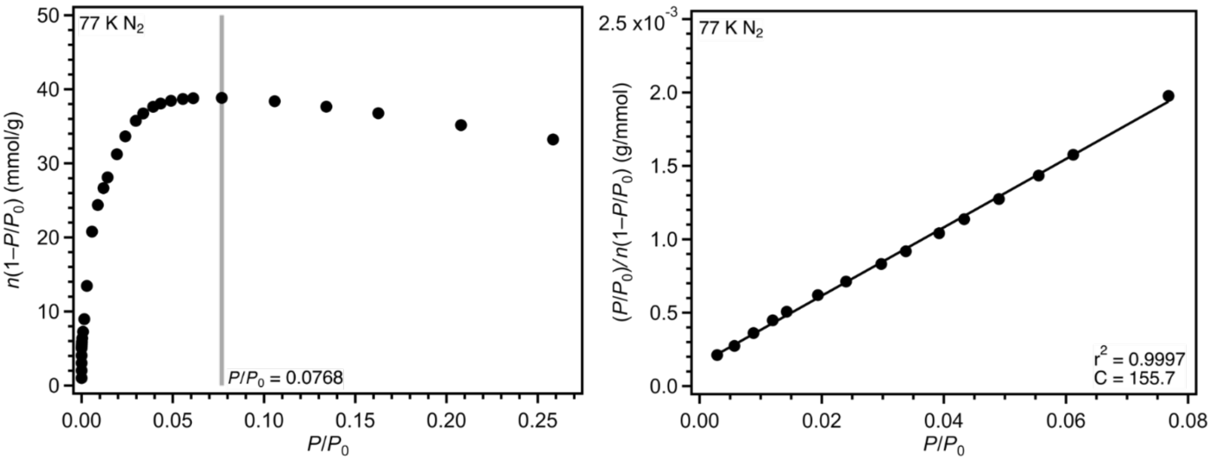

Figure S3. (Left) Plot of $n(1–P/P_0)$ versus P/P_0 for $Cu_{2.7}$-MFU-4*l*, revealing only $P/P_0 < 0.0768$ satisfies the first consistency criterion for applying BET theory. (Right) Plot of $(P/P_0)/n(1–P/P_0)$ versus P/P_0 for $Cu_{2.7}$-MFU-4*l*, revealing the linear regime for application of the BET equation.

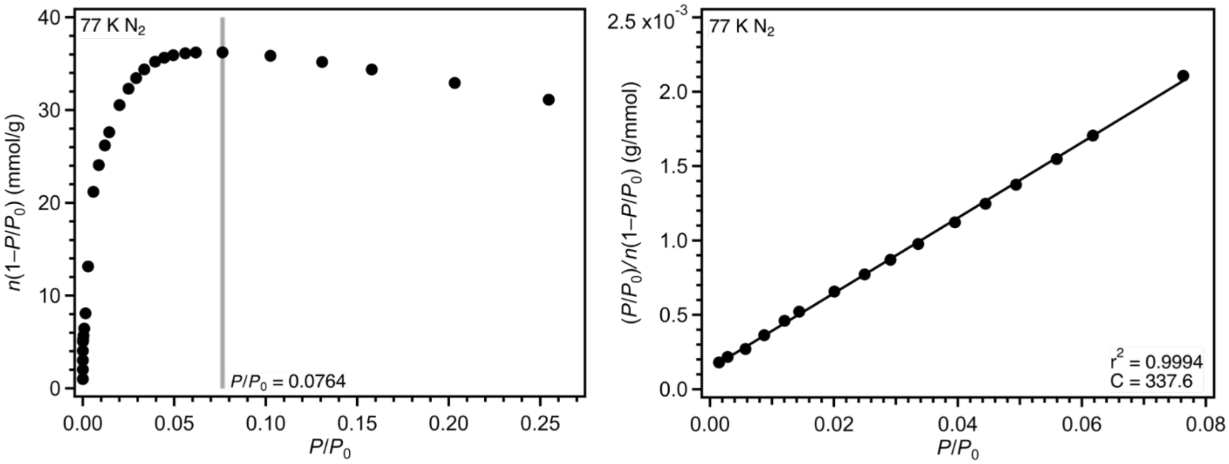

Figure S4. (Left) Plot of $n(1–P/P_0)$ versus P/P_0 for $Cu_{2.4}$-MFU-4*l*, revealing only $P/P_0 < 0.0764$ satisfies the first consistency criterion for applying BET theory. (Right) Plot of $(P/P_0)/n(1–P/P_0)$ versus P/P_0 for $Cu_{2.4}$-MFU-4*l*, revealing the linear regime for application of the BET equation.

Table S2. Brunauer–Emmett–Teller (BET) surface areas determined from N_2 (or Ar) adsorption data collected at 77 K (or 87 K) for Cu^I-MFU-4*l* samples prepared in the literature, along with reported copper loadings (based on ICP-OES) and copper(I) loadings (based on gas uptake attributed to binding at the copper(I) sites at a given pressure and temperature). These data highlight both the variability in the quality of Cu^I-MFU-4*l* samples prepared via various routes and the diverse methods used to report copper(I) loadings.

BET Surface Area	Reported Total Cu Loading	Loading at Cu^I Sites (based on gas uptake at given *P* and *T*) [a]	Reference
347 m^2/g (N_2)	3.2 Cu	0.4 mmol/g (CO uptake at 1 bar, 298 K)	13
3171 m^2/g (N_2)	2.0 Cu [b]	1.2 mmol/g (NO uptake at 2 mbar, 298 K)	14
3263 m^2/g (N_2)	1.2 Cu	0.5 mmol/g (C_2H_4, uptake at 13 mbar, 296 K)	15
3205 m^2/g (N_2)	1.8 Cu	1.0 mmol/g (C_2H_4 uptake at 13 mbar, 296 K)	15
2898 m^2/g (N_2)	2.2 Cu	1.0 mmol/g (C_2H_4 uptake at 13 mbar, 296 K)	15
2758 m^2/g (N_2)	2.3 Cu	1.1 mmol/g (C_2H_4 uptake at 13 mbar, 296 K)	15
2961 m^2/g (N_2)	3.0 Cu	1.4 mmol/g (C_2H_4 uptake at 13 mbar, 296 K)	15
2065 m^2/g (N_2)	3.8 Cu	Not quantified	15
3916 m^2/g (Ar)	2.1 Cu	1.7 mmol/g (CO uptake at 0.7 mbar, 213 K)	10
3682 m^2/g (Ar)	2.2 Cu	0.5 mmol/g (O_2 uptake at <10 mbar, 183 K)	12
3944 m^2/g (Ar)	1.0 Cu	0.5 mmol/g (O_2 uptake at < 1 mbar, 193 K)	12
2615 m^2/g (Ar)	2.6 Cu	1.5 mmol/g (O_2 uptake at < 1 mbar, 213 K)	12
4000 m^2/g (N_2)	2.9 Cu	Not quantified	16
Not Reported	2.2 Cu	1.08 mmol/g (H_2 uptake at 1 mbar, 77 K)	17
Not Reported	2.2 Cu	1.26 mmol/g (H_2 uptake at 1 mbar, 77 K)	2
Not Reported	4.0 Cu	Not quantified	18
Not Reported	2.0 Cu	~1.2 mmol/g (H_2 uptake at 1 mbar, 77 K)	19
3820(30) m^2/g (N_2)	2.4 Cu	2.03 mmol/g (H_2 uptake at 1 mbar, 77 K)	This Work
4160(40) m^2/g (N_2)	2.7 Cu	2.09 mmol/g (H_2 uptake at 1 mbar, 77 K)	This Work

[a] The pressures and capacity values used to measure the copper(I) loading were either taken directly from the references or calculated from reported single-component isotherm data using the online program WebPlotDigitizer (https://automeris.io/WebPlotDigitizer/).

[b] The Cu loading was not measured in this reference but assumed to be 2.0 Cu per node based on the synthetic procedure as described by Volkmer and coworkers in Reference 10.

Table S3. Comparison of O_2 and N_2 adsorption data reported in this work for $Cu_{2.7}$-MFU-4*l* with available literature data for other MOFs. Capacities are reported in units of mmol/g, based on O_2 and N_2 uptake at 210 and 780 mbar, respectively, from single-component isotherm data collected at 298 K. Enthalpies of adsorption (ΔH_{ads}) (or $-Q_{st}$ in the case of $K_xFe_2(bdp)_3$) are reported in units of kJ/mol.

Framework	O_2 Capacity (ΔH_{ads})	N_2 Capacity (ΔH_{ads})	Results from Cycling	IAST Selectivity at 298 K	Comments	Ref.
Cr-BTT	2.2 (−65)	0.5 (−15.3)	56% capacity loss after 15 cycles	>2570	Desorption of O_2 requires heating to 423 K	20
$Cr_3(btc)_2$	3.4 (not reported)	0.6 (not reported)	35% capacity loss after 15 cycles	Not reported	Sluggish desorption kinetics; sensitive to moisture with loss of crystallinity	21
$Co_2(OH)_2(bbta)$[c]	0.3 [22] and 0.7 [23] (−49(2))	0.2 (−14) [23]	No data reported	~49[23]		22, 23
Mn-PCN-224	0.4 (−49.6(8))	Not reported	No data reported	Not reported	–	24
Ti-MIL-101	0.9 (not reported)	Not reported	Irreversible oxidation	Not reported	Irreversible O_2 uptake	25
$K_xFe_2(bdp)_3$	~0.3–0.8 ($-Q_{st}$ >200)*	0.1 (not reported)	>50% capacity loss after one cycle for $K_{1.02}Fe_2(bdp)_3$	Not reported	Sluggish kinetics, water sensitive	26
$Cu_{2.7}$-MFU-4*l*	1.51 (−57)	1.30 (−39)	>99.9% capacity retained after 40 cycles (adsorbed O_2 and N_2)	~9	Kinetic differences in desorption allow for isolation of high-purity O_2	This work

* Based on adsorption data collected for temperatures 453 K and higher.

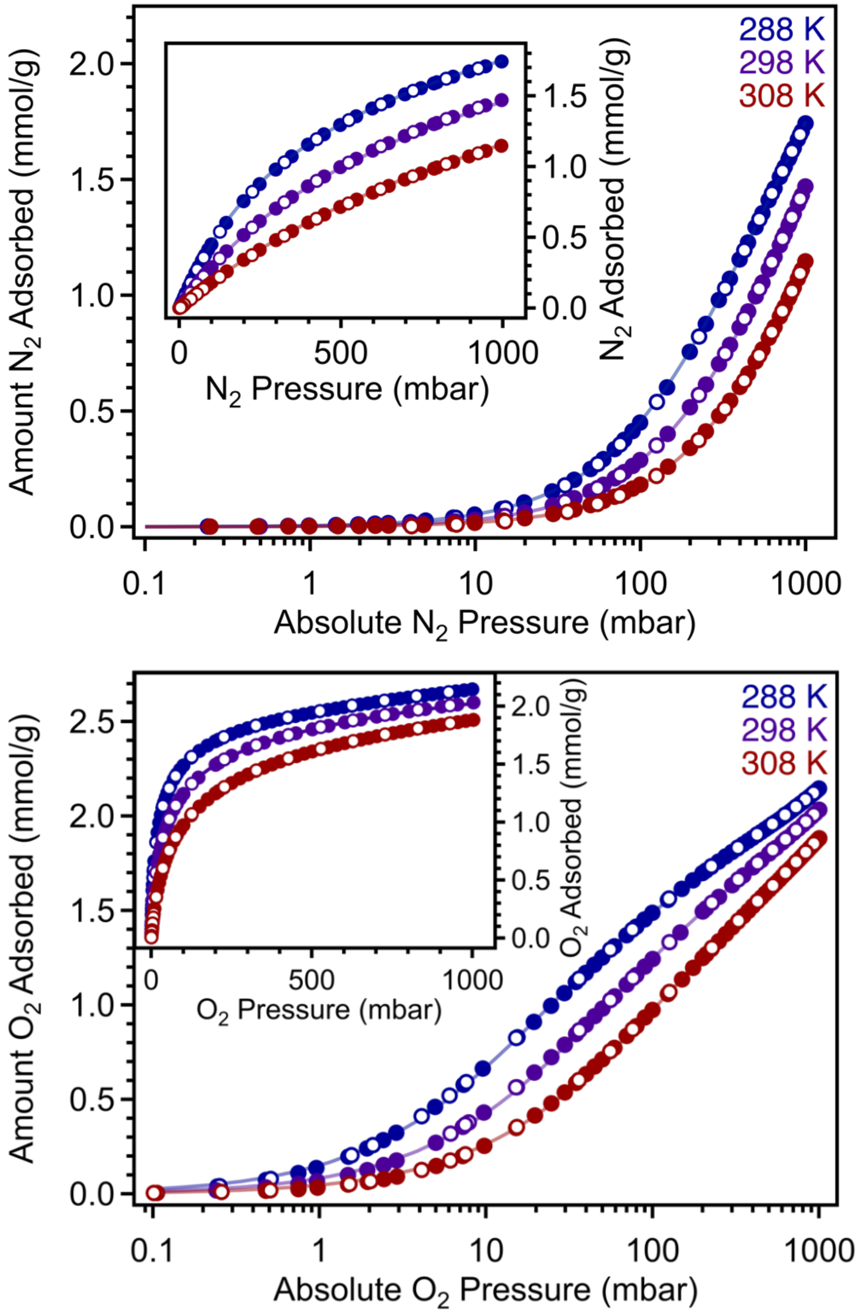

Figure S5. (Upper) Semi-logarithmic N_2 adsorption (filled circles) and desorption (open circles) isotherms for $Cu_{2.7}$-MFU-4*l* collected at 288, 298, and 308 K. (Lower) Semi-logarithmic O_2 adsorption (filled circles) and desorption (empty circles) isotherms for $Cu_{2.7}$-MFU-4*l* collected at 288, 298, and 308 K. In both plots, fits to a dual-site Langmuir-Freundlich model are shown as solid curves. The Langmuir-Freundlich equation was applied to adsorption data only, and desorption data are depicted to show reversibility and absence of hysteresis. Insets depict linear axes.

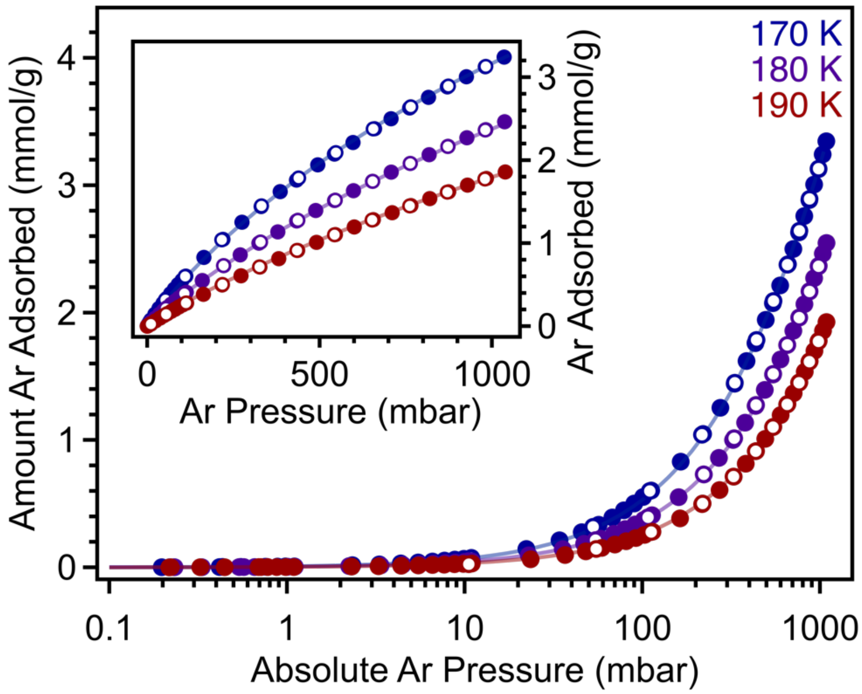

Figure S6. Semi-logarithmic Ar adsorption (filled circles) and desorption (empty circles) isotherms for $Cu_{2.7}$-MFU-4*l* collected at 170, 180, and 190 K with fits to a single-site Langmuir-Freundlich model as solid lines. The Langmuir-Freundlich equation was applied to adsorption data data only, and with desorption data are depicted to show reversibility and absence of hysteresis. Inset depicts linear axes.

Table S4. Fit parameters resulting from single-site (Ar) and dual-site (N_2 and O_2) Langmuir–Freundlich fits of N_2 (288, 298, 308 K), O_2 (288, 298, 308 K), and Ar (170, 180, and 190 K) adsorption isotherms collected for $Cu_{2.7}$-MFU-4*l*.

$Cu_{2.7}$-MFU-4*l*	Nitrogen (**N_2**)	Oxygen (**O_2**)	Argon (**Ar**)
$q_{sat,1}$	1.819	1.810	10.205
$-E_1$ (kJ/mol)	43.79	45.95	9.99
$-S_1/R$	7.69	16.01	7.87
b (bar^{-1})	0.33[a], 0.60[b], 1.06[c]	0.04[a], 0.08[b], 0.15[c]	0.45[d], 0.30[e], 0.21[f]
v_1	1.025	0.806	0.915
$q_{sat,2}$	4.246	0.863	–
$-E_2$ (kJ/mol)	12.83	19.94	–
$-S_2/R$	7.69	8.45	–
b (bar^{-1})	14.55[a], 12.3[b], 10.28[c]	1.13[a], 1.49[b], 1.94[c]	– / – / –
v_2	0.958	1.054	–

[a]288 K, [b]298 K, [c]308 K, [d]170 K, [e]180 K, [f]190 K.

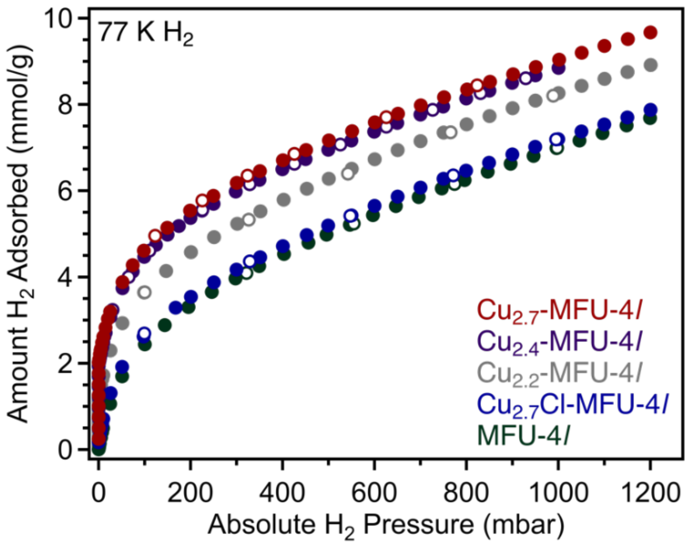

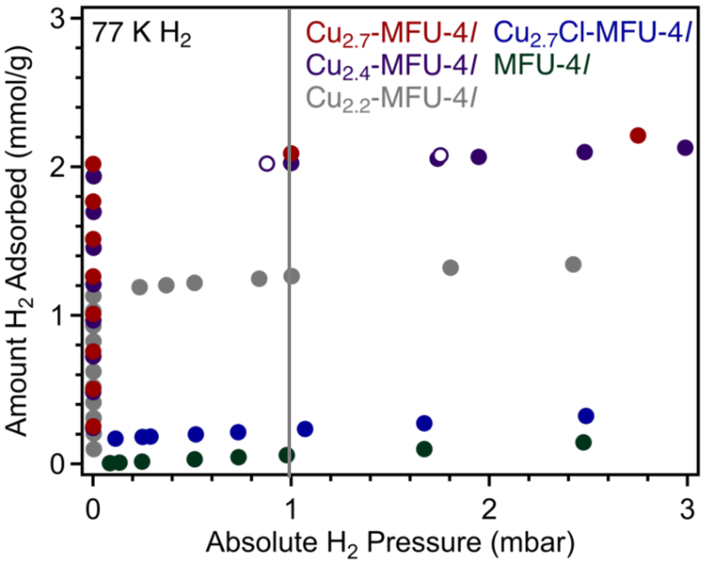

Figure S7. (Upper) Hydrogen adsorption (filled circles) and desorption (open circles) isotherms collected at 77 K for MFU-4*l* (green), $Cu_{2.7}$Cl-MFU-4*l* (blue), $Cu_{2.2}$-MFU-4*l* (gray) synthesized following the route in reference 2, $Cu_{2.7}$-MFU-4*l* (red), and $Cu_{2.4}$-MFU-4*l* (purple). Initial steep uptake at low pressures is attributed to strong binding of H_2 at the coordinatively unsaturated Cu^I sites. (Lower) Expanded view of the low-pressure region of the H_2 isotherms highlighting the uptake at 1 mbar (see the vertical gray line), which is proposed here as a benchmark for determining the loading of copper(I) sites in these materials. It should be emphasized that estimates of copper(I) loading derived in this way are qualitative only, given that some hydrogen physisorption is expected even at 77 K and 1 mbar.

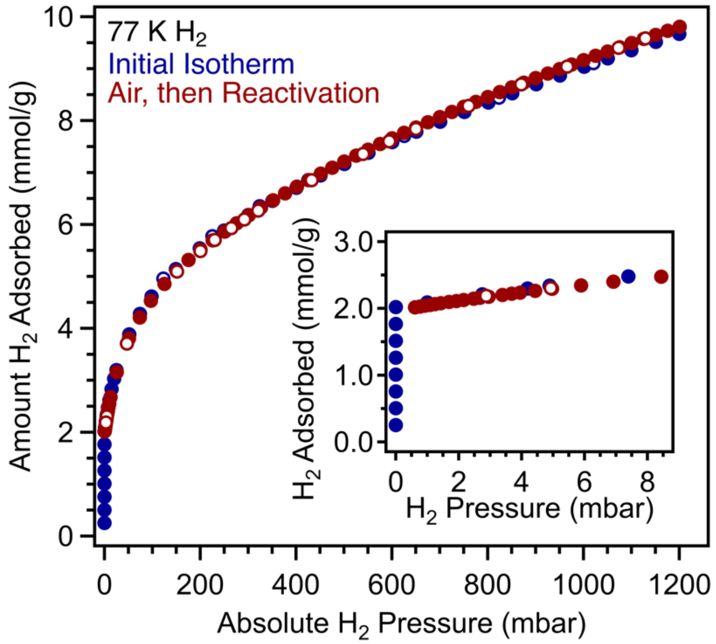

Figure S8. Hydrogen adsorption (filled circles) and desorption (open circles) isotherms collected at 77 K for $Cu_{2.7}$-MFU-4*l* before (blue) and after exposure to ambient air (2 h) followed by reactivation at 180 °C for 1 h (the sample was heated from ambient temperature to 180 °C at a 5 °C/min). The similar isotherm profiles suggest that the material is stable to air exposure under the studied conditions. The increased capacity at 1.2 bar for the reactivated sample is within measurement error from the initial isotherm. Inset depicts low-pressure data points.

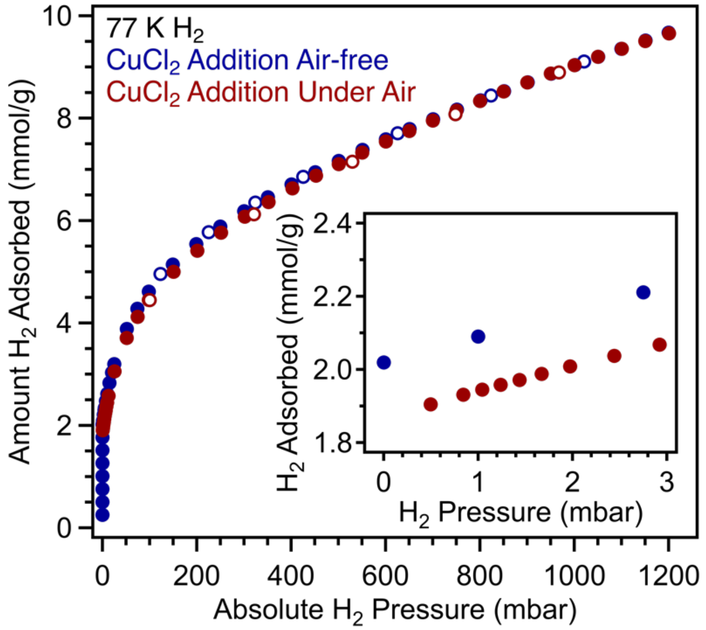

Figure S9. Hydrogen adsorption (filled circles) and desorption (open circles) isotherms collected at 77 K for $Cu_{2.7}$-MFU-4*l* synthesized with or without exclusion of air and moisture for Cu^{II} exchange (red or blue, respectively). Inset depicts low pressure data points, revealing a capacity of 1.94 mmol/g at 1 mbar H_2. Details of the syntheses are found above.

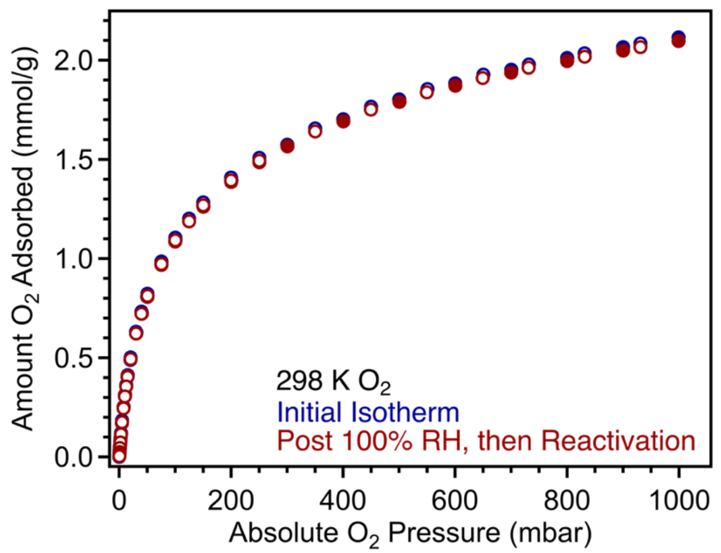

Figure S10. Oxygen adsorption (filled circles) and desorption (open circles) isotherms collected at 298 K for $Cu_{2.7}$-MFU-4*l* before (blue) and after exposure to air saturated with water for 30 minutes (red), followed by reactivation under dynamic vacuum at 150 °C for 2 h (the sample was heated from ambient temperature to 150 °C at a 1 °C/min). The similar isotherm profiles suggest that the material is stable to 100% relative humidity in air. We note that complete reactivation of the material is necessary to avoid desorption of water during the isotherm measurement, which would impact the measured capacity.

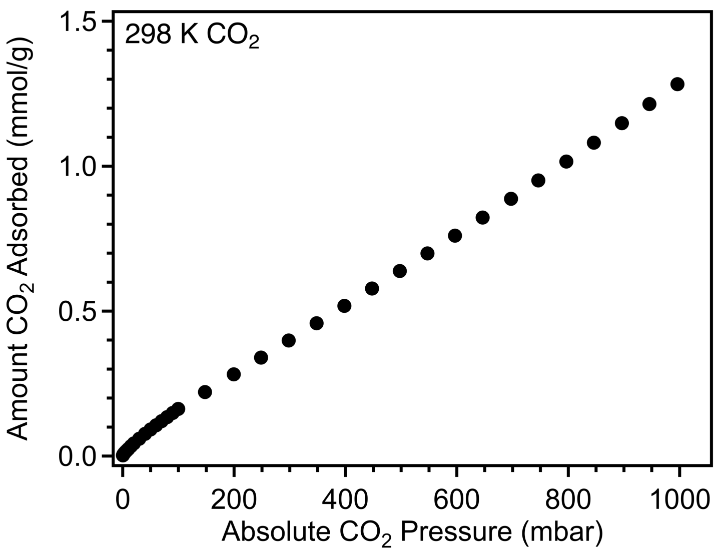

Figure S11. Carbon dioxide adsorption isotherm collected at 298 K for $Cu_{2.7}$-MFU-4*l*. The low uptake of CO_2 at concentrations relevant to air (0.003 mmol/g, 420 ppm) reflects a negligible affinity of the framework for CO_2 upon air exposure.

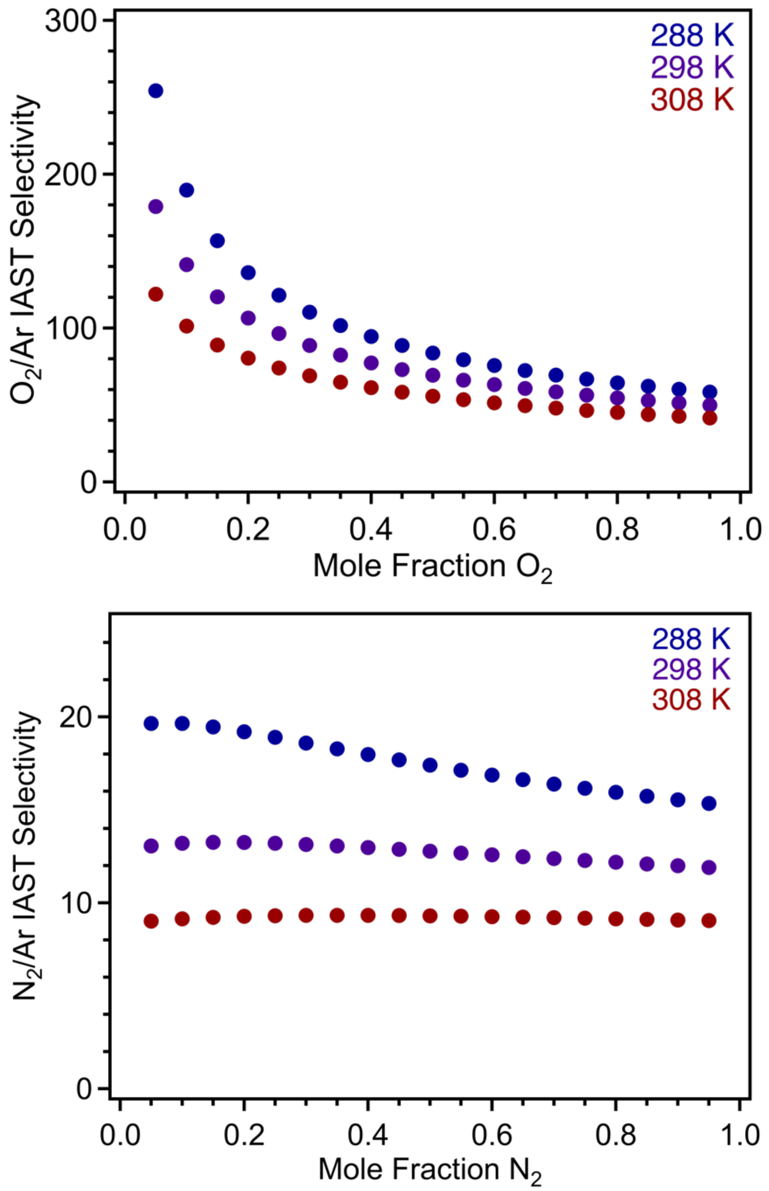

Figure S12. Selectivity values calculated using IAST for O_2 purity in mixed O_2/Ar streams at 288, 298, and 308 K (upper) and for N_2 purity in mixed N_2/Ar streams at 288, 298, and 308 K (lower). As discussed in the main text, because O_2 binds through electron transfer while Ar engages in minimal non-specific physisorption interactions with the framework, the assumptions of IAST are not necessarily valid for the chosen gas pairs,[27] and the above predictions should only be interpreted qualitatively.

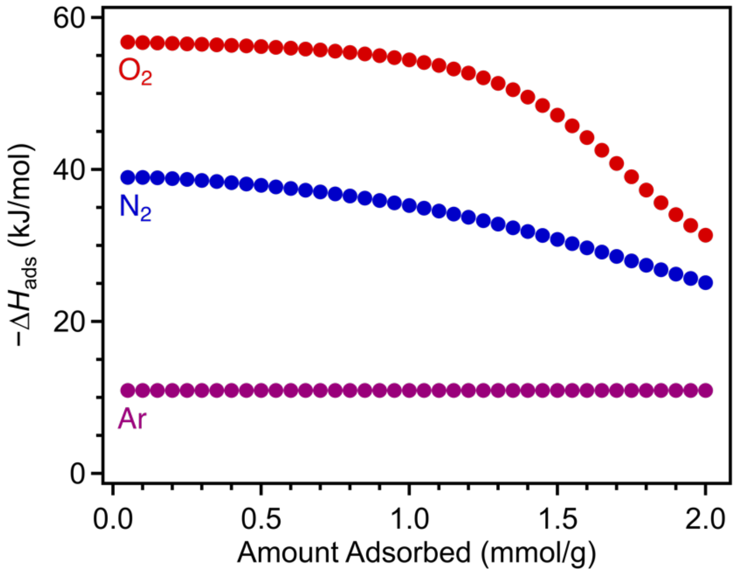

Figure S13. Isosteric enthalpies of adsorption for O_2, N_2, and Ar for $Cu_{2.7}$-MFU-4*l*. See Figure S5 for O_2 isotherms, Figure S5 for N_2 isotherms, and Figure S6 for Ar isotherms.

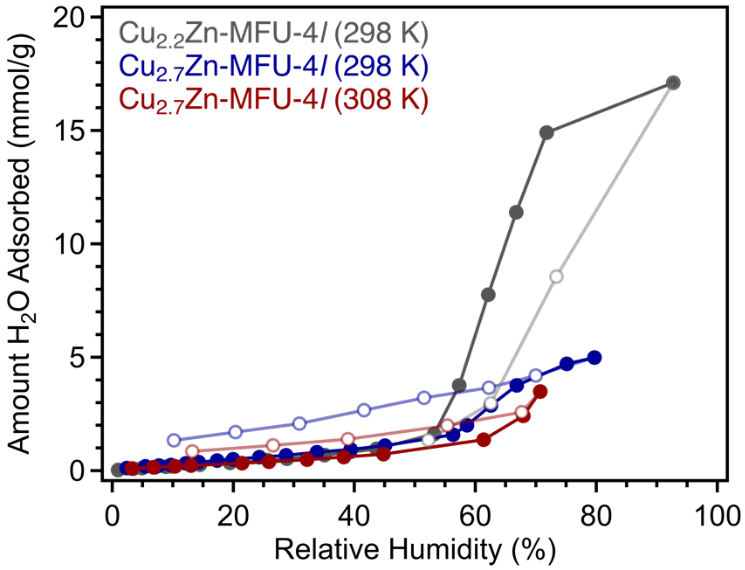

Figure S14. Single-component water adsorption (filled circles) and desorption (empty circles) isotherms collected at pressures ranging from 0.30 mbar (1% relative humidity) to 29 mbar (93% relative humidity) for $Cu_{2.2}$-MFU-4*l* at 298 K, 0.75 mbar (2% relative humidity) to 25 mbar (80% relative humidity) for $Cu_{2.7}$-MFU-4*l* at 298 K, and 1.36 mbar (2% relative humidity) to 30 mbar (53% relativity humidity) for $Cu_{2.7}$-MFU-4*l* at 308 K. The material $Cu_{2.2}$-MFU-4*l* prepared from reference 2 in our hands adsorbs much more water than $Cu_{2.7}$-MFU-4*l*. Additionally, desorption of water from the material occurred with negative hysteresis, which may indicate material decomposition. Indeed, powder x-ray diffraction data collected for the material following water desorption revealed diminished crystallinity, which is not the case for $Cu_{2.7}$-MFU-4*l* (Figure S38).

4. Supporting Isobaric Characterization Data (Thermogravimetric Analysis)

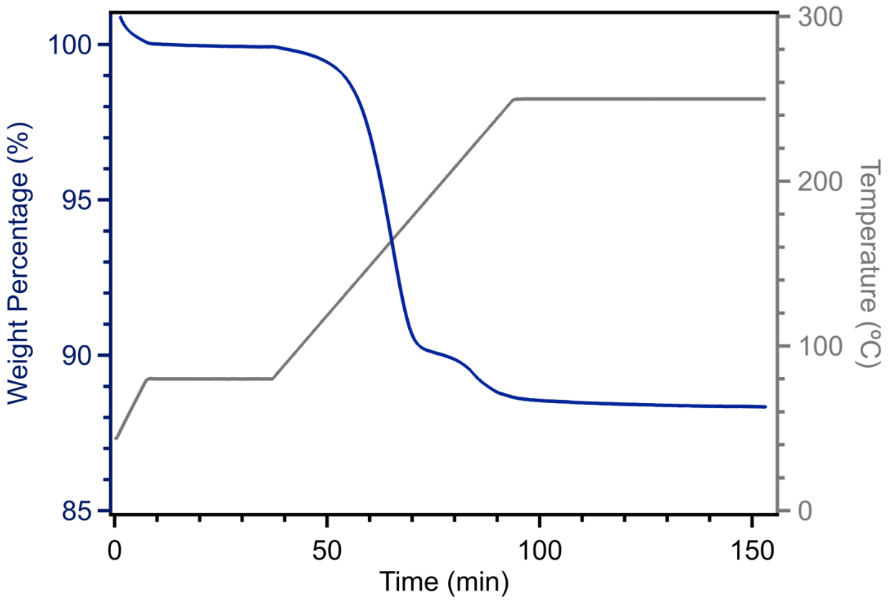

Figure S15. Thermogravimetric analysis data for conversion of $Cu_{2.7}$(OOCH)-MFU-4*l* to $Cu_{2.7}$-MFU-4*l* under N_2 at a variable ramp rate (5.0 °C/min to 80 °C, 3.0 °C/min to 250 °C). The initial decrease in mass prior to 80 °C is attributed to removal of volatiles from the framework. The measured mass loss between 100 and 250 °C corresponds to a 11.4 wt% change, consistent with a predicted 11.4 wt% loss based on the changes in mass from the chemical formula $(Cu^{II}(OOCH))_{2.4}(Cu^{II}Cl)_{0.3}(Zn(OOCH))_{0.9}(ZnCl)_{0.4}(btdd)_3$ to the chemical formula $Cu_{2.7}Zn_{2.3}H_{0.9}Cl_{0.7}(btdd)_3$. The initial steep drop (100–180 °C) is attributed to deformylation of Cu^{II} sites to yield terminal Cu^{I} sites, and the second drop (180–250 °C) is attributed to loss of CO_2 from Zn–formate sites.

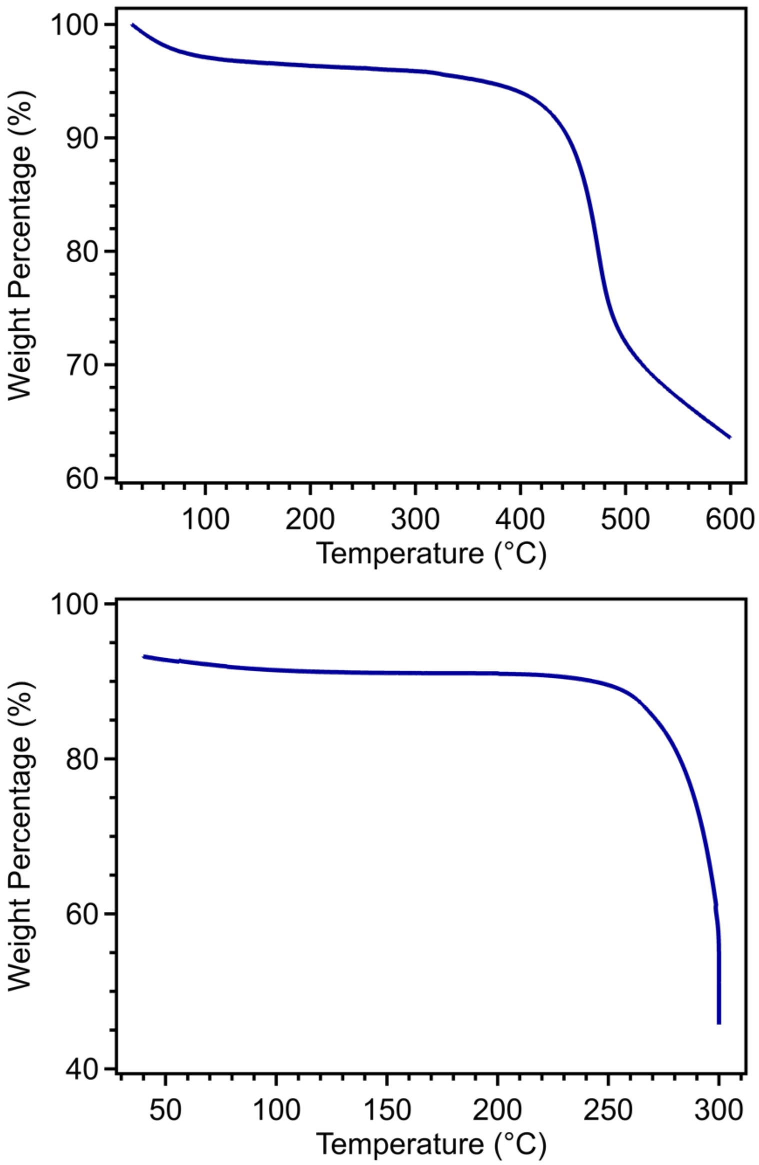

Figure S16. (Upper) Thermogravimetric analysis data collected for $Cu_{2.7}$-MFU-4*l* under N_2 with heating from 30 to 600 °C (ramp rate of 1.0 °C/min). The initial drop in weight prior to 100 °C is attributed to desorption of coordinated N_2. (Lower) Thermogravimetric analysis data collected for $Cu_{2.7}$-MFU-4*l* under O_2 with heating from 30 to 300 °C (ramp rate of 1.0 °C/min). The rapid onset weight loss at 260 °C is attributed to framework degradation. An initial small drop in weight is attributed to removal of physisorbed volatiles.

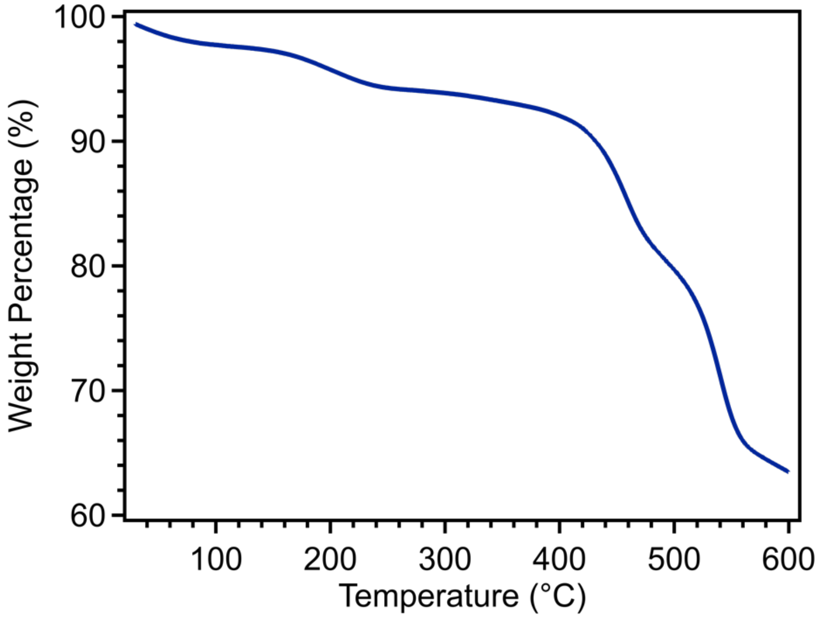

Figure S17. Thermogravimetric analysis data collected for $Cu_{2.4}$-MFU-4*l* under N_2 with heating from 30 to 600 °C (ramp rate of 1.0 °C/min). The initial drop in weight prior to 250 °C is attributed to gradual desorption of bound acetonitrile.

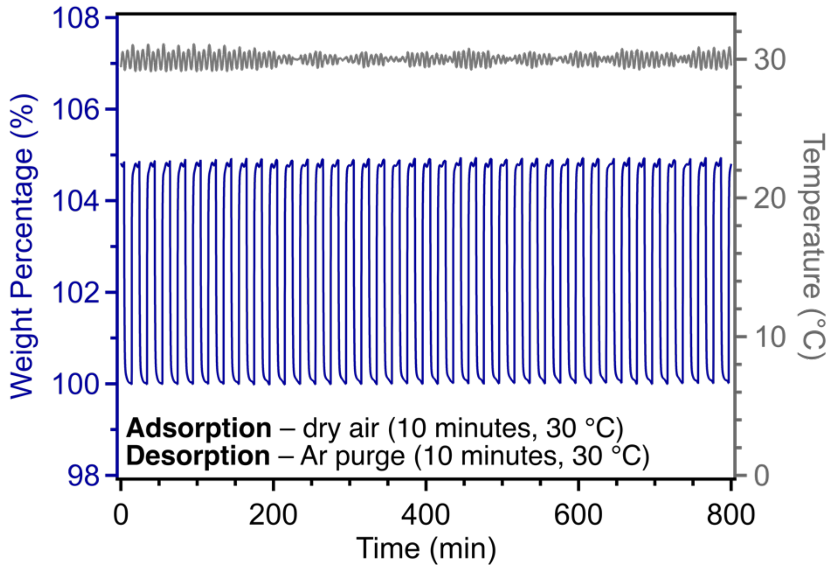

Figure S18. Crude thermogravimetric analysis (TGA) cycling data for $Cu_{2.7}$-MFU-4*l* with adsorption under air (atmospheric pressure, 10 min, 30 °C) and desorption under an Ar purge (atmospheric pressure, 10 min, 30 °C). The slight fluctuations in the peaks are attributed to minor temperature fluctuations (gray trace). No apparent change in capacity occurred over the course of cycling. Note that the total capacity reflects adsorbed O_2 and N_2.

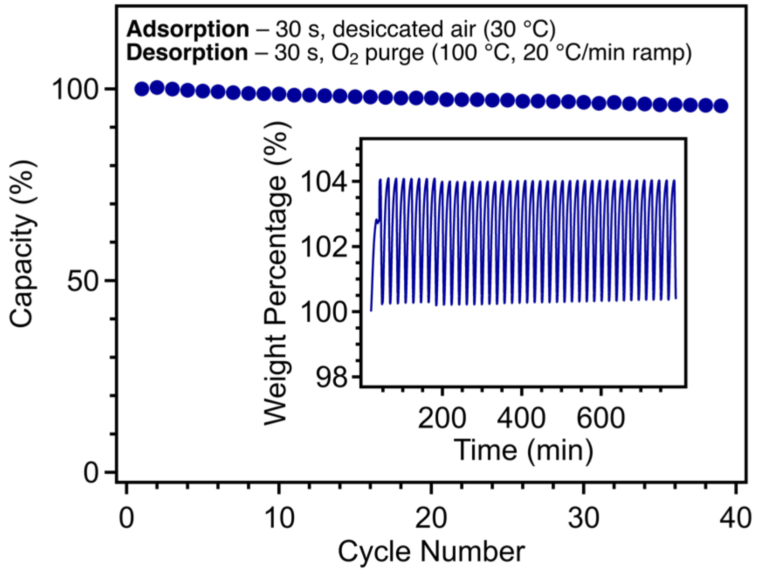

Figure S19. Relative cycling capacity of $Cu_{2.7}$-MFU-4*l* measured over the course of 40 TGA adsorption (flowing dry air, atmospheric pressure, 30 s, 30 °C) and desorption (flowing O_2, atmospheric pressure, 30 s, 100 °C) cycles. The capacity is reported relative to the measured capacity from the first cycle. (Inset) Raw TGA data. The lower cycling capacity determined here relative to that determined when cycling under simulated vacuum-swing adsorption (VSA) conditions (Figure S21) can be attributed to the shorter cycle times used in this case, incomplete O_2 desorption under these conditions, and possibly slight material degradation under the oxidizing desorption conditions.

5. Kinetic Measurements

Table S5. Equilibrium pressures (in mbar) following dosing of $Cu_{2.7}$-MFU-4*l* with varying loadings of O_2 and N_2 at 288, 298, and 308 K for kinetic measurements.

O_2 loading	Pressure at 288 K	Pressure at 298 K	Pressure at 308 K
0.5 mmol/g	3.1	5.5	8.3
1.0 mmol/g	8.7	13.4	18.7
5.0 mmol/g	116.4	125.1	134.7
10.0 mmol/g	282.8	289.5	298.6
**N_2 loading**	**Pressure at 288 K**	**Pressure at 298 K**	**Pressure at 308 K**
0.5 mmol/g	14.2	15.4	16.2
1.0 mmol/g	28.8	31.1	32.4
5.0 mmol/g	150.8	158.6	165.7
10.0 mmol/g	310.1	321.9	333.1

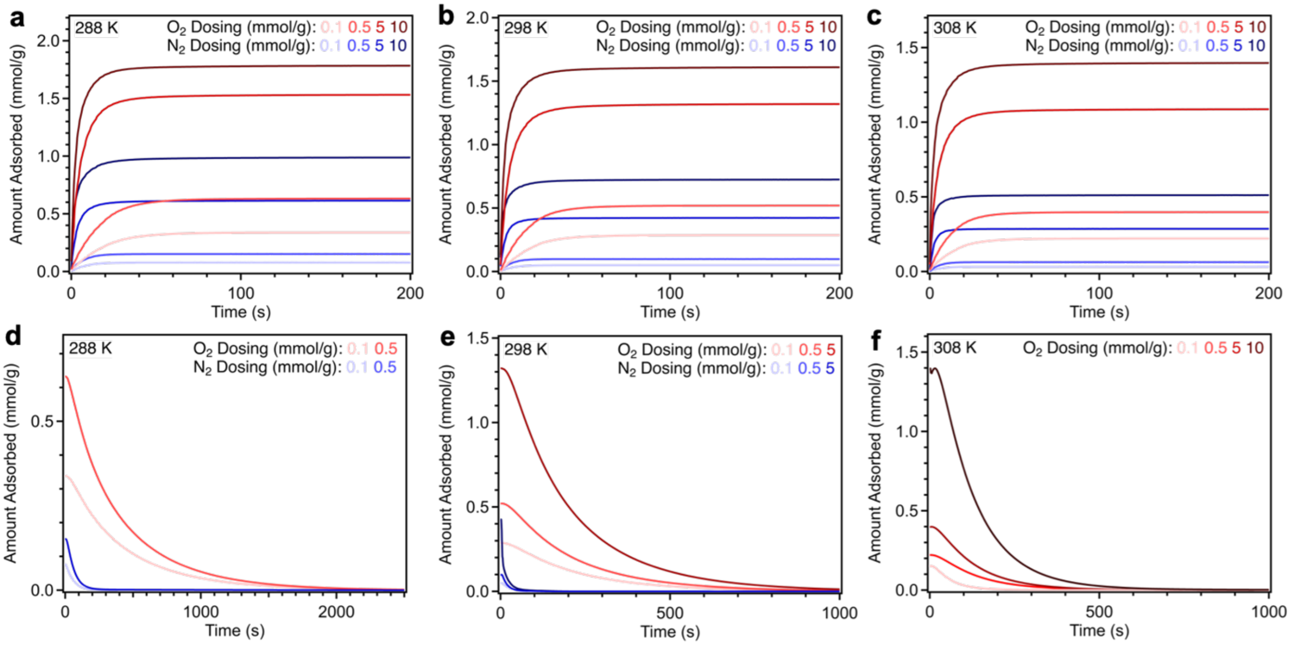

Figure S20. Kinetic adsorption traces of O_2 and N_2 to $Cu_{2.7}$-MFU-4*l* at temperatures (a) 288 K, (b) 298 K, and (c) 308 K. Plateau values indicate pressure equilibration. Kinetic desorption traces of O_2 and N_2 at temperatures (d) 288 K, (e) 298 K, and (f) 308 K. Plateau values indicate complete material regeneration.

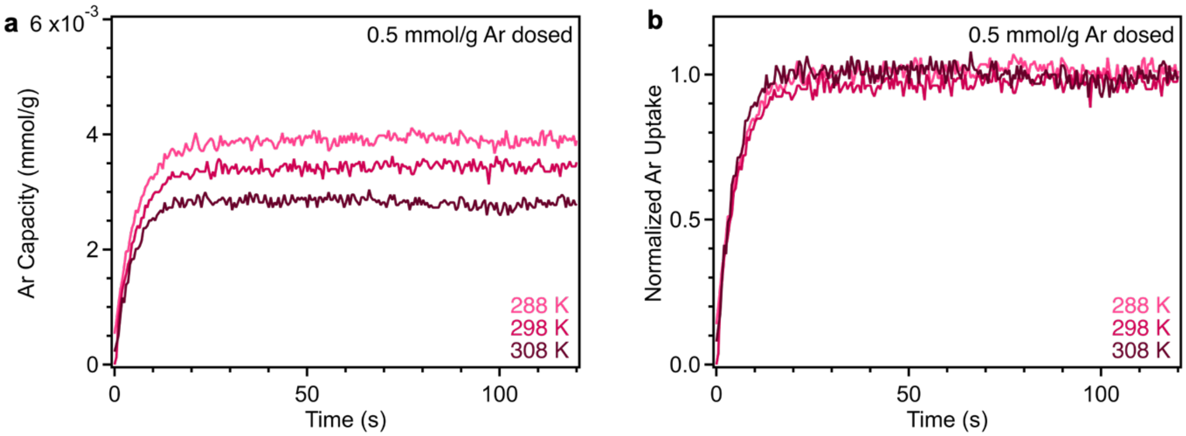

Figure S21. (a) Kinetic Ar adsorption traces for $Cu_{2.7}$-MFU-4*l* at 288, 298, and 308 K. (b) Normalized Ar kinetic adsorption traces at 288, 298, and 308 K. Due to the minimal uptake of Ar by $Cu_{2.7}$-MFU-4*l* and the rapid desorption of bound Ar from the framework, accurate desorption kinetic traces could not be obtained.

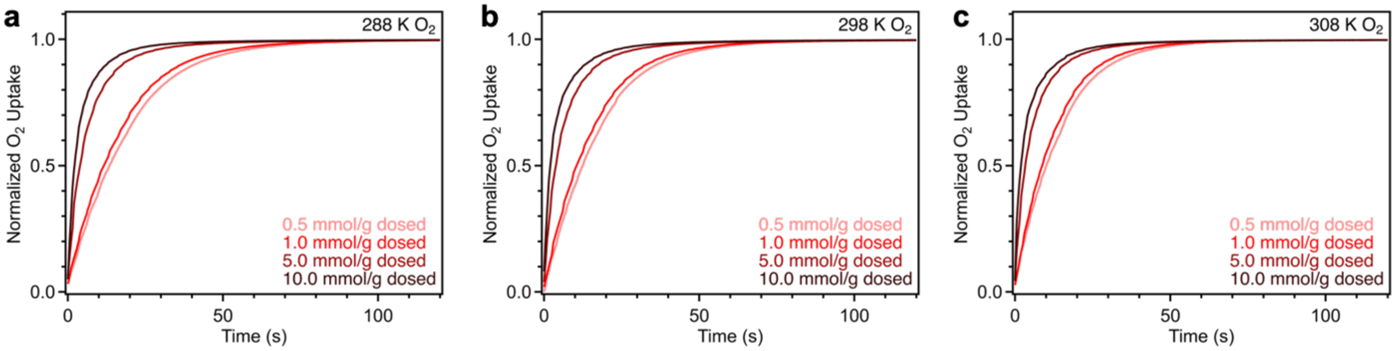

Figure S22. Normalized kinetic O_2 adsorption traces for $Cu_{2.7}$-MFU-4*l* at (a) 288 K, (b) 298 K, and (c) 308 K with variable pressures of O_2 dosing.

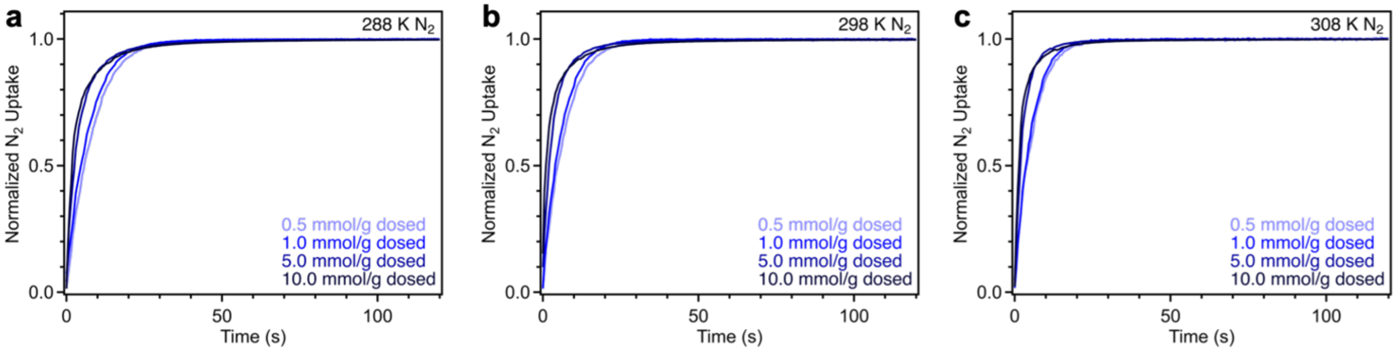

Figure S23. Normalized kinetic N_2 adsorption traces for $Cu_{2.7}$-MFU-4*l* at (a) 288 K, (b) 298 K, and (c) 308 K with variable pressures of N_2 dosing.

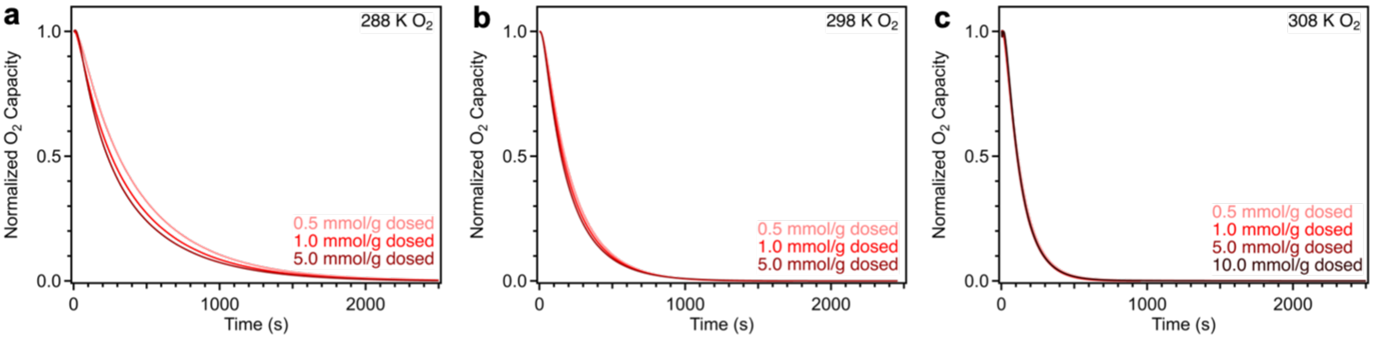

Figure S24. Kinetic O_2 desorption traces for $Cu_{2.7}$-MFU-4*l* at (a) 288 K, (b) 298 K, and (c) 308 K with variable pressures of O_2 dosing, normalized to the equilibrium O_2 capacity following adsorption measurements at the specified dosing concentrations.

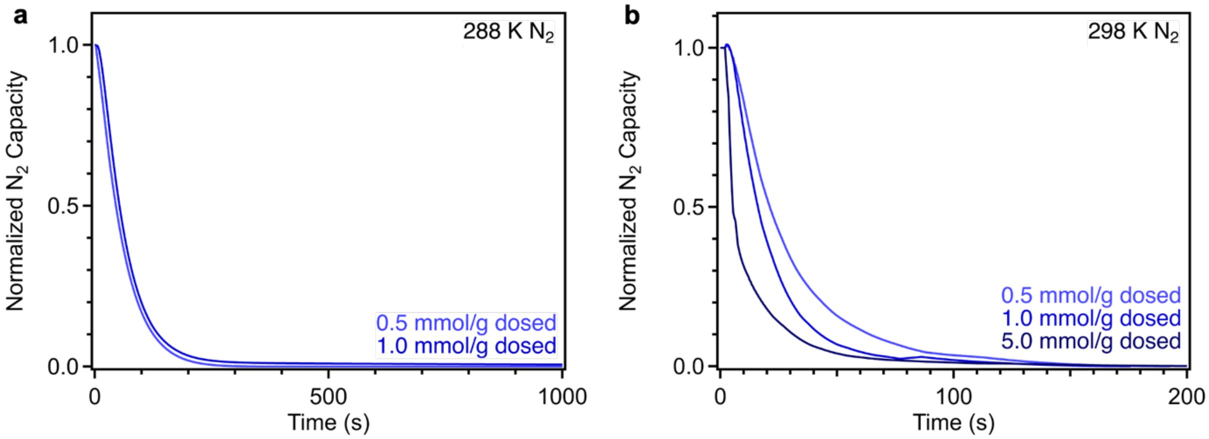

Figure S25. Normalized kinetic N_2 desorption traces of $Cu_{2.7}$-MFU-4*l* at (a) 288 K and (b) 298 K with variable pressures of N_2 dosing. Due to the rapid nature of N_2 desorption at 308 K, desorption traces devoid of poor signal-to-noise could not be obtained.

Table S6. Measured activation barriers (E_a) for O_2 and N_2 adsorption and desorption based on fits to kinetic data from Figures S20–S23.

Contribution	O_2 Adsorption	N_2 Adsorption	O_2 Desorption	N_2 Desorption
Activation Energy (E_a)	10(1) kJ/mol	12(1) kJ/mol	45(1) kJ/mol	30(1) kJ/mol

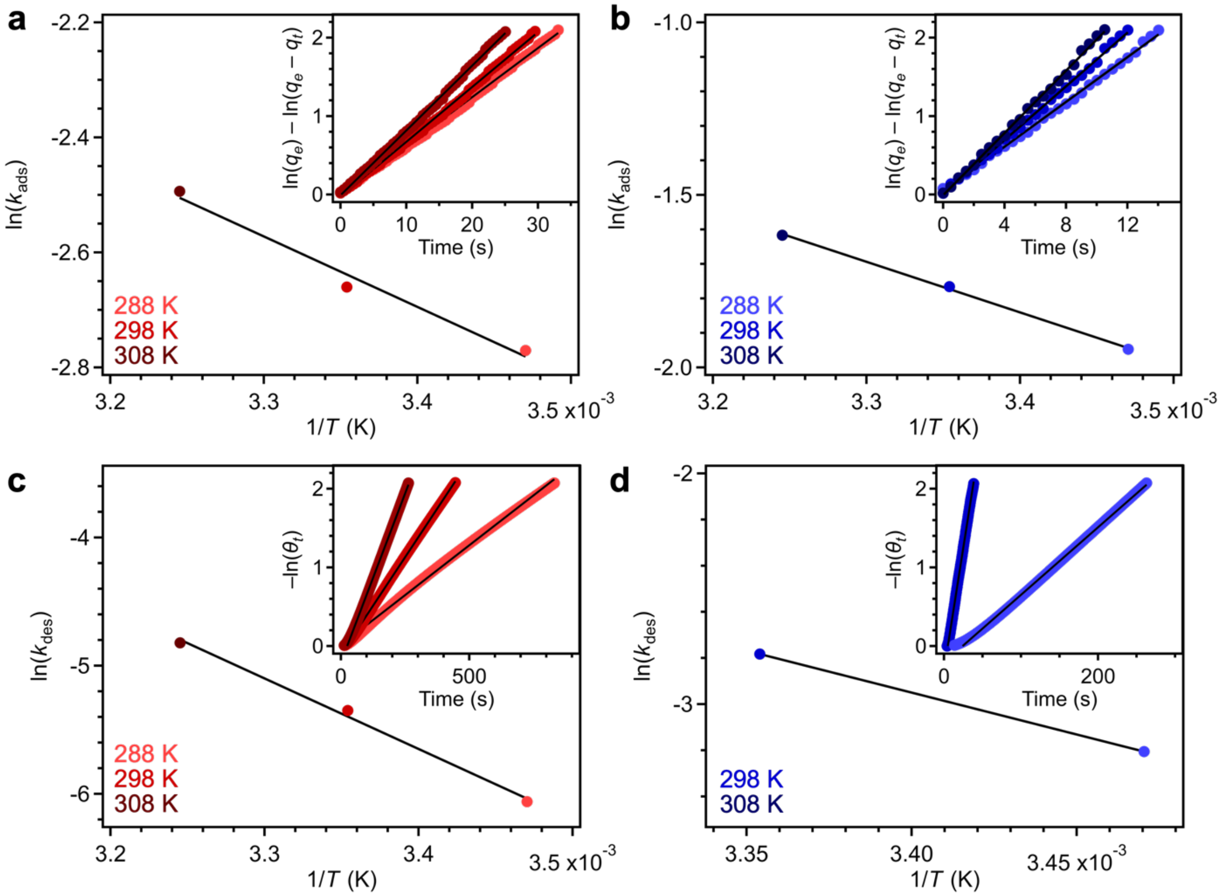

Figure S26. Arrhenius plots for (a) O_2 adsorption, (b) N_2 adsorption, (c) O_2 desorption, and (d) N_2 desorption, all following 1.0 mmol/g dosing. Insets depict fits for rate constant calculations for a pseudo-first order reaction measured to $3t_{1/2}$.

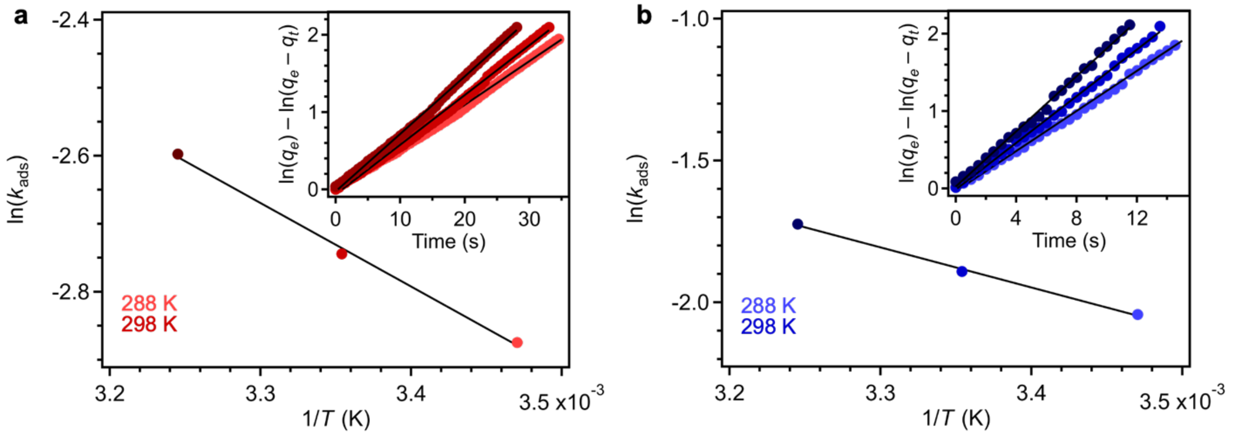

Figure S27. Arrhenius plots for $Cu_{2.7}$-MFU-4*l* with (a) O_2 adsorption and (b) N_2 adsorption with 0.5 mmol/g dosing. Insets depict fits for rate constant calculations for a pseudo-first order reaction measured to $3t_{1/2}$. Calculated E_a values for O_2 adsorption (10(1) kJ/mol) and N_2 adsorption (12(1) kJ/mol) are identical to those measured from 1.0 mmol/g dosing values.

Table S7. Intraparticle diffusion time constants (D_c/r_c^2, units of s^{-1}) for $Cu_{2.7}$-MFU-4*l* dosed with O_2 and N_2 at variable temperatures, in units of s^{-1}.

Temperature	0.5 mmol/g O_2	1.0 mmol/g O_2	0.5 mmol/g N_2	1.0 mmol/g N_2
288 K	4.8×10^{-3}	5.7×10^{-3}	1.3×10^{-2}	1.4×10^{-2}
298 K	5.8×10^{-3}	6.4×10^{-3}	1.4×10^{-2}	1.9×10^{-2}
308 K	7.1×10^{-3}	7.2×10^{-3}	1.9×10^{-2}	2.3×10^{-2}

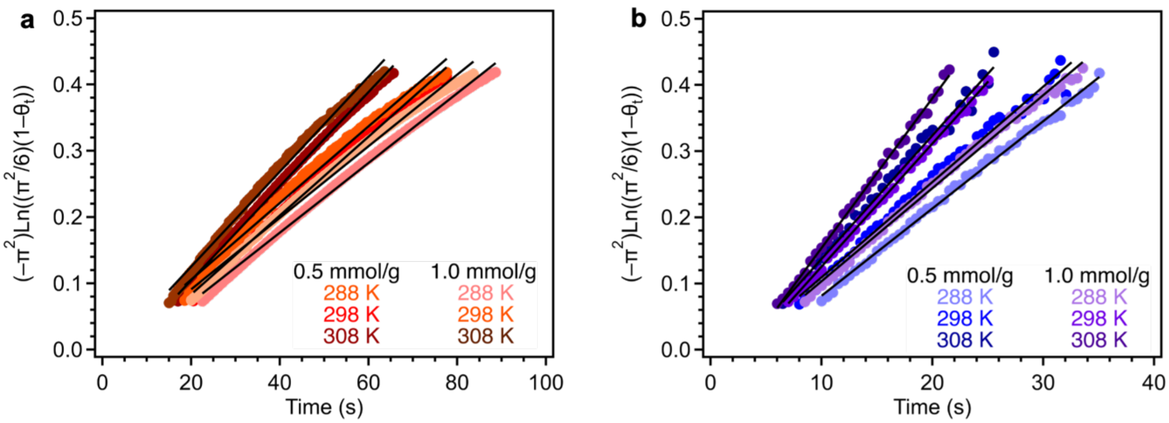

Figure S28. Intraparticle O_2 (a) and N_2 (b) diffusion time constant plots for $Cu_{2.7}$-MFU-4*l*, fit between 70 and 99% reaction completion, based on data fits as shown in Figure S24. Due to deviation of the data from linearity at high pressures, time constants should be interpreted qualitatively.

6. Supporting Spectroscopic Characterization Data

6.1 Energy Dispersive X-Ray Spectroscopy Data.

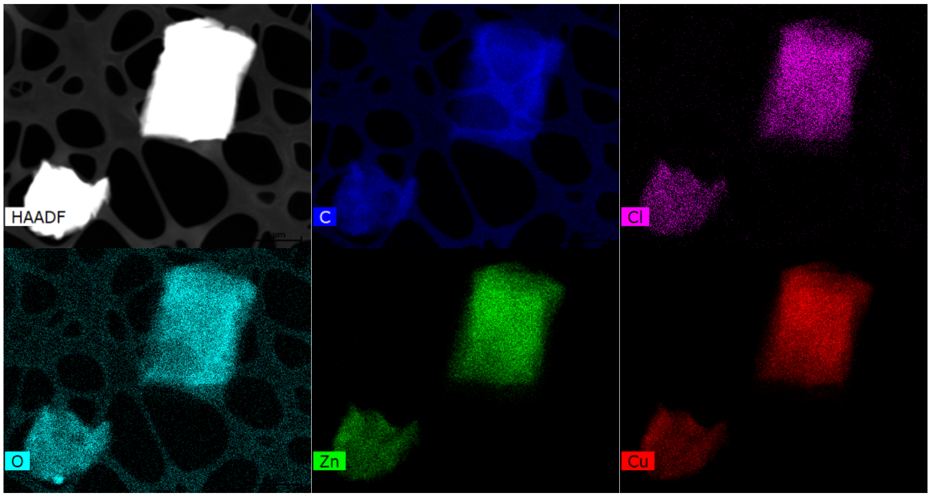

Figure S29. High-angle annular dark-field scanning transmission electron microscopy (HAADF-STEM) image (top left) of a sample of $Cu_{2.7}$-MFU-4*l* analyzed using energy-dispersive x-ray spectroscopy. Elemental maps are displayed in the remaining panels with the following color code: C (blue), Cl (pink), O (cyan), Zn (green), Cu (red). Field of view = 5 µm.

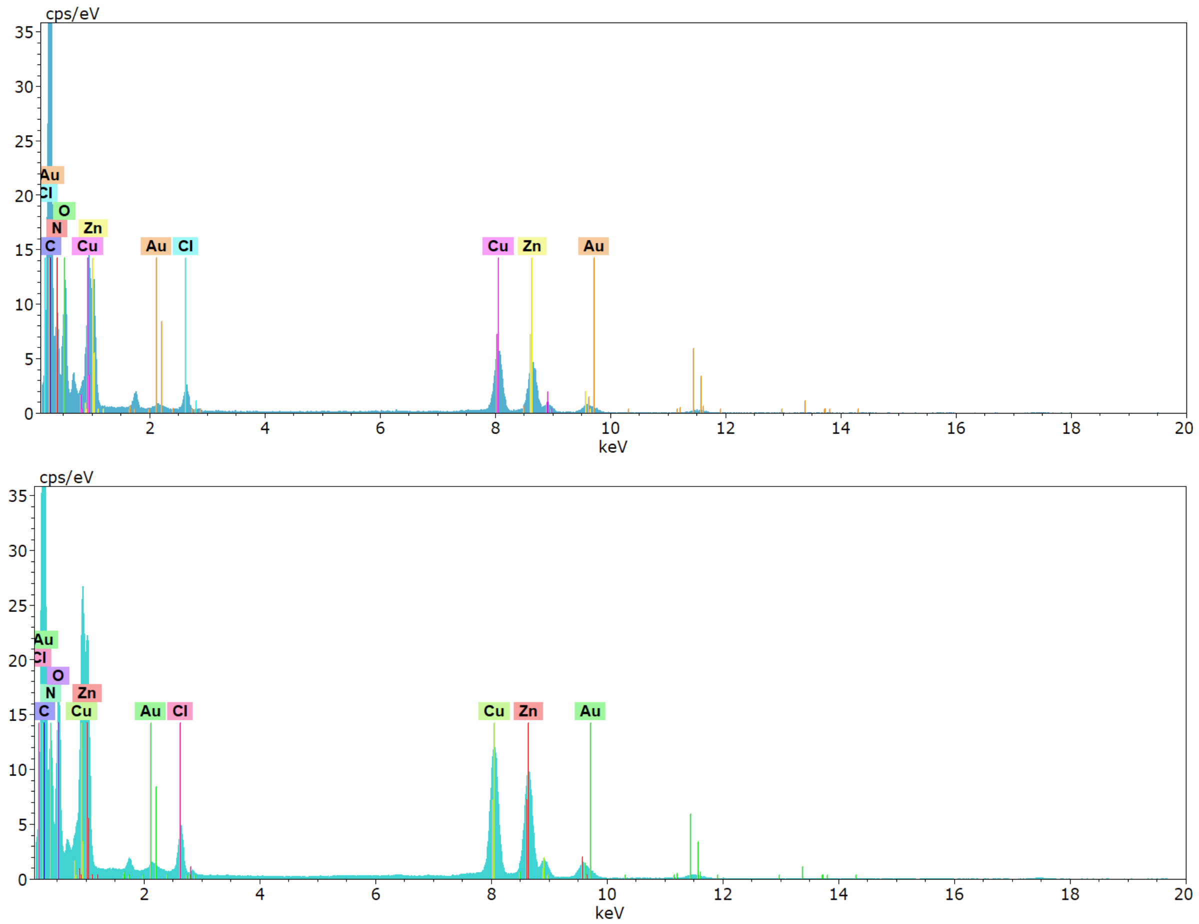

Figure S30. Energy dispersive x-ray spectroscopy results for the right particle (upper) and the left particle (lower) of $Cu_{2.7}$-MFU-4*l* shown in Figure S29.

Table S8. Energy dispersive x-ray spectroscopy results for $Cu_{2.7}$-MFU-4*l* (see Figures S27–S28).

	Element	Edge	Ratio by Weight %	Ratio by Atomic %	3σ Weight %
Left particle	Cl	K	8.1	13.9	0.9
	Cu	L	48.7	46.3	14.7
	Zn	L	43.1	39.8	13.0
	Cu	K	53.0	53.8	4.9
	Zn	K	47.0	46.2	4.4
Right particle	Cl	K	9.0	15.2	0.9
	Cu	L	48.9	46.2	14.8
	Zn	L	42.1	38.6	12.7
	Cu	K	52.9	53.6	4.9
	Zn	K	47.1	46.4	4.3

Data analysis reveals a Cu:Zn ratio of 2.7:2.3 per node, in agreement with the ratio determined for the Cu(II) precursor by ICP-OES analysis. Further analysis by EDX reveals ~0.8-0.9 Cl per formula unit, in qualitative agreement with the degree of formate substitution estimated from ^{1}H NMR spectroscopy analysis of a digested sample of $Cu_{2.7}$(OOCH)-MFU-4*l*.

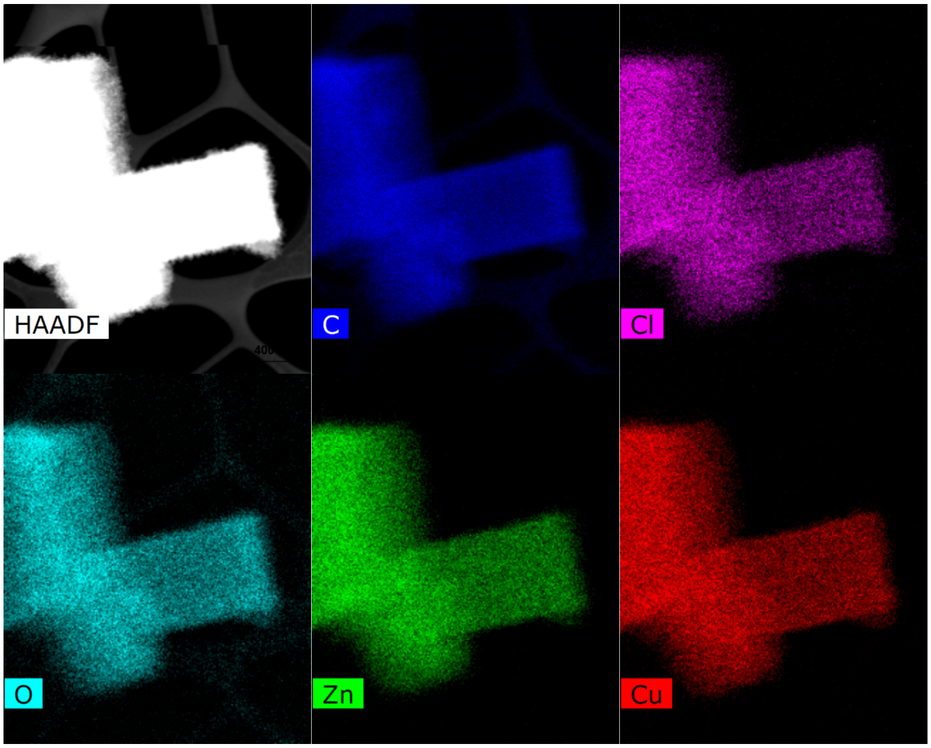

Figure S31. High-angle annular dark-field scanning transmission electron microscopy (HAADF-STEM) image (top left) of a sample of $Cu_{2.4}$-MFU-4*l* analyzed using energy-dispersive x-ray spectroscopy. Elemental maps are displayed in the remaining panels with the following color code: C (blue*),* Cl (pink), O (cyan), Zn (green), Cu (red). Field of view = 1.8 µm.

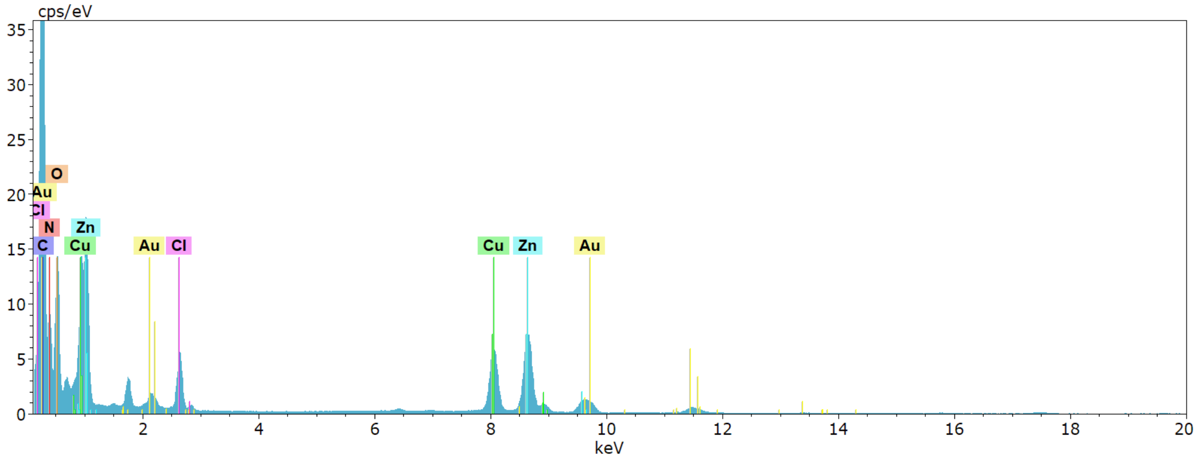

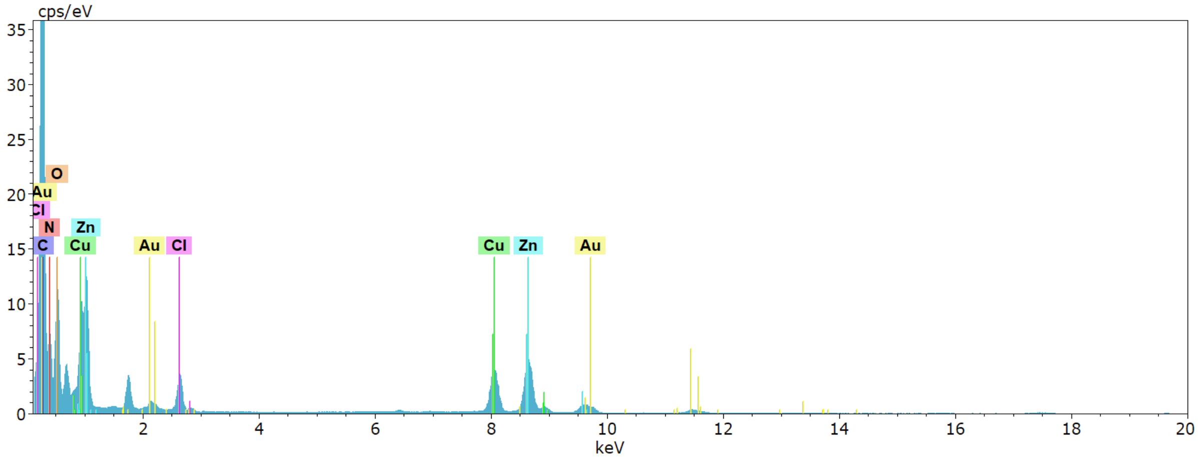

Figure S32. Energy dispersive x-ray spectroscopy results for the leftmost particle (upper) and rightmost particle (lower) of $Cu_{2.4}$-MFU-4*l*, shown in Figure S31.

Table S9. Energy dispersive spectroscopy results on leftmost and rightmost particles of $Cu_{2.4}$-MFU-4*l* from Figure S29.

	**Element**	**Element Edge**	**Ratio by Weight %**	**Ratio by Atomic %**	**3σ Weight %**
Leftmost particle	Cl	K	14.3	23.3	1.4
	Cu	L	35.6	32.4	10.8
	Zn	L	50.1	44.3	15.1
	Cu	K	42.4	43.1	3.9
	Zn	K	57.6	56.9	5.3
Rightmost particle	Cl	K	13.3	21.9	1.3
	Cu	L	37.5	34.3	11.3
	Zn	L	49.2	43.8	14.9
	Cu	K	44.2	44.9	4.1
	Zn	K	55.8	55.1	5.2

The data analysis suggests a Cu loading of *ca.* 2.2 Cu per cluster, which is marginally lower than the uptake capacity from the H_2 isotherm and the ICP-OES analysis, as the latter two forms of characterization are consistent with 2.4 Cu ions per node.

6.2. Nuclear Magnetic Resonance (NMR) Spectra.

Note: Altering acid concentration in framework degradation will subtly shift acidic ^{1}H resonances, including those of HCl and H_2btdd.

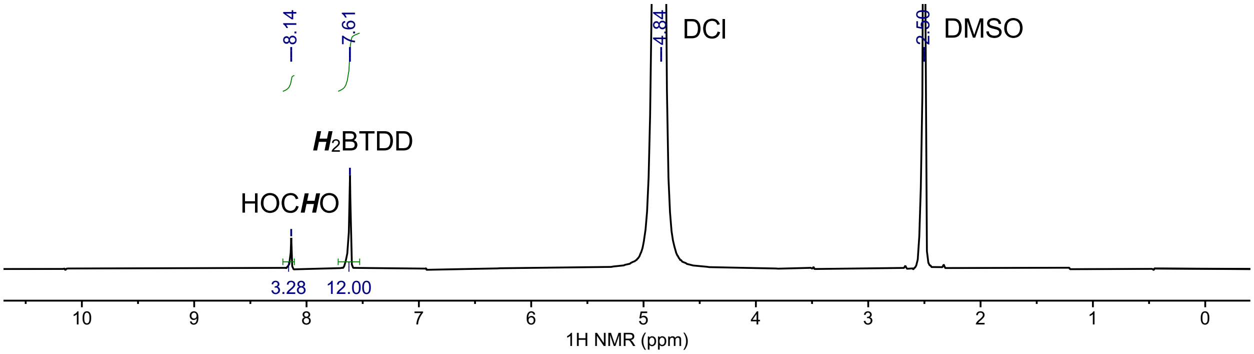

Figure S33. Proton NMR spectrum (400 MHz) collected for a digested sample of $Cu_{2.7}$(OOCH)-MFU-4*l* (dissolved in DCl/DMSO-d_6). The integration of the formic acid resonance (δ = 8.14 ppm) compared to the linker resonances (δ = 7.61 ppm) reveals ~80–85% formate incorporation. If the exchange of formate into the framework were to reach completion and afford the material $(Cu/Zn)_5(OOCH)_4(btdd)_3$, then a ^{1}H ratio of 4:12 would be expected for the integration of the formate C–*H* resonance relative to the linker C–*H* resonance.

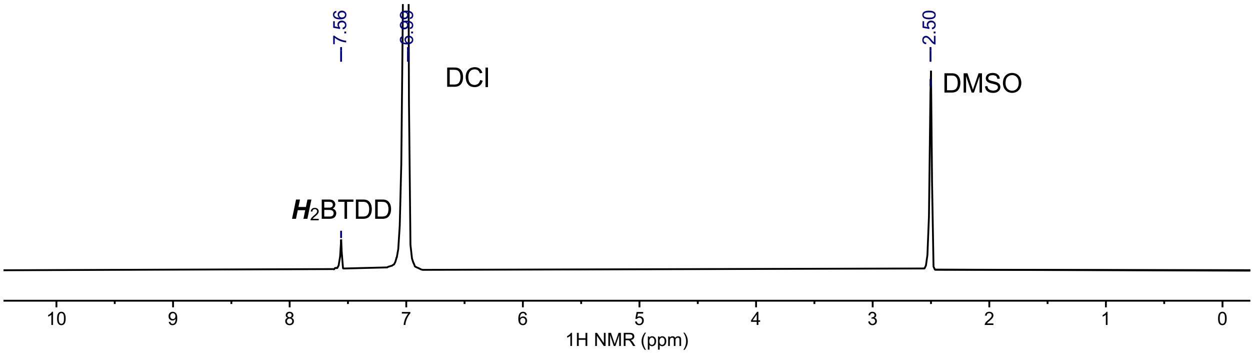

Figure S34. Proton NMR spectrum (400 MHz) of an acid-digested (DCl/DMSO-d_6) sample of $Cu_{2.7}$-MFU-4*l*. There is no peak for formic acid (expected at δ = 8.14 ppm), indicating compete conversion of Cu^{II}–OOCH and Zn–OOCHO motifs into Cu^{I} and Zn–H motifs, respectively.

6.3. Further Spectroscopic Characterization.

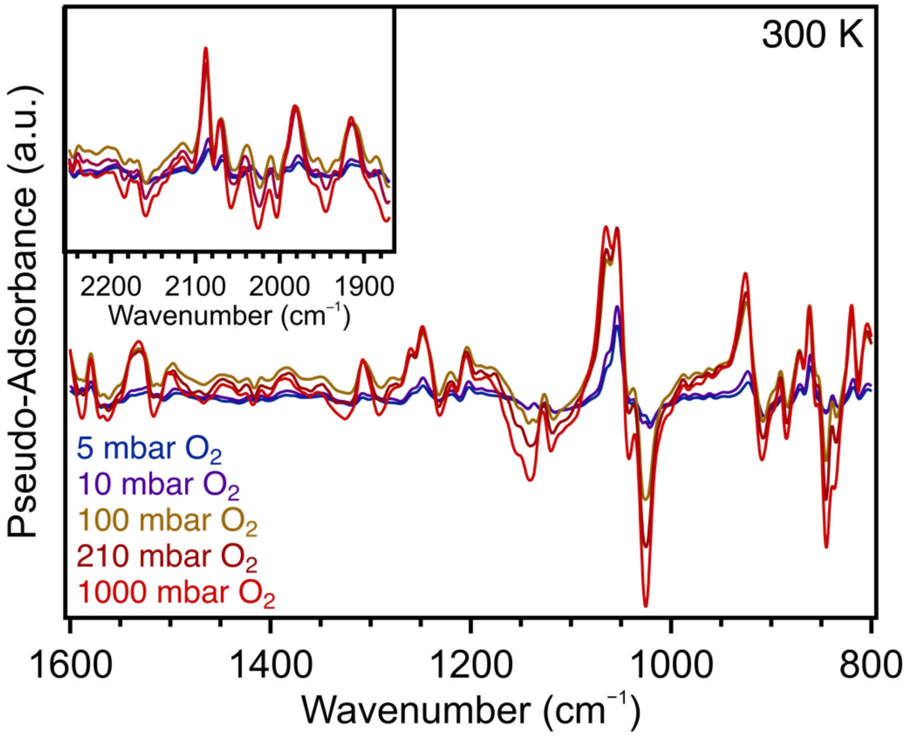

Figure S35. Difference spectra generated by subtracting DRIFTS data collected for activated $Cu_{2.4}$-MFU-4*l* from spectra collected for $Cu_{2.4}$-MFU-4*l* dosed with various pressures of O_2 at 300 K. The inset depicts the superoxide *ν*(O–O) overtone region.

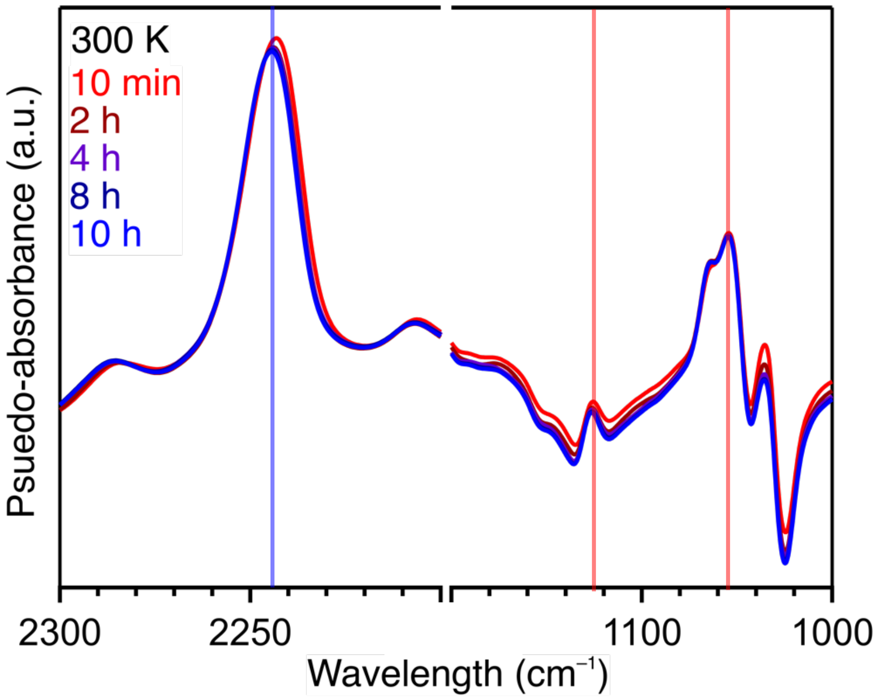

Figure S36. Difference spectra generated by subtracting DRIFTS data collected for activated $Cu_{2.4}$-MFU-4*l* from spectra collected for $Cu_{2.4}$-MFU-4*l* exposed to ambient air at 300 K (relative humidity of ~50%, measured with a LI-COR 850 humidity monitor). Salient spectroscopic features include bands for N_2 (blue vertical line, 2242 cm^{-1}) and O_2 (both red vertical lines, 1131 and 1051 cm^{-1}) adsorbed at the copper sites in the material. Importantly, the Cu–O_2 vibration persists over the 10 h span, reflecting a stability of the framework to ambient air.

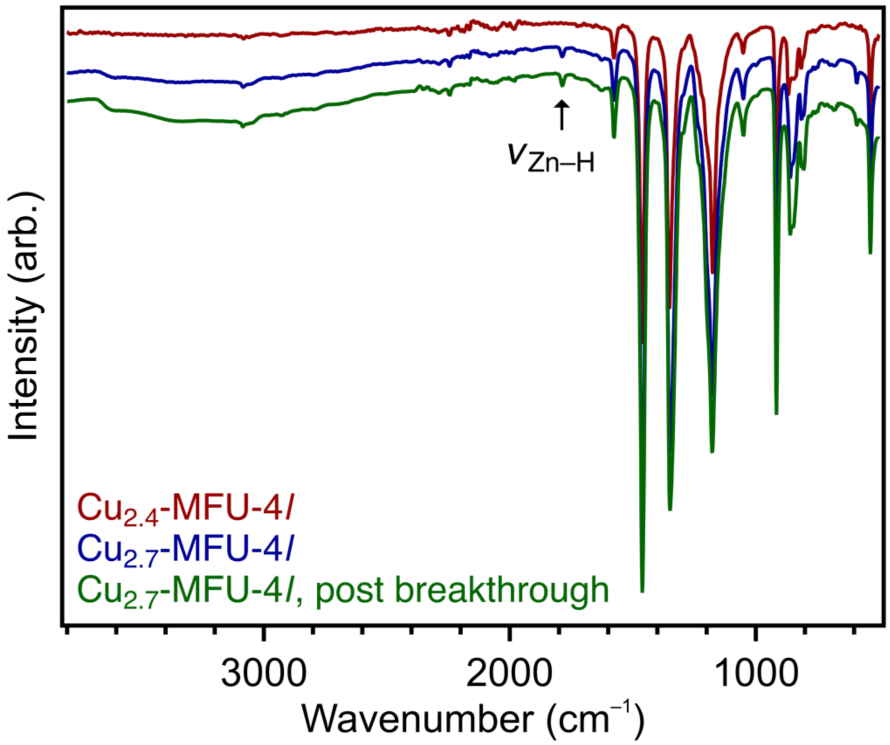

Figure S37. Comparison of infrared spectra (ATR, room temperature, under air) of $Cu_{2.4}$-MFU-4*l* (red), $Cu_{2.7}$-MFU-4*l* (blue), and $Cu_{2.7}$-MFU-4*l* (blue) following breakthrough analysis and reactivation at 150 °C under flowing He (green, following Figure S57). The black arrow denotes the Zn–H bond vibration (~1787 cm^{-1}) present in $Cu_{2.7}$-MFU-4*l* from thermolysis of Zn–OOCH motifs. Due to the absence of formate intermediates in the synthesis of $Cu_{2.4}$-MFU-4*l*, no such bond vibration is present in the $Cu_{2.4}$-MFU-4*l* spectrum. The 1787 cm^{-1} feature assignment is based on comparison to molecular Zn–H motifs.[28]

7. Supporting X-ray Diffraction Characterization Data

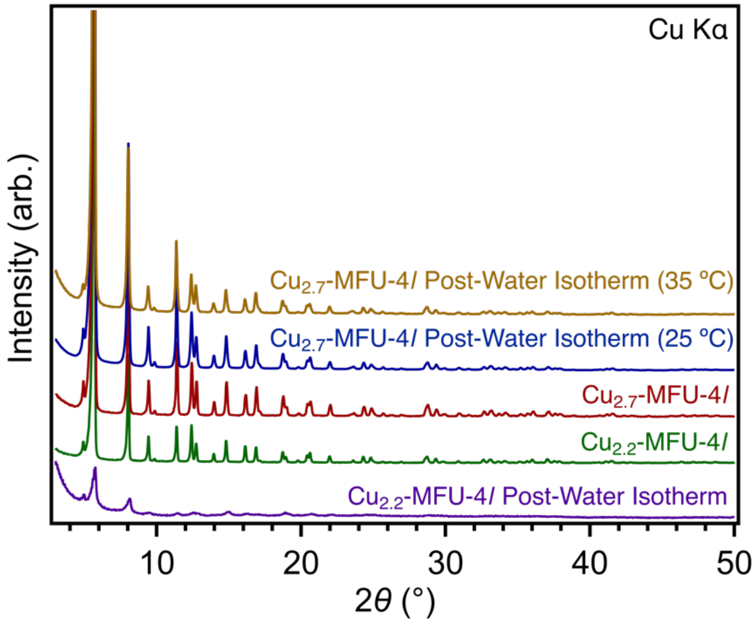

Figure S38. Powder x-ray diffraction data (Cu Kα) collected under air for $Cu_{2.2}$-MFU-4*l* prepared following the route in reference 2 (green), $Cu_{2.7}$-MFU-4*l* (red), $Cu_{2.2}$-MFU-4*l* after collecting water adsorption and desorption isotherms at 25 °C (purple), $Cu_{2.7}$-MFU-4*l* following collection of water adsorption and desorption isotherms at 25 °C (blue), and $Cu_{2.7}$-MFU-4*l* following collection of a water adsorption and desorption isotherms at 35 °C (gold). The first four diffraction patterns are consistent with the reported pattern for Cu^{I}-MFU-4*l* in reference 2.

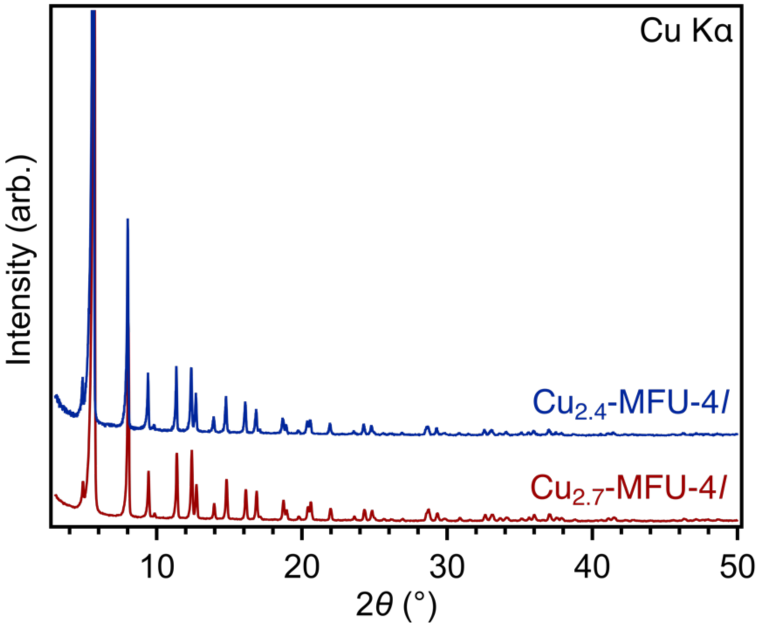

Figure S39. Powder x-ray diffraction patterns (Cu Kα) collected under air for $Cu_{2.7}$-MFU-4*l* and for $Cu_{2.4}$-MFU-4*l*.

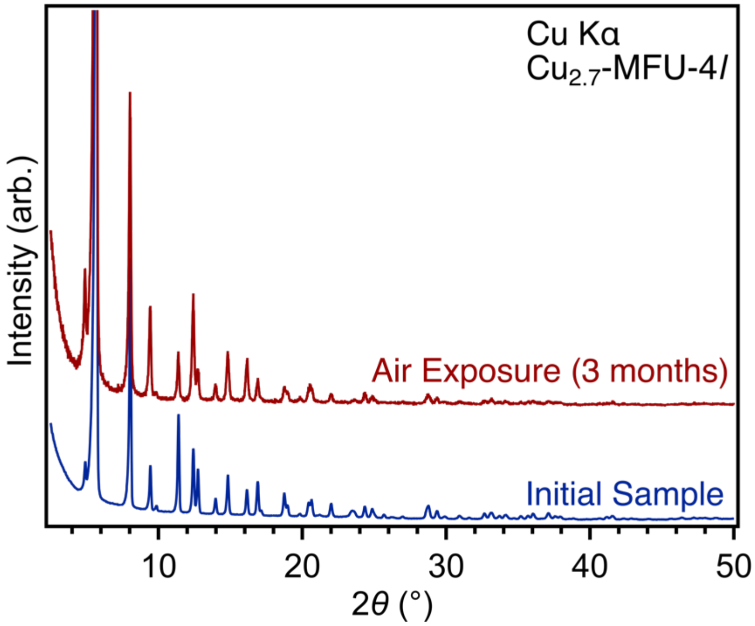

Figure S40. Powder x-ray diffraction patterns (Cu Kα) collected under air for $Cu_{2.7}$-MFU-4*l* before (blue trace) and after (red trace) exposure to air for three months in an open 20 mL vial in a cupboard with exclusion of light. Relative humidity levels were observed to range from 30 to 50% based on a proximal LI-COR 850 humidity monitor. Changes in peak asymmetry upon air exposure are attributed to the presence of water in the pores.

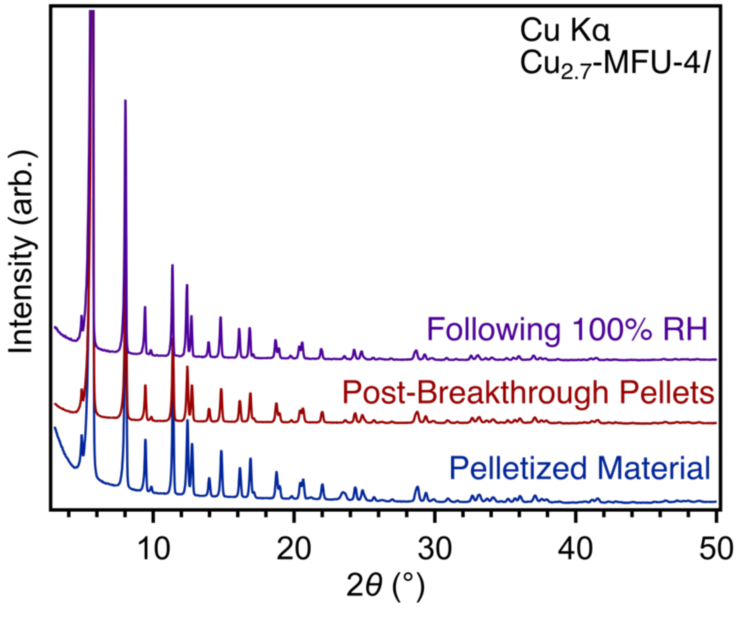

Figure S41. Powder x-ray diffraction patterns (Cu Kα) collected in air for pelletized $Cu_{2.7}$-MFU-4*l* (blue), $Cu_{2.7}$-MFU-4*l* following completion of breakthrough analysis and subsequent regeneration under flowing He at 150 °C (red), and $Cu_{2.7}$-MFU-4*l* following exposure to 100% relative humidity in air for 30 minutes and reactivation under dynamic vacuum at 150 °C. No substantial changes in the crystallinity of the material were apparent after humidity exposure.

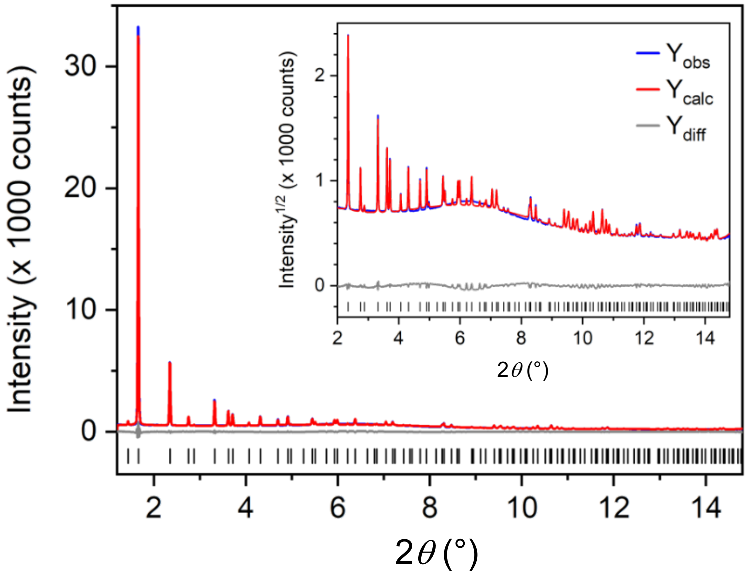

Figure S42. Pawley fit with synchrotron powder x-ray diffraction pattern of activated $Cu_{2.4}$-MFU-4*l* at 195 K from 1.2° to 14.8° and from 2° to 14.8° (inset). Blue and red lines represent the observed and calculated diffraction patterns, respectively. The gray line represents the difference between observed and calculated pattern. The black tick marks indicate calculated Bragg peak positions. Space group *F*m–3m, a = 31.2090(14) Å, V = 30397.7(2) Å^3. Figures-of-merit (as defined by TOPAS): R_{wp} = 4.99%, R_p = 3.80%, R_{exp} = 4.24%. The wavelength was 0.45192 Å.

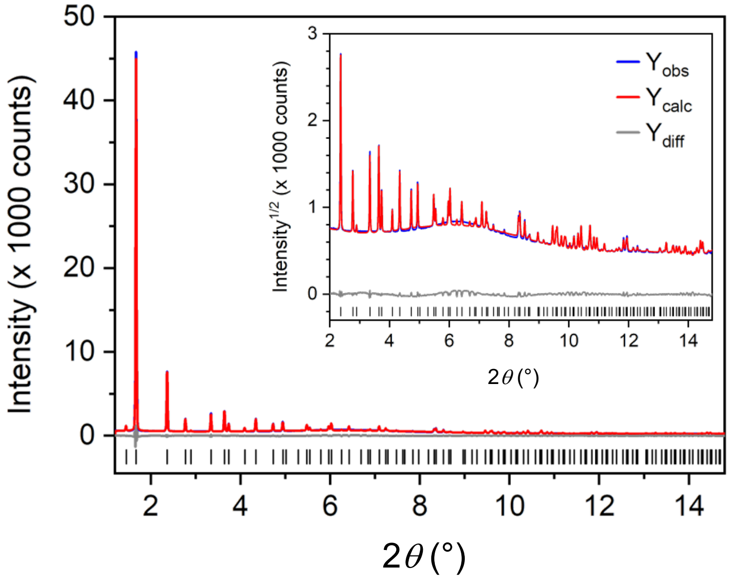

Figure S43. Pawley fit with synchrotron powder x-ray diffraction pattern of activated $Cu_{2.4}$-MFU-4*l* dosed with 8 mbar O_2 at 195 K from 1.2° to 14.8° and from 2° to 14.8° (inset). Blue and red lines represent the observed and calculated diffraction patterns, respectively. The gray line represents the difference between observed and calculated pattern. The black tick marks indicate calculated Bragg peak positions. Space group *F*m–3m, a = 31.0044(3) Å, V = 29803.7(8) Å^3. Figures-of-merit (as defined by TOPAS): R_{wp} = 4.17%, R_p = 3.11%, R_{exp} = 3.97%. The wavelength was 0.45192 Å.

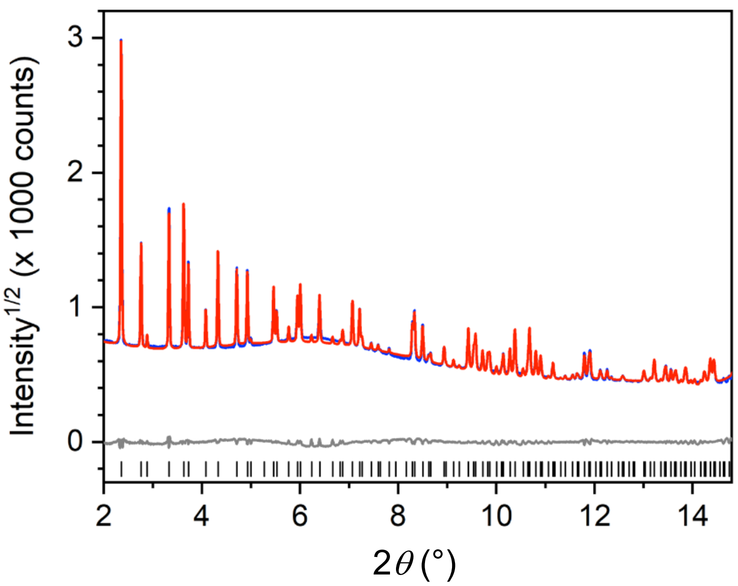

Figure S44. Pawley fit with synchrotron powder x-ray diffraction pattern of activated $Cu_{2.4}$-MFU-4*l* dosed with 9 mbar N_2 at 195 K from 1.2° to 14.8° and from 2° to 14.8° (inset). Blue and red lines represent the observed and calculated diffraction patterns, respectively. The gray line represents the difference between observed and calculated pattern. The black tick marks indicate calculated Bragg peak positions. Space group *F*m–3m, a = 31.0997(11) Å, V = 30079(3) Å^3. Figures-of-merit (as defined by TOPAS): R_{wp} = 3.77%, R_p = 2.76%, R_{exp} = 4.01%. The wavelength was 0.45192 Å.

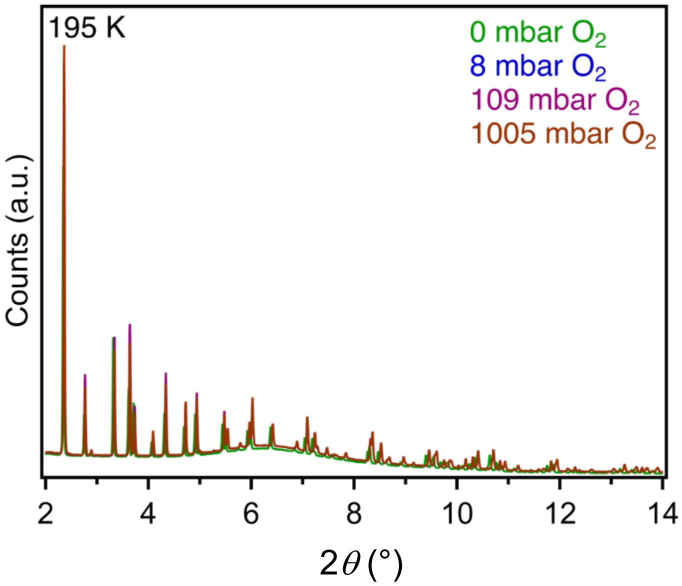

Figure S45. Synchrotron powder x-ray diffraction patterns (λ = 0.45192 Å, 195 K) of activated $Cu_{2.4}$-MFU-4*l* (green) dosed with variable quantities of O_2 at 8 mbar (blue), 109 mbar (purple), and 1005 mbar (dark orange).

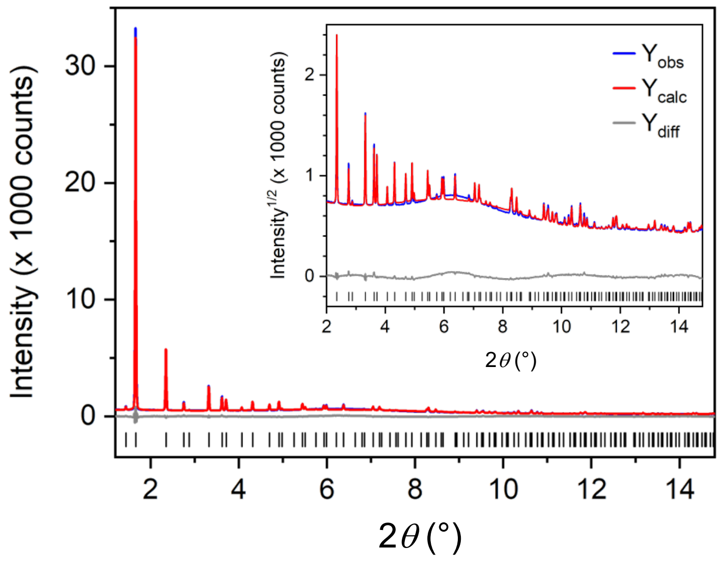

Figure S46. Rietveld refinement with synchrotron powder x-ray diffraction data for activated $Cu_{2.4}$-MFU-4*l* collected under vacuum at 195 K from 1.2° to 14.82°. Blue and red lines represent the observed and calculated patterns, respectively. The gray line represents the difference between the observed pattern and the calculated pattern. The black tick marks indicated calculated Bragg peak positions. The inset pattern shows a magnified view of the high angle region with square root of intensity as y-axis. Figures of merit (as defined by TOPAS): R_{wp} = 4.91%, R_p = 4.00%, R_{exp} = 4.39%, R_{Bragg} = 1.26%. GoF = 1.12 (λ = 0.45192 Å).

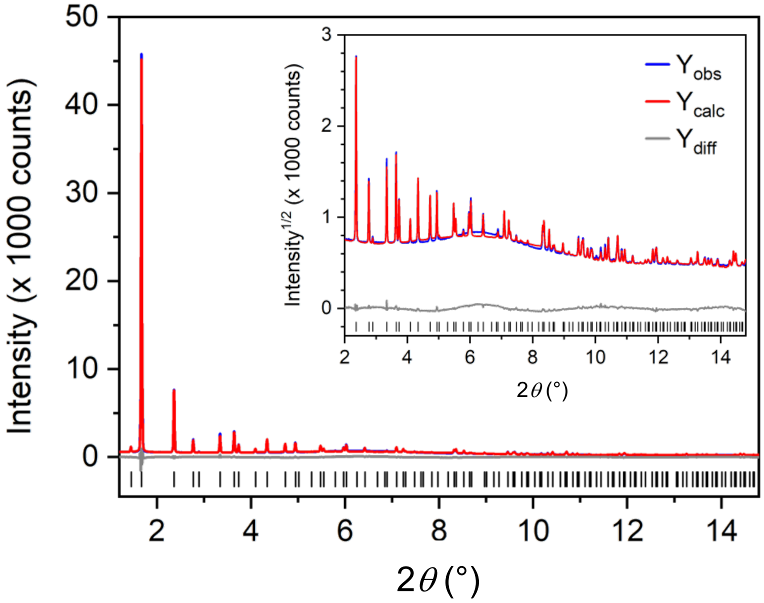

Figure S47. Rietveld refinement with synchrotron powder X-ray diffraction data for O_2-dosed Cu^{I}-MFU-4*l* collected at 195 K at 8 mbar from 1.2° to 14.82°. Blue and red lines represent the observed and calculated patterns, respectively. The gray line represents the difference between the observed pattern and the calculated pattern. The black tick marks indicated calculated Bragg peak positions. The inset pattern shows a magnified view of the high angle region with square root of intensity as y-axis. Figures of merit (as defined by TOPAS): R_{wp} = 5.06%, R_p = 4.00%, R_{exp} = 4.10%, R_{Bragg} = 1.40%. GoF = 1.23 (λ = 0.45192 Å).

Table S10. Experimental conditions, unit cell parameters, and figures-of-merit as determined by performing a structureless Pawley refinement of powder X-ray diffraction patterns for $Cu_{2.4}$-MFU-4*l*, 9 mbar N_2-dosed $Cu_{2.4}$-MFU-4*l*, and 8 mbar O_2-dosed $Cu_{2.4}$-MFU-4*l*.

	**$Cu_{2.4}$-MFU-4*l***	**N_2-dosed $Cu_{2.4}$-MFU-4*l***	**O_2-dosed $Cu_{2.4}$-MFU-4*l***
λ (Å)	0.45192	0.45192	0.45192
Temperature (K)	195	195	195
Space Group	*F*m–3m	*F*m–3m	*F*m–3m
a (Å)	31.2090(14)	31.0997(11)	31.0044(3)
b (Å)	31.2090(14)	31.0997(11)	31.0044(3)
c (Å)	31.2090(14)	31.0997(11)	31.0044(3)
V (Å^3)	30397.7(2)	30079(3)	29803.7(8)
R_{wp} (%)	4.99	3.77	4.17
R_{exp} (%)	4.24	4.01	3.97
R_p (%)	3.80	2.76	3.11

Table S11. Unit cell parameters and figures of merit obtained by Rietveld refinement using synchrotron X-ray powder diffraction patterns of Cu^I-MFU-4*l* under dynamic vacuum and dosed with 8 mbar O_2 gas.

	**$Cu_{2.4}$-MFU-4*l***	**O_2-dosed $Cu_{2.4}$-MFU-4*l***
λ (Å)	0.45192	0.45192
Temperature (K)	195	195
Space Group	*F*m–3m	*F*m–3m
a (Å)	30.2086(10)	31.0045(8)
b (Å)	30.2086(10)	31.0045(8)
c (Å)	30.2086(10)	31.0045(8)
V (Å^3)	30396(3)	29803(2)
R_{wp} (%)	4.91	5.06
R_{exp} (%)	4.39	4.10
R_p (%)	4.00	4.00
R_{Bragg} (%)	1.26	1.40
GoF	1.12	1.23

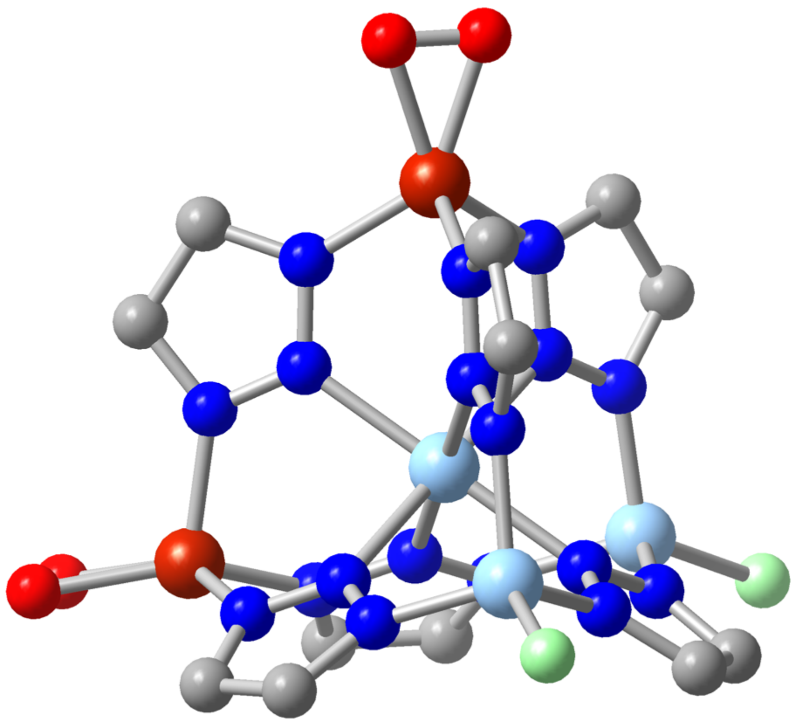

Figure S48. Solid-state structure obtained from Rietveld refinement of $Cu_{2.4}$-MFU-4*l* dosed with 8 mbar O_2 at 195 K. The linker is truncated for clarity, and structural disorder is omitted for clarity. Brown, light blue, dark blue, gray, green, and red spheres represent Cu, Zn, N, C, Cl and O atoms respectively. The Cu:Zn ratio was determined based on ICP-OES values, and all peripheral Zn sites were refined with terminal Cl ligands.

8. Magnetic Characterization Data

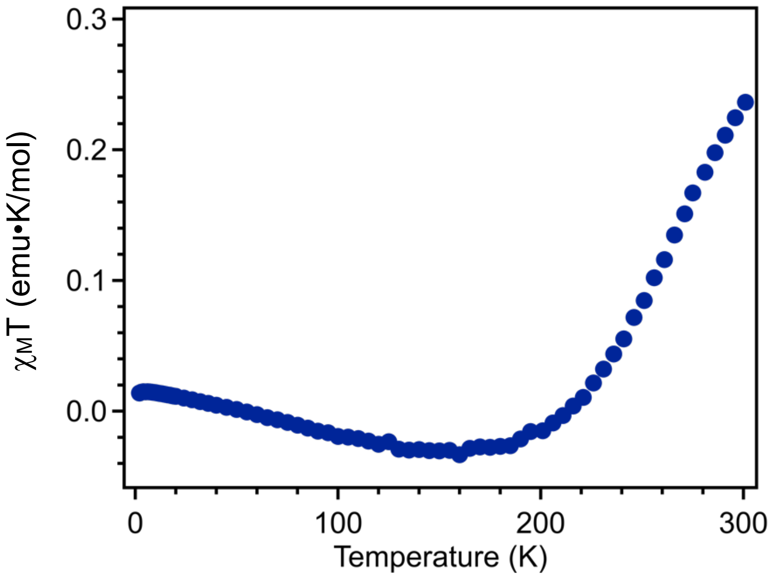

Figure S49. Plot of the molar magnetic susceptibility–temperature product ($\chi_M T$) versus T for O_2-dosed $Cu_{2.4}$-MFU-4*l*. Data were collected at 1 T. The magnitude of $\chi_M T$ increases with increasing temperature, consistent with an equilibrium between superoxide species bound side-on and end-on to copper(II), as discussed in the main text.

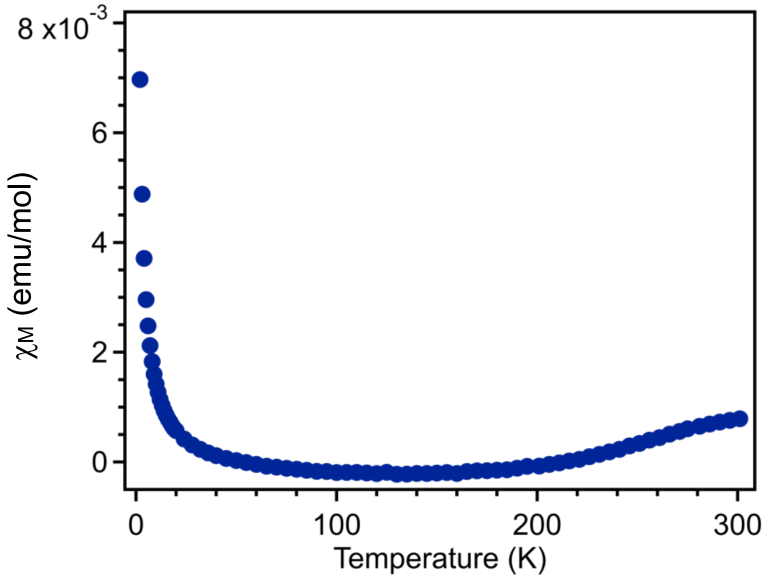

Figure S50. Plot of χ_M versus T for O_2-dosed $Cu_{2.4}$-MFU-4*l*, illustrating the low values of χ at temperatures between 100 and 200 K.

9. Breakthrough Measurements.

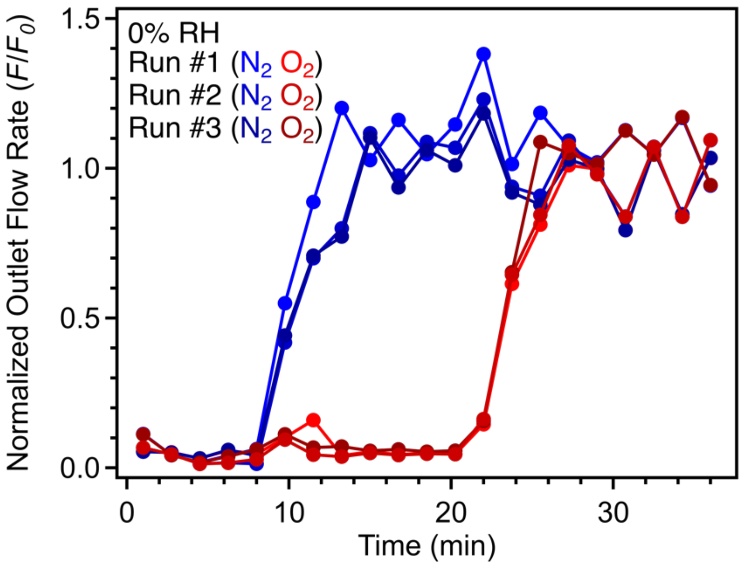

Figure S51. Multicomponent breakthrough data of compressed air with $Cu_{2.7}$-MFU-4*l* at 25 °C and 0% relative humidity. Regeneration was conducted at 150 °C for a minimum of 1 h prior to repeat measurements.

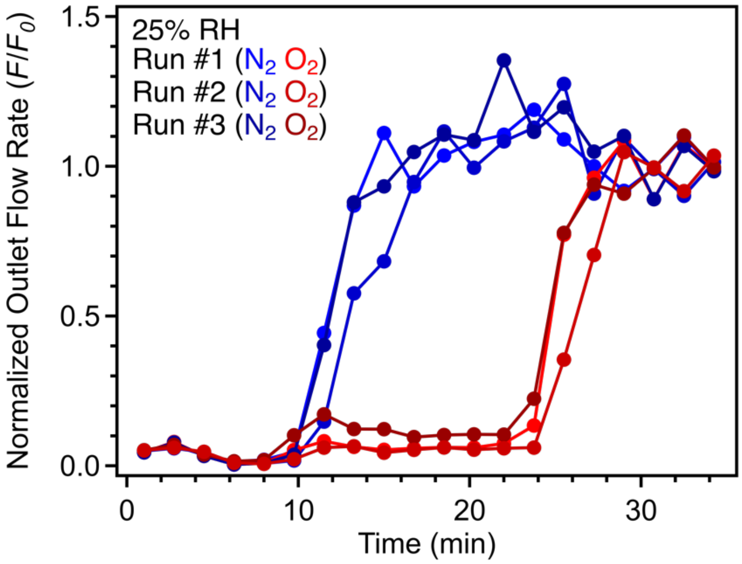

Figure S52. Multicomponent breakthrough data collected for $Cu_{2.7}$-MFU-4*l* exposed to a compressed air stream at 25 °C and 25% relative humidity. Regeneration was conducted at 150 °C for a minimum of 1 h prior to repeat measurements.

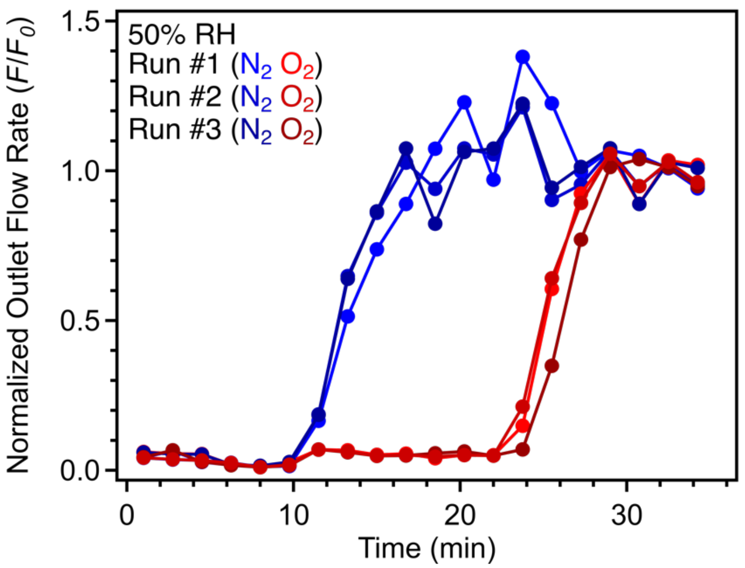

Figure S53. Multicomponent breakthrough data collected for $Cu_{2.7}$-MFU-4*l* exposed to a compressed air stream at 25 °C and 50% relative humidity. Regeneration was conducted at 150 °C for a minimum of 1 h prior to repeat measurements.

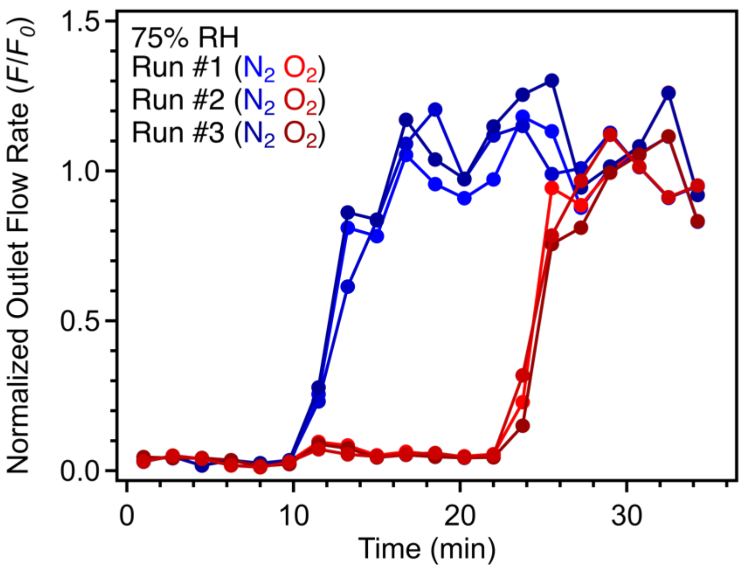

Figure S54. Multicomponent breakthrough data collected for $Cu_{2.7}$-MFU-4*l* exposed to a compressed air stream at 25 °C and 75% relative humidity. Regeneration was conducted at 150 °C for a minimum of 1 h prior to repeat measurements.

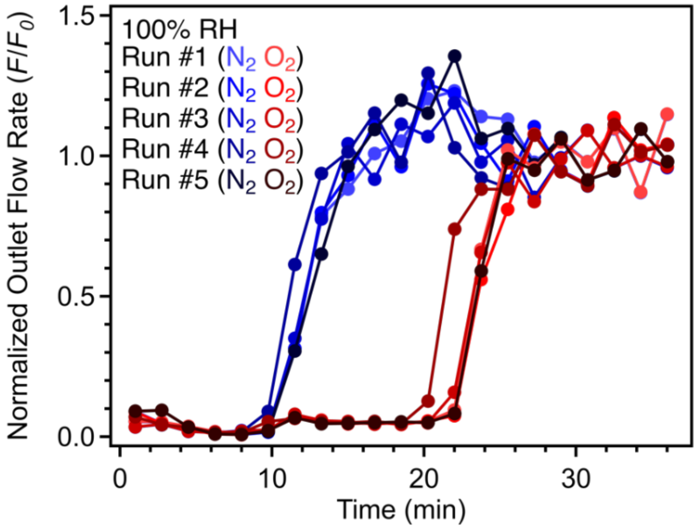

Figure S55. Multicomponent breakthrough data collected for $Cu_{2.7}$-MFU-4*l* exposed to a compressed air stream at 25 °C and 100% relative humidity. Regeneration after the first two run in each case was conducted at 150 °C for a minimum of 1 h prior to repeat measurements. After the third and four runs in each case, the material was regenerated by heating under flowing He at 50 °C for 24 h.

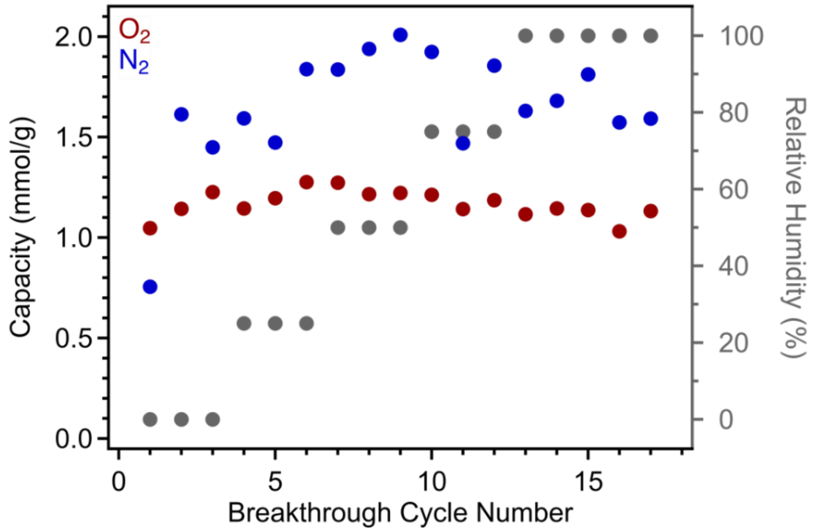

Figure S56. Compiled data obtained from multicomponent breakthrough "cycling" experiments performed with $Cu_{2.7}$-MFU-4*l* (Figures S51–S55). All measurements were carried out on the same batch of material. For the first fifteen measurements, regeneration was conducted at 150 °C under a flow of He (10 sccm) for a minimum of 1 h to ensure sample reactivation. Heating the sample to temperatures above 100 °C is necessary to achieve complete water desorption, as monitored by a nondispersive infrared sensor. For the last two data points, regeneration was carried out under a flow of He gas at 50 °C, resulting in the retention of water within the framework. From these latter data, even when water remains in the framework, the material exhibits O_2 and N_2 capacities on subsequent adsorption runs that are comparable to those measured after full desorption of water from the material.

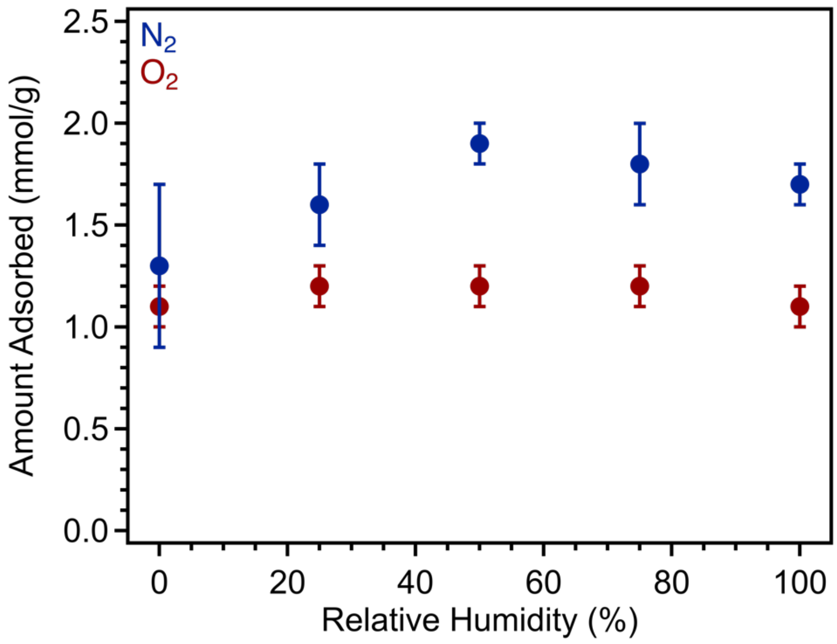

Figure S57. Experimental O_2 (red points) and N_2 (blue points) capacities determined for $Cu_{2.7}$-MFU-4*l* based on dry and humid compressed air breakthrough experiments. The error bars depict the first standard deviation, and the data point are average capacities over three separate runs (see Figure S56 and Table S12).

Table S12. Oxygen and N_2 capacities (in mmol/g) determined for a sample of $Cu_{2.7}$-MFU-4*l* over the course of continuous breakthrough experiments under dry and humid air (see Figure S56). Numbers in parentheses represent standard deviations from the three independent runs at each RH level. Capacities determined from the final two breakthrough runs under 100% RH (see Figure S55), wherein the sample was only partially regenerated, are consistent with the capacities determined from the first three runs at 100% RH (1.0 then 1.1 mmol/g for O_2 and 1.6 then 1.6 mmol/g for N_2).

**Gas**	**Run**	**Relative Humidity Level**				
		**0%**	**25%**	**50%**	**75%**	**100%**
	1	1.1	1.2	1.3	1.2	1.1
O_2	2	1.1	1.2	1.2	1.1	1.2
	3	1.2	1.3	1.2	1.2	1.1
**Average O_2**		**1.1(1)**	**1.2(1)**	**1.2(1)**	**1.2(1)**	**1.1(1)**
	1	0.8	1.6	1.8	1.9	1.6
N_2	2	1.6	1.5	1.9	1.5	1.7
	3	1.5	1.8	2.0	1.9	1.8
**Average N_2**		**1.3(4)**	**1.6(2)**	**1.9(1)**	**1.8(2)**	**1.7(1)**

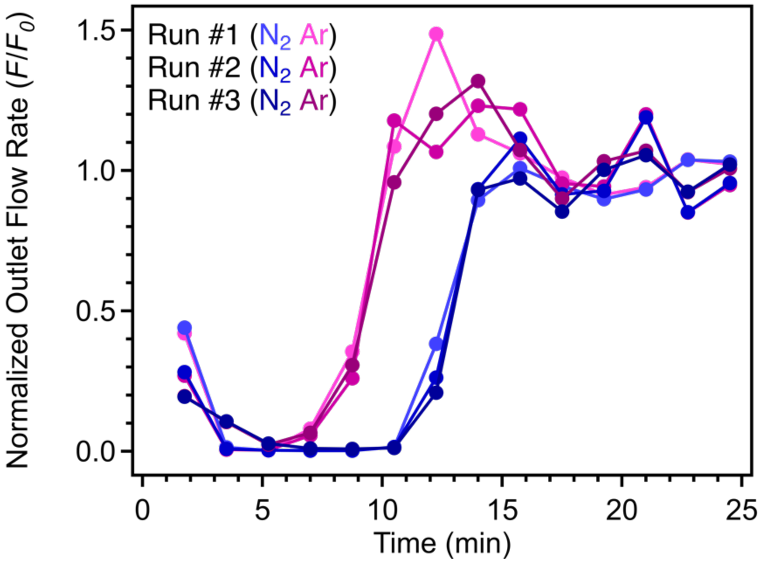

Figure S58. Multicomponent breakthrough data for $Cu_{2.7}$-MFU-4*l* exposed to an equal component mixture of N_2 and Ar at 1 bar and 25 °C. The N_2/Ar stream was allowed to equilibrate prior to measurements to ensure a well-mixed stream (see Section 1.13). Three total runs were completed, and between each run, regeneration was conducted at 25 °C under flow of He (10 sccm) for a minimum of 10 min. Reactivation of the material was confirmed once Ar and N_2 were no longer detected by the gas chromatograph. Breakthrough of Ar occurred first during each measurement, consistent with the larger binding enthalpy for N_2 and a greater selectivity for N_2 over Ar. The measured capacity for N_2 is higher than that for Ar (0.9 versus 0.2 mmol/g). Based on these data, $Cu_{2.7}$-MFU-4*l* may also be of interest for the removal of N_2 and O_2 impurities from Ar streams under mild conditions. The detection of N_2 and Ar at early timepoints is ascribed to the presence of residual gas in the tubing connecting the breakthrough column to the GC (see Figure S62 and Section 1.13).

Table S13. Measurement N_2/Ar breakthrough capacities for $Cu_{2.7}$-MFU-4*l*.

Run	N_2 Capacity (mmol/g)	Ar Capacity (mmol/g)
Run #1	0.9	0.2
Run #2	0.8	0.1
Run #3	0.9	0.2
Average	0.9(1)	0.2(1)

Note: the lower N_2 capacities measured under these conditions relative to the capacities from the compressed air breakthrough experiments me may be due to the lower partial pressure of N_2 in the Ar/N_2 stream (50% N_2) relative to the compressed air stream (79% N_2).

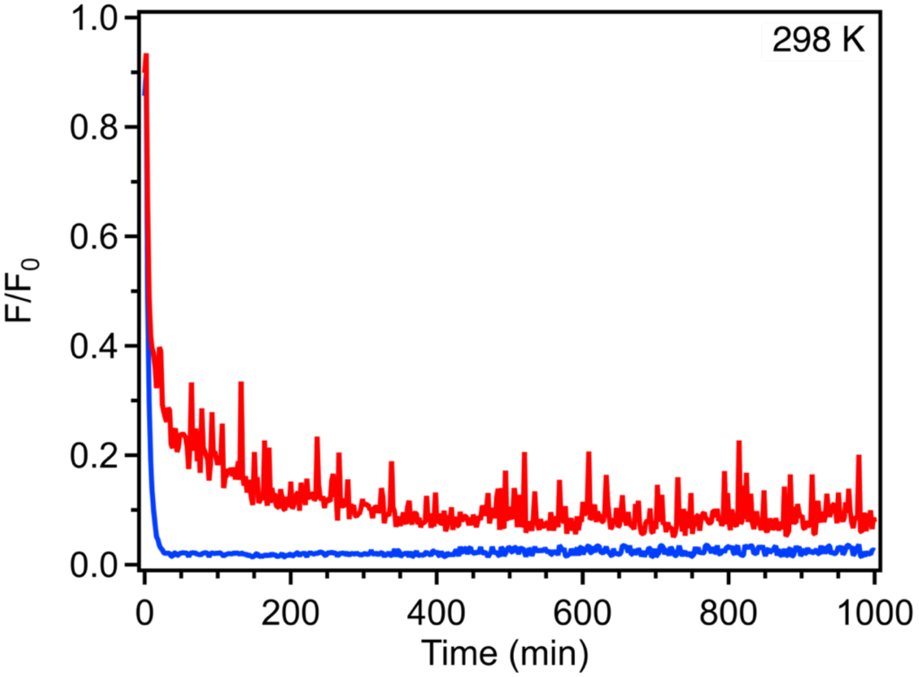

Figure S59. Desorption data collected at 25 °C following treatment of $Cu_{2.7}$-MFU-4*l* with compressed air at 50% relative humidity, revealing a more gradual desorption of O_2 (red) compared to N_2 (blue). Thus, differences in O_2 and N_2 desorption kinetics can also in principle be harnessed at ambient temperatures to obtain enhanced purity O_2 from air.

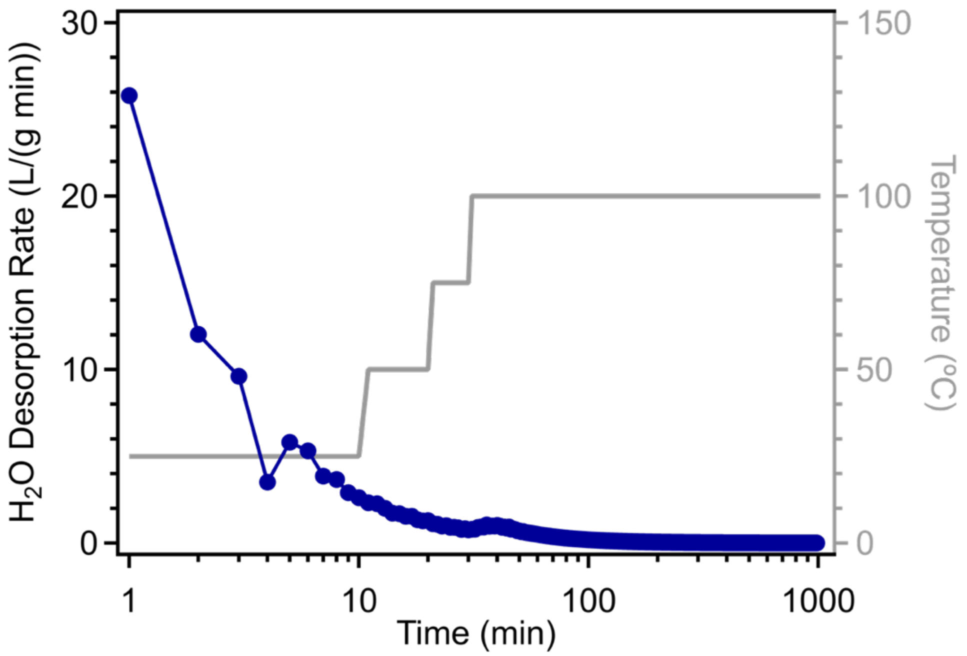

Figure S60. Time-dependent water desorption data collected for $Cu_{2.7}$-MFU-4*l* following adsorption run #9 (see Figure S56) using compressed air at 50% relative humidity. Water was desorbed from the material under flowing He (10 sccm) with concurrent removal of adsorbed O_2 and N_2 while ramping the temperature from 25 to 100 °C. The water desorption rate was measured using a non-dispersive infrared (NDIR) sensor at the outlet stream, and integration of the curve above yielded a total capacity of 2.82 mmol/g, which was estimated to have been adsorbed during run #9.

We note that between cycles, the amount of water that was quantified by the NDIR sensor was observed to vary substantially, attributed to water condensing in the piping of the breakthrough setup. As a result, we present this data qualitatively to demonstrate that (i) $Cu_{2.7}$-MFU-4*l* adsorbs water under breakthrough measurements and that (ii) heating to 100 °C under He flow is necessary under these conditions to fully remove bound water.

10. Additional Characterization

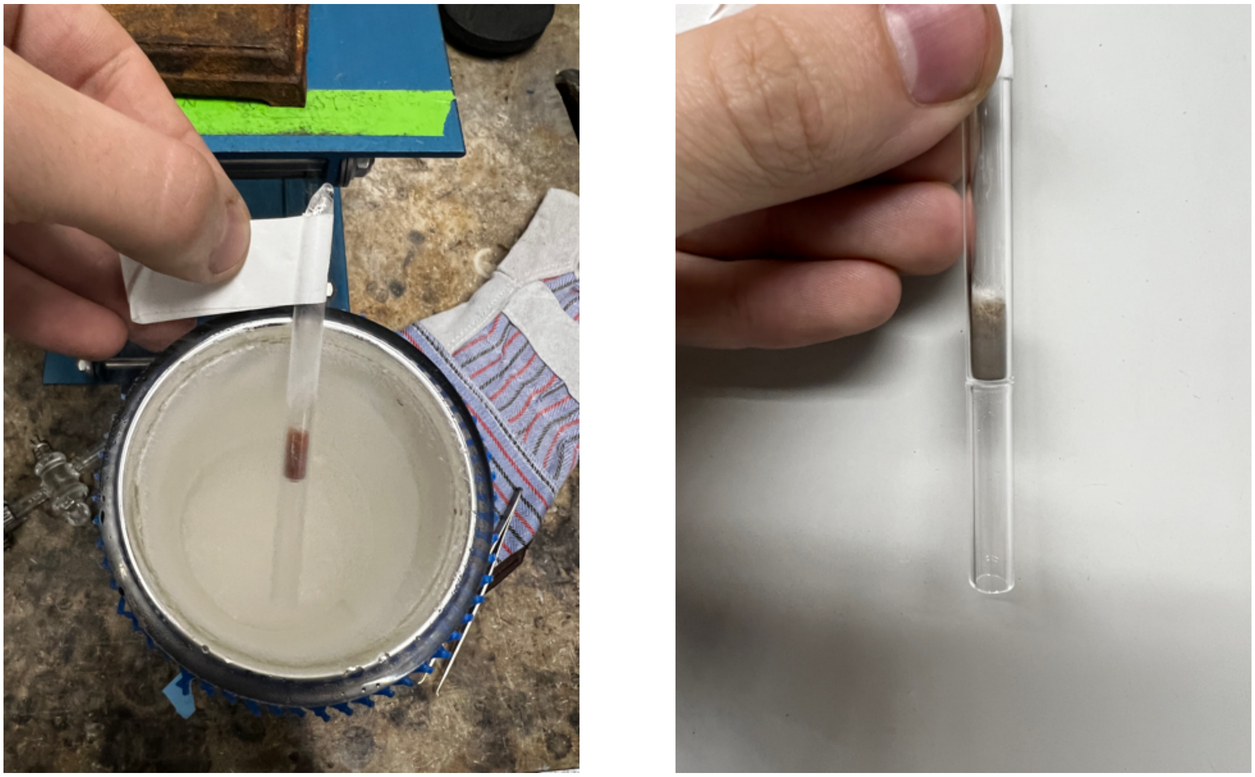

Figure S61. Visual color change for O_2-dosed Cu^{I}-MFU-4*l* upon immediate removal from a 77 K liquid nitrogen bath (left) compared to the same material upon standing at room temperature for 5 minutes (right). A visual color change from rose-pink to gray-brown is evident, attributed to changes in the relative populations of the O_2-bound geometries.

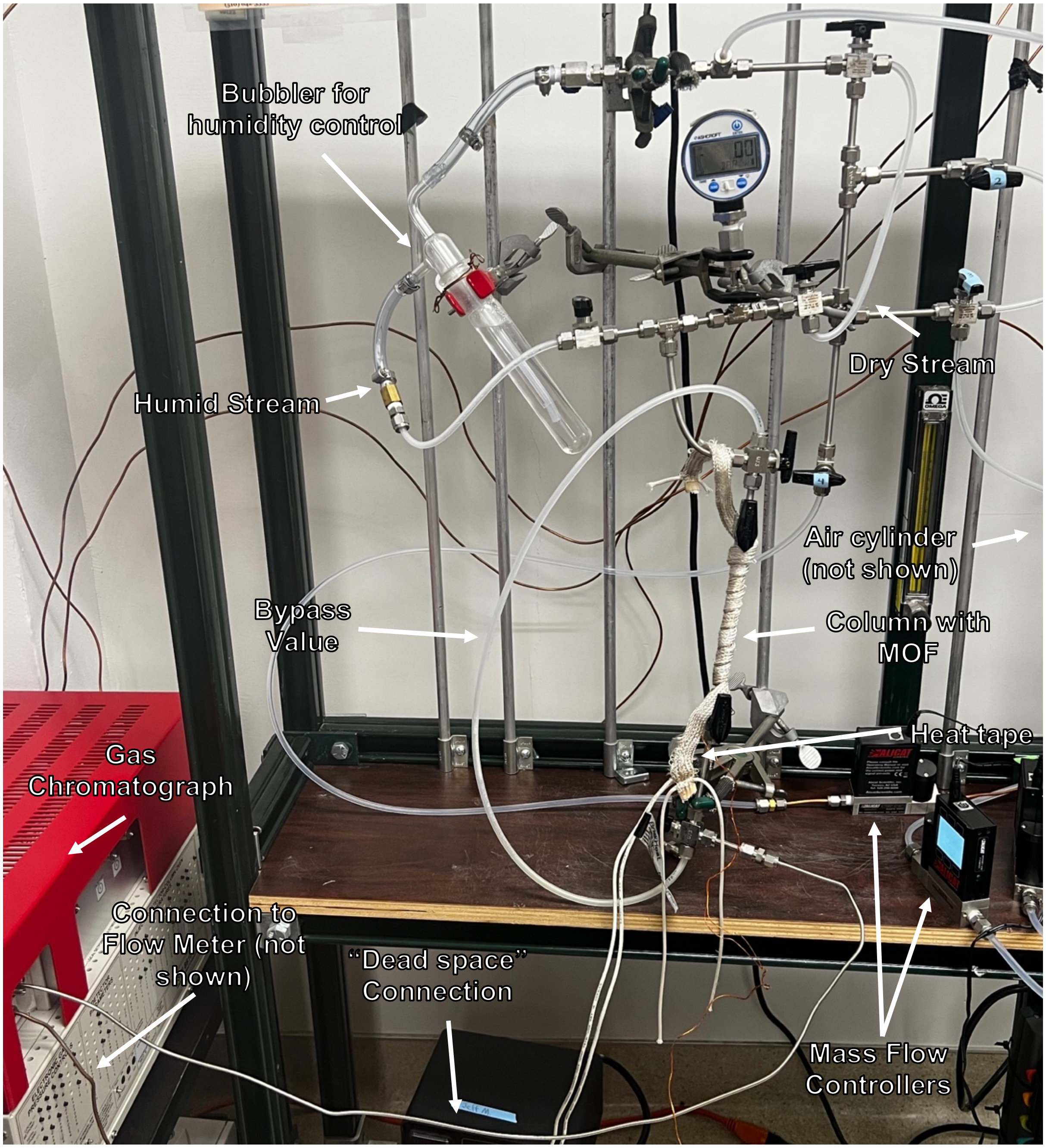

Figure S62. Custom-built multicomponent breakthrough apparatus with attached gas chromatograph. In brief, dry and humidified streams of air are premixed to afford the desired humidity level (for instance, air at 25% RH was obtained from a 3:1 mixture of dry air and humidified air). This mixed stream is then sent through the bypass value (while the column containing the MOF is kept closed) prior to the start of the measurement, to enable equilibration of the stream. The bypass valve is closed at the immediate start of the measurement as the column with the MOF is open. The "dead space" connection contains residual air from this equilibration period, which likely is the cause of the trace amounts of O_2 and N_2 detected at the start of the breakthrough measurements (see for example Figure 6a in the main text). For N_2/Ar breakthrough measurements, the water bubbler was removed from the inlet stream, and the mixed gas stream was produced by combining equal amounts of gas streams obtained from separate cylinders of N_2 and Ar.

Table S14. Gravimetric quantification of Cu(I) sites in $Cu_{2.7}$-MFU-4*l* and $Cu_{2.4}$-MFU-4*l*.

Framework	Initial Sample Mass (g)	CO-Dosed Sample Mass (g)	Mass Increase (wt%)	Cu(I) Loading per Node	Cu(I) Loading from H_2 Adsorption Data
$Cu_{2.7}$-MFU-4*l*	0.1761	0.1867	6.0	2.4(1)	2.4
	0.1762	0.1866	5.9		
	0.1762	0.1866	5.9		
$Cu_{2.4}$-MFU-4*l*	0.1234	0.1305	5.8	2.4(1)	2.4
	0.1234	0.1304	5.7		
	0.1234	0.1305	5.8		

The mass increase following dosing $Cu_{2.7}$-MFU-4*l* and $Cu_{2.4}$-MFU-4*l* with CO and subsequent evacuation at 25 °C for 12 h is ascribed to chemisorbed CO at Cu(I) sites. We find are mass increases are consistent with the expected Cu(I) loading in both frameworks as ascertained by H_2 isotherm analysis at 77 K.

11. Computational Details

11.1. Geometry Optimization. Density functional theory (DFT) calculations were conducted using the model pentanuclear cluster $CuZn_4Cl_3(bta)_3(ta)_3$ (bta^- = 1,2,3-benzenetriazolate; ta^- = 1,2,3-triazolate) and O_2, N_2, and water as adsorbates (see reference 2 for details of the cluster model and computational methods used). Accurate simulation of small-molecule binding at the Cu^I sites requires an adroit handling of metal–ligand interactions that occur at short range as well as long-range dispersion interactions. Calculations were performed using the BP86/RI-def2-SV(P) level of theory. The BP86 functional was selected based on its literature precedence for modelling trispyrazoleborate copper–dioxygen complexes.[29] Geometries were converged to 3×10^{-4} kJ/mol in energy, and 3×10^{-5} a.u. in the maximum gradient component. Spin densities for the open-shell singlet configuration upon O_2 binding were determined using a range-separated hybrid functional (ωB97M-V), noting that pure functional prefer closed-shell singlet solutions. All simulations were executed using QChem version 5.4. Preliminary test calculations were conducted on a pre-release version of QChem 6.1.[30]

Table S15. Calculated electronic energy (ΔE) for binding of H_2O, N_2, and O_2 to a single Cu^I site in the model complex $CuZn_4Cl_3(bta)_3(ta)_3$.

Adsorbate	Electronic Energy (ΔE, kJ/mol)
O_2 (S = 3), end-on binding	−64.6
O_2 (S = 1), side-on binding	−45.8
N_2	−31.6
H_2O	−27.0

11.2. Partial Charges and Spins. For the final converged geometries obtained for O_2 bound side-on (singlet state) and end-on (triplet state) to the model cluster, we calculated the partial charges for both the Cu site and for the adsorbate (this was also done for N_2 and H_2O). Singlet and triplet states were modeled within an unrestricted Kohn-Sham formalism. The results are shown in Table S16, along with results for N_2 and H_2O for comparison. Binding of O_2 either an end-on or side-on fashion results in an increase in the partial unpaired spin on Cu consistent with partial Cu oxidation to Cu(II). This marginal increase in partial charge on Cu is not found upon binding of N_2 or H_2O. There is also partial accumulation of unpaired spin on bound O_2 in the triplet configuration, which, in tandem with the DRIFTS data, is consistent with superoxide character. For the side-on bound O_2 species, calculations indicate an open-shell singlet to be more favorable than a closed-shell singlet.

Table S16. Partial charges and spins calculated for the model cluster $CuZn_4Cl_3(bta)_3(ta)_3$ and for the cluster with O_2, N_2, and H_2O bound at the copper site.

	Spin State	Cu		Adsorbate	
		**Charge (a.u.)**	**Spin (a.u.)**	**Charge (a.u.)**	**Spin (a.u.)**
$CuZn_4Cl_3(bta)_3(ta)_3$	$2S + 1 = 1$	0.35	0.00	–	–
$CuZn_4Cl_3(bta)_3(ta)_3 + O_2$ (side-on)*	$2S + 1 = 1$	0.57	0.53	-0.30	-0.62
$CuZn_4Cl_3(bta)_3(ta)_3 + O_2$ (end-on)	$2S + 1 = 3$	0.42	0.34	-0.16	1.61
$CuZn_4Cl_3(bta)_3(ta)_3 + N_2$	$2S + 1 = 1$	0.33	0.00	0.00	0.00
$CuZn_4Cl_3(bta)_3(ta)_3 + H_2O$	$2S + 1 = 1$	0.33	0.00	0.00	0.00

*The respective spin densities on the three nitrogen atoms in the primary coordination sphere of the Cu ion are 0.05, 0.04, and 0.01.

11.3. Coordinates.

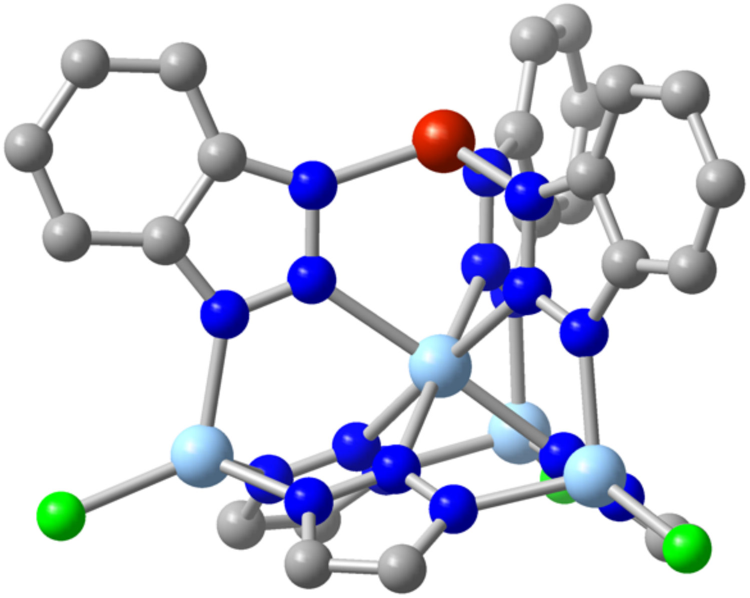

Cu^I-MFU-4*l* Cluster Model for O_2 Binding.

N	2.55512	0.80674	2.02845
C	3.07388	1.79302	2.80234
N	-0.47883	-2.63807	2.01286
C	0.14868	-3.58747	2.75148
N	-1.94458	1.70957	2.13117
C	-3.04373	1.62853	2.92226
N	1.07469	2.39770	2.08469
C	2.12231	2.81534	2.83867
N	1.35040	1.18563	1.60219
N	-0.45931	1.86797	-2.23387
C	-0.76238	3.15177	-2.61112
C	-0.92002	3.75549	-3.88193
C	-1.22996	5.11555	-3.89751
H	-0.80092	3.17285	-4.80992
N	2.96523	-0.90003	-0.52399
C	3.77529	-1.13739	-1.60148
C	5.12740	-1.55083	-1.69174
C	5.64665	-1.69435	-2.97812
H	5.71802	-1.74230	-0.78084
N	-2.29369	-2.10332	-0.44545
C	-2.96485	-2.66465	-1.49772
C	-4.01749	-3.61164	-1.54624
C	-4.47351	-3.96651	-2.81538
H	-4.43837	-4.03189	-0.61808

N	1.74438	-0.50456	-2.32336
C	2.98770	-0.88166	-2.76340
N	1.76708	-0.52572	-0.98736
C	3.52869	-1.03293	-4.06306
C	4.86035	-1.43962	-4.14579
H	2.92093	-0.83698	-4.96142
N	1.64008	-2.15085	1.98330
C	1.51012	-3.27435	2.73244
N	0.43310	-1.77966	1.55527
Zn	3.28739	-1.03092	1.47861
Cu	-0.05826	0.05553	-2.89764
N	-1.42297	-1.23004	-2.27936
C	-2.40270	-2.10211	-2.68236
N	-1.38650	-1.25566	-0.94360
C	-2.87565	-2.47360	-3.96411
C	-3.91167	-3.40682	-4.00599
H	-2.44235	-2.04129	-4.88064
Zn	-2.46165	-2.34929	1.56168
N	-2.58357	-0.36859	2.09917
C	-3.45434	0.29329	2.90174
N	-1.68002	0.49778	1.64105
Zn	-0.72332	3.28670	1.64102
Cl	-1.12857	5.23857	2.54316
Zn	0.00787	-0.00608	0.29930
N	-0.70009	3.03954	-0.37529
C	-0.91685	3.90769	-1.41097
N	-0.43047	1.83800	-0.89809
C	-1.23187	5.28887	-1.43289
C	-1.38272	5.86981	-2.69166
H	-1.34604	5.85382	-0.49320
H	-1.36217	5.62973	-4.86478
H	6.69538	-2.01272	-3.10499
H	-5.29253	-4.69974	-2.90964
H	5.32593	-1.57112	-5.13745
H	-4.31302	-3.72657	-4.98276
H	-1.62720	6.94313	-2.76570
Cl	5.22800	-1.65501	2.27230
Cl	-3.93065	-3.72409	2.42053
H	2.38278	-3.75744	3.18976
H	-0.41177	-4.40090	3.22918
H	2.11046	3.79245	3.33771
H	-3.44437	2.51294	3.43338
H	-4.28641	-0.22838	3.39127
H	4.06447	1.69372	3.26373

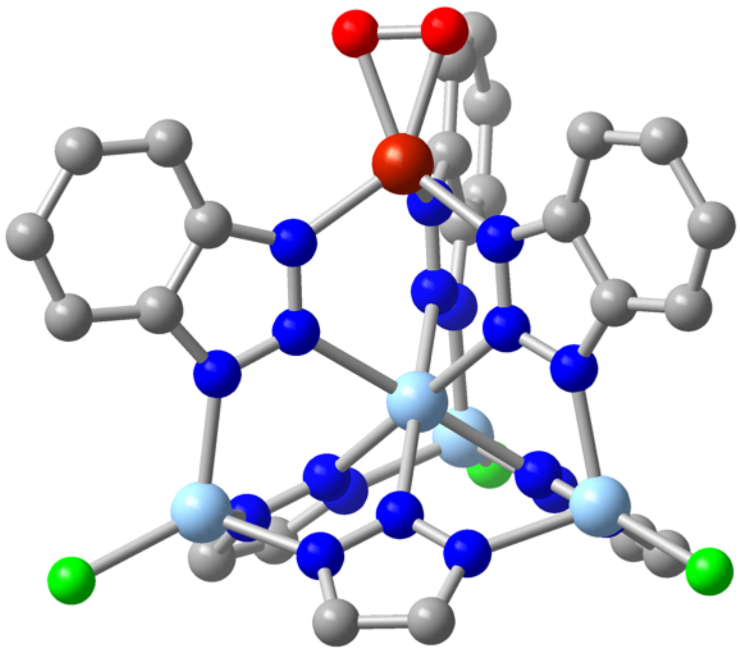

Side–on (η^2–O_2) O_2 with Cu^IZn-MFU-4*l* Cluster Model.

N	2.99916	0.91123	1.42187
C	3.68322	1.92371	2.01223
N	0.19111	-2.58735	2.32850
C	1.02529	-3.49564	2.89344
N	-1.33250	1.72966	2.73162
C	-2.17513	1.64888	3.79211
N	1.54777	2.46813	1.86366
C	2.75025	2.92426	2.29600
N	1.71369	1.25436	1.33829
N	-0.99096	1.75213	-1.89950
C	-1.37510	3.02530	-2.24168
C	-1.81754	3.59386	-3.46045
C	-2.11781	4.95609	-3.43850
H	-1.91723	2.98413	-4.37212
N	2.68857	-0.82664	-1.12269
C	3.18041	-1.04211	-2.38379
C	4.46508	-1.42480	-2.84059
C	4.61571	-1.55371	-4.22080
H	5.28472	-1.60610	-2.12621
N	-2.19038	-2.14700	0.40622
C	-3.06916	-2.77820	-0.43246
C	-4.03024	-3.79246	-0.20030
C	-4.76268	-4.21429	-1.30953
H	-4.17459	-4.21299	0.80823
N	1.02298	-0.45399	-2.50765
C	2.10137	-0.80055	-3.28421

N	1.40615	-0.48002	-1.23434
C	2.26771	-0.93303	-4.68394
C	3.53570	-1.31072	-5.12660
H	1.43431	-0.74618	-5.37904
N	2.20328	-2.06796	1.68790
C	2.31842	-3.16190	2.48222
N	0.91503	-1.73461	1.60411
Zn	3.59270	-0.92003	0.70709
Cu	-0.85479	0.05942	-2.93935
N	-1.86923	-1.27601	-1.59883
C	-2.86078	-2.21593	-1.72727
N	-1.49644	-1.26495	-0.32388
C	-3.61398	-2.65482	-2.84516
C	-4.55740	-3.65473	-2.60994
H	-3.45364	-2.22508	-3.84548
Zn	-1.85277	-2.35245	2.39810
N	-1.88777	-0.36359	2.92823
C	-2.53151	0.30360	3.91852
N	-1.17002	0.50851	2.22171
Zn	-0.32814	3.30119	1.88379
Cl	-0.58769	5.28938	2.75746
Zn	0.09339	-0.01330	0.49187
N	-0.80182	2.96586	-0.07707
C	-1.25030	3.81115	-1.05856
N	-0.65833	1.75312	-0.61250
C	-1.55734	5.19353	-1.04402
C	-1.98982	5.74269	-2.25085
H	-1.44892	5.78339	-0.11910
H	-2.46588	5.44513	-4.36412
H	5.59563	-1.85159	-4.63086
H	-5.52345	-5.00362	-1.18523
H	3.71501	-1.42830	-6.20883
H	-5.16624	-4.02973	-3.45022
H	-2.24118	6.81591	-2.29545
Cl	5.70554	-1.45913	0.87173
Cl	-3.00081	-3.73969	3.63735
H	3.29618	-3.61324	2.69204
H	0.64156	-4.29832	3.53561
H	2.84843	3.91051	2.76670
H	-2.45266	2.53997	4.36899
H	-3.18435	-0.22122	4.62716
H	4.76505	1.85701	2.18285
O	-1.24836	-0.69297	-4.77472
O	-2.10949	0.25207	-4.49728

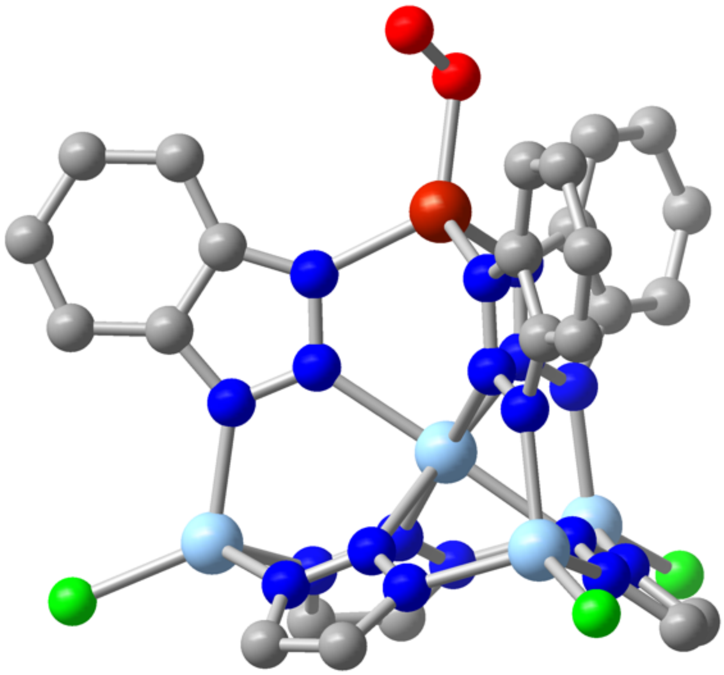

End–on (η^2–O_2) O_2 with Cu^IZn-MFU-4*l* Cluster Model.

N	3.01416	0.94399	1.41084
C	3.69700	1.94783	2.01638
N	0.19558	-2.55736	2.30937
C	1.02763	-3.46493	2.87908
N	-1.30986	1.74857	2.72678
C	-2.15092	1.66325	3.78778
N	1.56524	2.50208	1.85659
C	2.76559	2.94912	2.30292
N	1.73055	1.29313	1.31984
N	-1.06180	1.82394	-1.89616
C	-1.45904	3.10204	-2.19892
C	-1.95087	3.69016	-3.39059
C	-2.25892	5.04951	-3.33612
H	-2.07986	3.09845	-4.31028
N	2.71790	-0.82927	-1.11830
C	3.19998	-1.10871	-2.36944
C	4.47901	-1.52061	-2.81721
C	4.61685	-1.72185	-4.19021
H	5.30412	-1.67051	-2.10181
N	-2.18364	-2.13437	0.38289
C	-3.05002	-2.78240	-0.45694
C	-4.00693	-3.80015	-0.22295
C	-4.72460	-4.24043	-1.33483
H	-4.15823	-4.20873	0.78950
N	1.03978	-0.51895	-2.51351

C	2.11408	-0.90917	-3.27202
N	1.43320	-0.48508	-1.24143
C	2.26699	-1.12033	-4.66403
C	3.52904	-1.52542	-5.09855
H	1.42471	-0.97661	-5.35934
N	2.21081	-2.02921	1.68905
C	2.32296	-3.12536	2.48055
N	0.92285	-1.69883	1.59400
Zn	3.61171	-0.89060	0.70956
Cu	-0.88880	0.02602	-2.83442
N	-1.85046	-1.28934	-1.62573
C	-2.83398	-2.23675	-1.75664
N	-1.48674	-1.25746	-0.34502
C	-3.56855	-2.69459	-2.87796
C	-4.50830	-3.69764	-2.64073
H	-3.39372	-2.28066	-3.88367
Zn	-1.84364	-2.32759	2.38068
N	-1.88066	-0.34167	2.90162
C	-2.51759	0.31965	3.90030
N	-1.15711	0.53261	2.20253
Zn	-0.31022	3.33258	1.89253
Cl	-0.56817	5.29637	2.82246
Zn	0.11620	0.03927	0.46297
N	-0.80921	3.01588	-0.05829
C	-1.29485	3.87252	-1.00994
N	-0.68199	1.80957	-0.61950
C	-1.60941	5.25270	-0.96155
C	-2.09127	5.81823	-2.14158
H	-1.47108	5.82893	-0.03192
H	-2.64475	5.55129	-4.23983
H	5.59194	-2.04493	-4.59281
H	-5.48157	-5.03307	-1.20906
H	3.69801	-1.70414	-6.17411
H	-5.10405	-4.08908	-3.48282
H	-2.35075	6.89038	-2.15943
Cl	5.72053	-1.43713	0.91170
Cl	-3.00848	-3.72120	3.59834
H	3.30067	-3.57347	2.69761
H	0.64125	-4.27103	3.51541
H	2.86357	3.92998	2.78484
H	-2.41966	2.55069	4.37446
H	-3.17300	-0.20771	4.60474
H	4.77707	1.87465	2.19558
O	-1.50195	-0.58003	-5.40704
O	-1.56735	0.40818	-4.62377

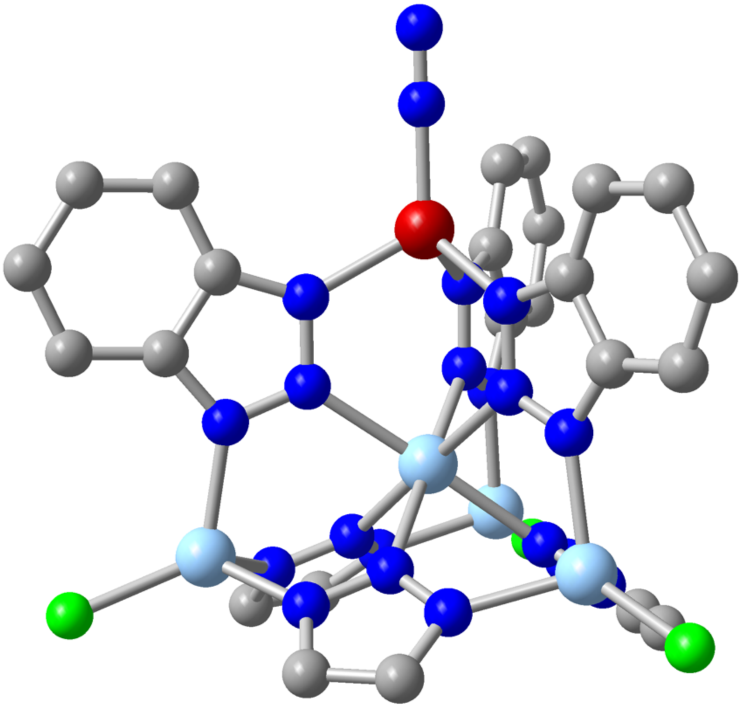

N_2 Coordination to Cu^{I}Zn-MFU-4*l* with Simplified Cluster Model.

N	2.08218	-2.70403	-0.25898
C	2.16615	-4.05370	-0.14880
N	1.12790	0.65615	-3.23335
C	2.23923	0.69119	-4.01134
N	-2.22752	-2.08078	-1.69440
C	-3.03232	-2.20381	-2.77965
N	0.04457	-3.44202	-0.08979
C	0.85649	-4.52817	-0.04001
N	0.79774	-2.35004	-0.22057
N	-1.59417	0.20267	2.27032
C	-2.52967	-0.37643	3.09236
C	-3.14386	0.05968	4.29218
C	-4.07235	-0.80402	4.87365
H	-2.89945	1.03460	4.74252
N	2.85470	0.02229	0.98977
C	3.50772	0.61724	2.03582
C	4.85710	0.55473	2.46219
C	5.18370	1.30183	3.59345
H	5.59108	-0.05886	1.91438
N	-1.01142	2.34788	-1.78275
C	-1.43477	3.62728	-1.54235
C	-1.86837	4.65320	-2.41689
C	-2.23842	5.85816	-1.82043
H	-1.90252	4.48308	-3.50569
N	1.34586	1.23217	2.05306

C	2.53312	1.39948	2.72263
N	1.57642	0.40974	1.03279
C	2.88146	2.15411	3.86943
C	4.21120	2.08903	4.28609
H	2.13679	2.76306	4.40578
N	2.71863	-0.28634	-2.09006
C	3.26145	0.08538	-3.27662
N	1.43294	0.06507	-2.07794
Zn	3.51335	-1.24557	-0.45983
Cu	-0.54834	1.95913	2.30090
N	-0.92236	2.62795	0.40464
C	-1.37681	3.80923	-0.12920
N	-0.71689	1.78734	-0.60618
C	-1.75496	5.04039	0.46083
C	-2.18228	6.04818	-0.40412
H	-1.71468	5.19320	1.55078
Zn	-0.79193	1.32168	-3.52734
N	-1.77910	-0.39775	-2.99479
C	-2.74442	-1.12187	-3.61555
N	-1.47927	-0.98732	-1.83747
Zn	-1.99669	-3.23297	-0.01213
Cl	-3.21332	-5.04027	0.18734
Zn	0.03575	-0.26741	-0.38241
N	-2.09959	-1.75466	1.38371
C	-2.85571	-1.64088	2.51962
N	-1.36516	-0.64426	1.26934
C	-3.79765	-2.51554	3.11474
C	-4.39306	-2.07199	4.29510
H	-4.03181	-3.48811	2.65161
H	-4.57682	-0.50525	5.80823
H	6.22096	1.28847	3.96926
H	-2.58499	6.69164	-2.45472
H	4.52635	2.66032	5.17577
H	-2.48745	7.02309	0.01273
H	-5.13444	-2.71361	4.80084
Cl	5.61262	-1.85723	-0.54376
Cl	-1.30345	2.25789	-5.43825
H	4.31725	-0.10685	-3.50508
H	2.22074	1.13700	-5.01374
H	0.44741	-5.54049	0.06822
H	-3.73696	-3.03944	-2.87481
H	-3.14831	-0.81772	-4.58934
H	3.13651	-4.56564	-0.15508
N	-1.04204	3.86456	4.54613
N	-0.85451	3.14408	3.70059

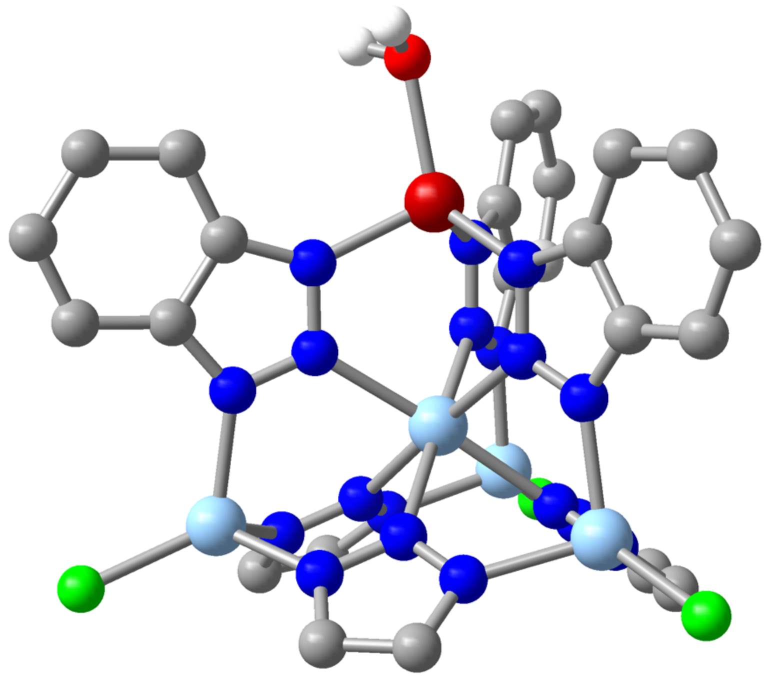

H_2O Coordination to $Cu^{I}Zn$-MFU-4*l* with Simplified Cluster Model.

N	2.64995	0.23644	2.12343
C	3.33178	1.06767	2.95118
N	-0.92547	-2.62025	2.00266
C	-0.46338	-3.69217	2.69517
N	-1.61107	1.90513	2.33762
C	-2.69465	1.98155	3.15068
N	1.47431	2.05713	2.28262
C	2.57623	2.23843	3.05289
N	1.53232	0.84666	1.72768
N	-0.13330	1.99308	-2.05455
C	-0.17785	3.33110	-2.36010
C	-0.19708	4.01939	-3.59686
C	-0.24494	5.41287	-3.54405
H	-0.16701	3.47201	-4.55311
N	2.73518	-1.35596	-0.52809
C	3.50478	-1.61682	-1.62875
C	4.78792	-2.20074	-1.76813
C	5.28627	-2.30086	-3.06721
H	5.34744	-2.54600	-0.88336
N	-2.62094	-1.63907	-0.40392
C	-3.38872	-2.01018	-1.47389
C	-4.59060	-2.75471	-1.56338
C	-5.11085	-2.94671	-2.84382
H	-5.07339	-3.15213	-0.65546
N	1.57751	-0.65267	-2.27804
C	2.76022	-1.16026	-2.75666

N	1.60010	-0.78179	-0.95220
C	3.28101	-1.26514	-4.06909
C	4.54594	-1.83976	-4.20084
H	2.71095	-0.89521	-4.93683
N	1.24397	-2.50963	1.94511
C	0.93129	-3.62079	2.65837
N	0.11570	-1.91709	1.55623
Zn	3.04924	-1.66409	1.45161
Cu	-0.08152	0.23481	-2.97927
N	-1.61870	-0.82262	-2.20165
C	-2.74266	-1.48365	-2.63148
N	-1.58151	-0.93605	-0.87463
C	-3.28579	-1.68658	-3.92271
C	-4.46911	-2.42103	-4.00836
H	-2.78826	-1.27383	-4.81540
Zn	-2.82616	-1.96205	1.59258
N	-2.60022	-0.02673	2.23722
C	-3.33088	0.73952	3.08603
N	-1.56728	0.68963	1.79298
Zn	-0.13886	3.26355	1.88858
Cl	-0.23222	5.22876	2.85249
Zn	-0.00312	-0.03181	0.36197
N	-0.16133	3.09421	-0.13545
C	-0.19788	4.04048	-1.12289
N	-0.12294	1.89083	-0.72446
C	-0.24704	5.45546	-1.07362
C	-0.27115	6.11910	-2.30052
H	-0.26387	5.98366	-0.10612
H	-0.26000	5.99107	-4.48369
H	6.28303	-2.74542	-3.22956
H	-6.04541	-3.52017	-2.96644
H	4.99522	-1.93856	-5.20382
H	-4.92693	-2.60105	-4.99593
H	-0.30937	7.22166	-2.31707
Cl	4.86738	-2.64600	2.17828
Cl	-4.50874	-3.13018	2.36873
H	1.71373	-4.26477	3.07903
H	-1.15094	-4.41292	3.15533
H	2.73490	3.17208	3.60715
H	-2.92560	2.89964	3.70550
H	-4.23289	0.34814	3.57291
H	4.28718	0.76669	3.39897
O	-0.20834	-0.02442	-5.13401
H	-0.95331	0.42401	-5.59148
H	-0.29688	-0.97843	-5.35268

12. References

(1) Denysenko, D.; Grzywa, M.; Tonigold, M.; Streppel, B.; Krkljus, I.; Hirscher, M.; Mugnaioli, E.; Kolb, U.; Hanss, J.; Volkmer, D. Elucidating Gating Effects for Hydrogen Sorption in MFU-4-Type Triazolate-Based Metal–Organic Frameworks Featuring Different Pore Sizes. *Chem. Eur. J.* **2011**, *17*, 1837–1848.

(2) Barnett, B. R.; Evans, H. A.; Su, G. M.; Jiang, H. Z. H.; Chakraborty, R.; Banyeretse, D.; Hartman, T. J.; Martinez, M. B.; Trump, B. A.; Tarver, J. D.; Dods, M. N.; Funke, L. M.; Börgel, J.; Reimer, J. A.; Drisdell, W. S.; Hurst, K. E.; Gennett, T.; FitzGerald, S. A.; Brown, C. M.; Head-Gordon, M.; Long, J. R. Observation of an Intermediate to H_2 Binding in a Metal–Organic Framework. *J. Am. Chem. Soc.* **2021**, *143*, 14884–14894.

(3) Myers, A. L.; Prausnitz, J. M. Thermodynamics of Mixed-Gas Adsorption. *AIChE J.* **1965**, *11*, 121–127.

(4) Sirita, J.; Phanichphant, S.; Meunier, F. C. Quantitative Analysis of Adsorbate Concentrations by Diffuse Reflectance FT-IR. *Anal. Chem.* **2007**, *79*, 3912–3918.

(5) Cliff, G.; Lorimer, G. W. The Quantitative Analysis of Thin Specimens. *J. Microsc.* **1975**, *103*, 203–207.

(6) Jaramillo, D. E.; Reed, D. A.; Jiang, H. Z.; Oktawiec, J.; Mara, M. W.; Forse, A. C.; Lussier, D. J.; Murphy, R. A.; Cunningham, M.; Colombo, V. Selective Nitrogen Adsorption via Backbonding in a Metal–Organic Framework with Exposed Vanadium Sites. *Nat. Mater.* **2020**, *19*, 517–521.

(7) Macrae, C. F.; Sovago, I.; Cottrell, S. J.; Galek, P. T. A.; McCabe, P.; Pidcock, E.; Platings, M.; Shields, G. P.; Stevens, J. S.; Towler, M.; Wood, P. A. Mercury 4.0: From Visualization to Analysis, Design and Prediction. *J. Appl. Crystallogr.*, **2020**, *53*, 226–235.

(8) Liu, Q.; Cho, S. G.; Hilliard, J.; Wang, T.-Y.; Chien, S.-C.; Lin, L.-C.; Co, A. C.; Wade, C. R. Inverse CO_2/C_2H_2 Separation with MFU-4 and Selectivity Reversal via Postsynthetic Ligand Exchange. *Angew. Chem. Int. Ed.* **2023**, *62*, e202218854.

(9) Siegelman, R. L.; Thompson, J. A.; Mason, J. A.; McDonald, T. M.; Long, J. R. A Cooperative Adsorbent for the Switch-like Capture of Carbon Dioxide from Crude Natural Gas. *Chem. Sci.* **2022**, *13*, 11772–11784.

(10) Denysenko, D.; Grzywa, M.; Jelic, J.; Reuter, K.; Volkmer, D. Scorpionate-Type Coordination in MFU-4l Metal–Organic Frameworks: Small-Molecule Binding and Activation upon the Thermally Activated Formation of Open Metal Sites. *Angew. Chem. Int. Ed.* **2014**, *53*, 5832–5836.

(11) Doud, E. A.; Inkpen, M. S.; Lovat, G.; Montes, E.; Paley, D. W.; Steigerwald, M. L.; Vázquez, H.; Venkataraman, L.; Roy, X. In Situ Formation of N-Heterocyclic Carbene-Bound Single-Molecule Junctions. *J. Am. Chem. Soc.* **2018**, *140*, 8944–8949.

(12) Denysenko, D.; Jelic, J.; Reuter, K.; Volkmer, D. Postsynthetic Metal and Ligand Exchange in MFU-4*l*: A Screening Approach toward Functional Metal–Organic Frameworks Comprising Single-Site Active Centers. *Chem. Eur. J.* **2015**, *21*, 8188–8199.

(13) Evans, A. D.; Cummings, M. S.; Luebke, R.; Brown, M. S.; Favero, S.; Attfield, M. P.; Siperstein, F.; Fairen-Jimenez, D.; Hellgardt, K.; Purves, R.; Law, D.; Petit, C. Screening Metal–Organic Frameworks for Dynamic CO/N_2 Separation Using Complementary Adsorption Measurement Techniques. *Ind. Eng. Chem. Res.* **2019**, *58*, 18336–18344.

(14) Wright, A. M.; Sun, C.; Dincă, M. Thermal Cycling of a MOF-Based NO Disproportionation Catalyst. *J. Am. Chem. Soc.* **2021**, *143*, 681–686.

(15) Mohamed, M. H.; Yang, Y.; Li, L.; Zhang, S.; Ruffley, J. P.; Jarvi, A. G.; Saxena, S.; Veser, G.; Johnson, J. K.; Rosi, N. L. Designing Open Metal Sites in Metal–Organic Frameworks for Paraffin/Olefin Separations. *J. Am. Chem. Soc.* **2019**, *141* (33), 13003–13007.
(16) Mian, M. R.; Chen, H.; Cao, R.; Kirlikovali, K. O.; Snurr, R. Q.; Islamoglu, T.; Farha, O. K. Insights into Catalytic Hydrolysis of Organophosphonates at M–OH Sites of Azolate-Based Metal Organic Frameworks. *J. Am. Chem. Soc.* **2021**, *143*, 9893–9900.
(17) Yan, X.; Song, Y.; Wang, D.; Xia, T.; Tan, X.; Ba, J.; Tang, T.; Luo, W.; Sang, G.; Xiong, R. Direct Observation of Highly Effective Hydrogen Isotope Separation at Active Metal Sites by in Situ DRIFT Spectroscopy. *Chem. Commun.* **2023**, *59*, 3922–3925.
(18) Zhu, H.-L.; Huang, J.-R.; Zhang, X.-W.; Wang, C.; Huang, N.-Y.; Liao, P.-Q.; Chen, X.-M. Highly Efficient Electroconversion of CO_2 into CH_4 by a Metal–Organic Framework with Trigonal Pyramidal Cu(I)N3 Active Sites. *ACS Catal.* **2021**, *11*, 11786–11792.
(19) FitzGerald, S. A.; Mukasa, D.; Rigdon, K. H.; Zhang, N.; Barnett, B. R. Hydrogen Isotope Separation within the Metal–Organic Framework Cu(I)-MFU-4*l*. *J. Phys. Chem. C* **2019**, *123*, 30427–30433.
(20) Bloch, E. D.; Queen, W. L.; Hudson, M. R.; Mason, J. A.; Xiao, D. J.; Murray, L. J.; Flacau, R.; Brown, C. M.; Long, J. R. Hydrogen Storage and Selective, Reversible O_2 Adsorption in a Metal–Organic Framework with Open Chromium(II) Sites. *Angew. Chem. Int. Ed.* **2016**, *55*, 8605–8609. https://doi.org/10.1002/anie.201602950.
(21) Murray, L. J.; Dinca, M.; Yano, J.; Chavan, S.; Bordiga, S.; Brown, C. M.; Long, J. R. Highly-Selective and Reversible O_2 Binding in Cr_3(1,3,5-benzenetricarboxylate)$_2$. *J. Am. Chem. Soc.* **2010**, *132*, 7856–7857.
(22) Oktawiec, J.; Jiang, H. Z. H.; Vitillo, J. G.; Reed, D. A.; Darago, L. E.; Trump, B. A.; Bernales, V.; Li, H.; Colwell, K. A.; Furukawa, H.; Brown, C. M.; Gagliardi, L.; Long, J. R. Negative Cooperativity upon Hydrogen Bond-Stabilized O_2 Adsorption in a Redox-Active Metal–Organic Framework. *Nat. Commun.* **2020**, *11*, 3087.
(23) Rosen, A. S.; Mian, M. R.; Islamoglu, T.; Chen, H.; Farha, O. K.; Notestein, J. M.; Snurr, R. Q. Tuning the Redox Activity of Metal–Organic Frameworks for Enhanced, Selective O2 Binding: Design Rules and Ambient Temperature O_2 Chemisorption in a Cobalt–Triazolate Framework. *J. Am. Chem. Soc.* **2020**, *142*, 4317–4328.
(24) Gallagher, A. T.; Lee, J. Y.; Kathiresan, V.; Anderson, J. S.; Hoffman, B. M.; Harris, T. D. A Structurally-Characterized Peroxomanganese(IV) Porphyrin from Reversible O_2 Binding within a Metal–Organic Framework. *Chem. Sci.* **2018**, *9*, 1596–1603.
(25) Mason, J. A.; Darago, L. E.; Lukens, W. W. Jr.; Long, J. R. Synthesis and O_2 Reactivity of a Titanium(III) Metal–Organic Framework. *Inorg. Chem.* **2015**, *54*, 10096–10104.
(26) Jaffe, A.; Ziebel, M. E.; Halat, D. M.; Biggins, N.; Murphy, R. A.; Chakarawet, K.; Reimer, J. A.; Long, J. R. Selective, High-Temperature O2 Adsorption in Chemically Reduced, Redox-Active Iron-Pyrazolate Metal–Organic Frameworks. *J. Am. Chem. Soc.* **2020**, *142*, 14627–14637.
(27) Krishna, R.; van Baten, J. M. How Reliable Is the Ideal Adsorbed Solution Theory for the Estimation of Mixture Separation Selectivities in Microporous Crystalline Adsorbents? *ACS Omega* **2021**, *6*, 15499–15513.
(28) Kreider-Mueller, A.; Quinlivan, P. J.; Rauch, M.; Owen, J. S.; Parkin, G. Synthesis, Structure and Reactivity of [Tm^{Bu^t}]ZnH, a Monomeric Terminal Zinc Hydride Compound in a Sulfur-Rich Coordination Environment: Access to a Heterobimetallic Compound. *Chem Commun* **2016**, *52*, 2358–2361.

(29) Sarangi, R.; Aboelella, N.; Fujisawa, K.; Tolman, W. B.; Hedman, B.; Hodgson, K. O.; Solomon, E. I. X-Ray Absorption Edge Spectroscopy and Computational Studies on $LCuO_2$ Species: Superoxide−Cu^{II} versus Peroxide−Cu^{III} Bonding. *J. Am. Chem. Soc.* **2006**, *128*, 8286–8296.
(30) Epifanovsky, E. *et al.*, Software for the Frontiers of Quantum Chemistry: An Overview of Developments in the Q-Chem 5 Package. *J. Chem. Phys.* **2021**, *155*, 084801.
